# Monoclonal Antibody for the Prevention of Respiratory Syncytial Virus in Infants and Children: A Systematic Review and Network Meta-analysis

**DOI:** 10.1001/jamanetworkopen.2023.0023

**Published:** 2023-02-17

**Authors:** Mingyao Sun, Honghao Lai, Feiyang Na, Sheng Li, Xia Qiu, Jinhui Tian, Zhigang Zhang, Long Ge

**Affiliations:** 1 Department of Intensive Care Unit, The First Hospital of Lanzhou University, Lanzhou, Gansu, China; 2 Evidence-Based Nursing Center, School of Nursing, Lanzhou University, Lanzhou, Gansu, China; 3 Evidence-Based Social Science Research Center, School of Public Health, Lanzhou University, Lanzhou, Gansu, China; 4 Department of Social Medicine and Health Management, School of Public Health, Lanzhou University, Lanzhou, Gansu, China; 5 Department of Pediatrics, Gansu Provincial Maternity and Child Care Hospital, Lanzhou, Gansu, China; 6 The First People’s Hospital of Lanzhou City, Lanzhou, Gansu, China; 7 Department of Pediatrics, West China Second University Hospital, Chengdu, Sichuan, China; 8 Key Laboratory of Obstetric & Gynecologic and Pediatric Diseases and Birth Defects of Ministry of Education, Sichuan University, Chengdu, Sichuan, China; 9 Evidence-Based Medicine Center, School of Basic Medical Sciences, Lanzhou University, Lanzhou, Gansu, China; 10 Key Laboratory of Evidence-Based Medicine and Knowledge Translation of Gansu Province, Lanzhou, Gansu, China

## Abstract

**Importance:**

Respiratory syncytial virus (RSV) is the leading cause of acute lower respiratory infection in children younger than 5 years; effective prevention strategies are urgently needed.

**Objective:**

To compare the efficacy and safety of monoclonal antibodies for the prevention of RSV infection in infants and children.

**Data Sources:**

In this systematic review and network meta-analysis, PubMed, Embase, CENTRAL, and ClinicalTrials.gov were searched from database inception to March 2022.

**Study Selection:**

Randomized clinical trials that enrolled infants at high risk of RSV infection to receive a monoclonal antibody or placebo were included. Keywords and extensive vocabulary related to monoclonal antibodies, RSV, and randomized clinical trials were searched.

**Data Extraction and Synthesis:**

The Preferred Reporting Items for Systematic Reviews and Meta-analyses reporting guideline was used. Teams of 2 reviewers independently performed literature screening, data extraction, and risk of bias assessment. The Grading of Recommendations, Assessments, Developments, and Evaluation approach was used to rate the certainty of evidence. A random-effects model network meta-analysis was conducted using a consistency model under the frequentist framework.

**Main Outcomes and Measures:**

The main outcomes were all-cause mortality, RSV-related hospitalization, RSV-related infection, drug-related adverse events, intensive care unit admission, supplemental oxygen use, and mechanical ventilation use.

**Results:**

Fifteen randomized clinical trials involving 18 395 participants were eligible; 14 were synthesized, with 18 042 total participants (median age at study entry, 3.99 months [IQR, 3.25-6.58 months]; median proportion of males, 52.37% [IQR, 50.49%-53.85%]). Compared with placebo, with moderate- to high-certainty evidence, nirsevimab, palivizumab, and motavizumab were associated with significantly reduced RSV-related infections per 1000 participants (nirsevimab: −123 [95% CI, −138 to −100]; palivizumab: −108 [95% CI, −127 to −82]; motavizumab: −136 [95% CI, −146 to −125]) and RSV-related hospitalizations per 1000 participants (nirsevimab: −54 [95% CI, −64 to −38; palivizumab: −39 [95% CI, −48 to −28]; motavizumab: −48 [95% CI, −58 to −33]). With moderate-certainty evidence, both motavizumab and palivizumab were associated with significant reductions in intensive care unit admissions per 1000 participants (−8 [95% CI, −9 to −4] and −5 [95% CI, −7 to 0], respectively) and supplemental oxygen use per 1000 participants (−59 [95% CI, −63 to −54] and −55 [95% CI, −61 to −41], respectively), and nirsevimab was associated with significantly reduced supplemental oxygen use per 1000 participants (−59 [95% CI, −65 to −40]). No significant differences were found in all-cause mortality and drug-related adverse events. Suptavumab did not show any significant benefits for the outcomes of interest.

**Conclusions and Relevance:**

In this study, motavizumab, nirsevimab, and palivizumab were associated with substantial benefits in the prevention of RSV infection, without a significant increase in adverse events compared with placebo. However, more research is needed to confirm the present conclusions, especially for safety and cost-effectiveness.

## Key Points

**Question:**

What is the most appropriate monoclonal antibody (mAb) for the prevention of respiratory syncytial virus (RSV) in infants and children?

**Findings:**

In this systematic review and network meta-analysis of 14 randomized clinical trials assessing efficacy and safety of 4 mAbs, nirsevimab, motavizumab, and palivizumab were associated with significant reductions in RSV-related hospitalization, infection, and supplemental oxygen use. Motavizumab was associated with a significantly larger reduction in RSV infection, intensive care unit admissions, and mechanical ventilation use than palivizumab.

**Meaning:**

The findings suggest that motavizumab, nirsevimab, and palivizumab are associated with substantial benefits in the prevention of RSV infection in infants and children.

## Teaser

This systematic review and network meta-analysis compares the efficacy and safety of 4 monoclonal antibodies for preventing respiratory syncytial virus among infants and children.

## Introduction

Respiratory syncytial virus (RSV) is a leading cause of respiratory disease in children worldwide and is also the primary cause of hospitalization for viral respiratory infections and a major cause of overall mortality in infants and children, especially premature infants.^1,2^ With an estimated 2.1 million children younger than 5 years requiring inpatient or outpatient treatment for RSV infection each year,^3,4^ the overall mortality burden of RSV disease worldwide is 1 in 28 deaths among infants aged 28 days to 6 months.^5^ As most RSV deaths occur in the community and may be missed in hospital surveillance, the actual situation may be worse.^6,7^ Therefore, effective RSV prevention strategies are urgently needed to address this major public health issue and burden.^8^

Respiratory syncytial virus passive immunization programs targeting protection during the first 6 months of life could substantially reduce RSV disease burden.^5^ The World Health Organization published documents in 2021 that already encouraged the development of preventive interventions for RSV.^9^ Palivizumab is currently the most widely used prophylaxis for preventing RSV disease in infants. Two meta-analyses of randomized clinical trials (RCTs) suggested that palivizumab could significantly reduce RSV-related hospitalizations by 51 to 55 per 1000 participants (baseline risk: 98-101 per 1000 participants) compared with placebo.^10,11^ Although the efficacy of palivizumab has been proved, it is not available in some countries, such as China. Meanwhile, the high price of palivizumab imposes a substantial economic burden on low- and middle-income families.^12^ Therefore, new monoclonal antibodies (mAbs) have been developed, such as nirsevimab, which could protect infants from RSV-related infection and hospitalization during an entire RSV season with a single dose. Nirsevimab was assessed in 2 large, multicenter RCTs conducted in the northern and southern hemispheres, with strong practicability.^13,14^ In addition to palivizumab and nirsevimab, other mAbs were also under consideration, such as motavizumab, which could significantly shorten the length of RSV hospitalization.^15,16^ However, the relative efficacy and safety of different mAbs have not been compared comprehensively. Therefore, we performed a systematic review and network meta-analysis of RCTs to assess the benefits and harms of different mAbs for the prevention of RSV infection among infants and children.

## Methods

The protocol for this systematic review and network meta-analysis was registered on PROSPERO (CRD42022322043). We followed the Preferred Reporting Items for Systematic Reviews and Meta-analyses (PRISMA) reporting guideline to perform the study and report the present results.^17^

### Search Strategy and Selection Criteria

To identify eligible studies, we searched PubMed, Embase, CENTRAL, and ClinicalTrials.gov from database inception up to March 2022. We used a sensitive search strategy with keywords and extensive vocabulary related to monoclonal antibodies, respiratory syncytial virus, and RCTs (eTable 1 in Supplement 1). The reference lists of relevant studies were also tracked to identify eligible studies.

Eligible studies were RCTs that enrolled infants at high risk of RSV infection (eg, preterm infants, geographic conditions, infants younger than 6 months, and infants with chronic lung disease of prematurity or congenital heart disease) using any type of mAbs or alternative mAbs. For studies that only reported laboratory metrics, we discussed the relevant effects on our findings rather than including them in data synthesis.

The search records were independently reviewed by teams of 2 reviewers (M.Y.S. and H.H.L. or F.Y.N. and X.Q.) using an online platform, Rayyan,^18^ through titles and abstracts of the articles to identify the potential trials. Then, full texts of the potential trials were obtained and screened independently under the eligibility criteria. Any differences were resolved by discussion among all authors.

### Data Extraction

Two reviewers (M.Y.S., H.H.L.) independently collected the data of interest from all the studies using a data extraction sheet; any conflict was resolved by discussion. The main information included general information (author, year, sample size, follow-up time, and funding), characteristics of participants (country, age, sex, race or ethnicity, and high-risk factors), details of intervention (types of mAb, dosage, and route), and outcomes. Race and ethnicity categories included Asian, White, Black, Hispanic, or other (reported as “other” in study); we collected this information to assess whether there were any differences in use of mAbs between different races or ethnicities.

### Outcomes

We focused on the patient-important outcomes, including all-cause mortality, rate and duration of RSV-related hospitalization, rate of RSV infection, drug-related adverse events or serious adverse events, rate and duration of intensive care unit (ICU) admission, rate and duration of supplemental oxygen use, and rate and duration of mechanical ventilation (MV) use. The rate of RSV-related hospitalization was defined as the number of participants hospitalized with laboratory-confirmed RSV-related infections. A drug-related adverse event was defined as any unexpected or harmful occurrence in the participant related to the trial drug. The rate of RSV infection was defined as a positive polymerase chain reaction test result and RSV-associated medically attended lower respiratory tract infection.

### Risk of Bias Assessment

The risk of bias assessment of individual studies was evaluated independently by 2 reviewers (M.Y.S., H.H.L.) using the revised Cochrane risk of bias tool for randomized trials,^19^ which addressed the following domains: bias from the randomization process generated, deviations from the intended intervention, missing data (we judged high risk of bias if the rate of missing data was >10%), measurement of the outcome, selection of the reported results, and overall bias. Each signaling question within each domain of bias was judged as “yes,” “probably yes,” “no,” “probably no,” or “no information” and finally judged as “low,” “some concerns,” and “high.”

### Assessment of Certainty of Evidence

Two trained methodologists (M.Y.S., H.H.L.) independently rated the certainty (quality) of the evidence using the Grading of Recommendations, Assessments, Developments, and Evaluation (GRADE) framework, which rates evidence as high, moderate, low, or very low certainty.^20^ We first rated the certainty of evidence for each direct comparison according to the GRADE framework for pairwise meta-analyses. Then we rated the certainty of indirect evidence, focusing on the dominant first-order loops,^21^ rating the certainty of indirect evidence as the lowest certainty among direct comparisons that dominated the loop. In the absence of first-order loops, we used the same method but assessed higher-order loops to rate the certainty of evidence. Finally, we rated the certainty of the network estimation. We started with the certainty of direct or indirect evidence that dominated the comparison for network estimates and then considered rating down the certainty in network estimates due to inconsistency between direct and indirect estimates. If there was inconsistency or imprecision between direct and indirect estimates, we considered reducing the certainty of the network estimates.^22^

### Statistical Analysis

We calculated pooled odds ratios (ORs) with 95% CIs for dichotomous outcomes and pooled mean differences with 95% CIs for continuous outcomes. When quantitative data synthesis was impossible, we summarized the results qualitatively. For dichotomous outcomes, absolute effects were also calculated based on the pooled ORs and baseline risk (the median incidence rate in the placebo group).^23^

Pairwise meta-analyses were performed using RevMan, version 5.4.1.^24^ The data were synthesized with a random-effects model to obtain direct effect estimates for each pairwise comparison. Heterogeneity among trials within each comparison was evaluated using the *I*^2^ statistic.^25^ Publication bias was evaluated using funnel plots.

We conducted a random-effects model network meta-analysis using a consistency model with the netmeta, version 1.0-1, package under a frequentist framework in R, version 1.3.1093 (R Project for Statistical Computing)^26^ (eMethods in Supplement 1); the estimator was based on weighted least-squares regression with the Moore-Penrose pseudoinverse method.^27^ To assess the inconsistency between direct and indirect estimates and obtain indirect effect estimates, the netsplit function of R was used. *P* values were used to rank each intervention with the interpretation of the mean extent of certainty that one intervention was better than the other.^28^ Two-sided *P* < .05 was considered significant.

To explore the sources of heterogeneity, we conducted the subgroup analysis with direct comparisons for a priori–specified variables including health status (with vs without comorbidities, with a predefined hypothesis of a larger effect in participants with comorbidities including bronchopulmonary dysplasia, congenital heart disease, or chronic lung disease at baseline). We also performed metaregression analyses for gestational age and the proportion of males. In addition, we performed sensitivity analyses to observe the robustness of results by repeating the analyses using a bayesian hierarchical model^29,30^ and both a fixed-effects model and a random-effects model.

## Results

**Figure 1.**
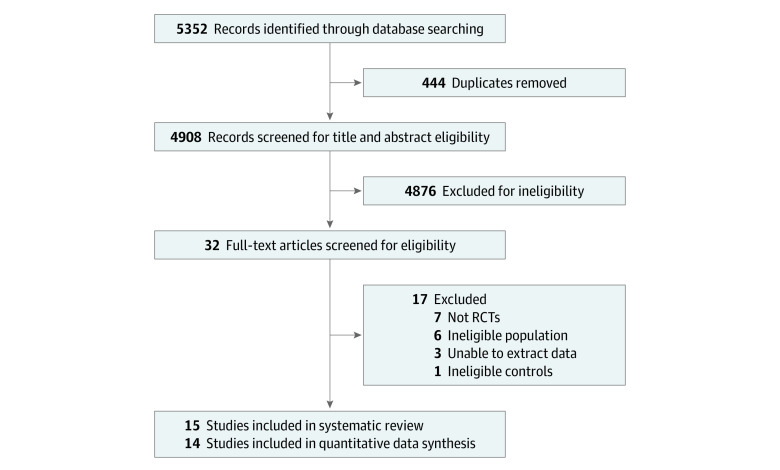
PRISMA Flow Diagram RCTs indicates randomized clinical trials.

**Figure 2.**
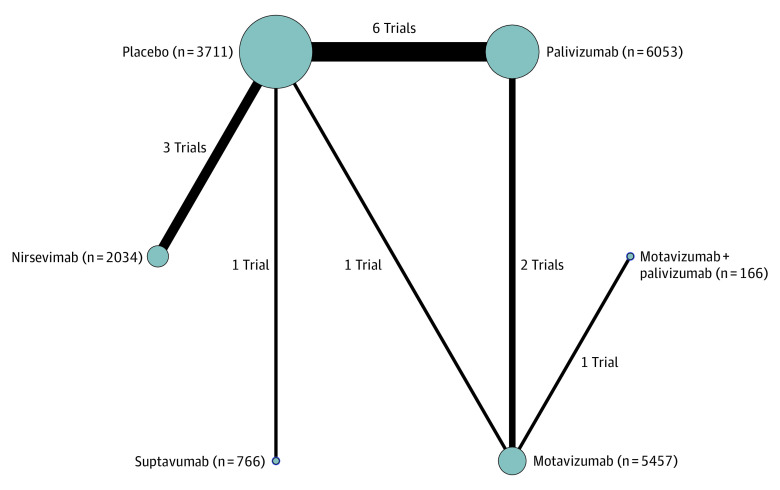
Network Plot of All Included Studies Network plot comparing monoclonal antibodies for preventing respiratory syncytial virus in children. The line width is proportional to the number of studies comparing each pair of interventions, and the size of each node is proportional to the number of participants (sample size).

Our search yielded 5352 records; after removing duplicates, 4908 records were screened. Finally, 15 RCTs^13,14,31,32,33,34,35,36,37,38,39,40,41,42,43^ involving 18 395 participants proved to be eligible (Figure 1 shows the PRISMA flow diagram, and eTable 2 in Supplement 1 shows the list of excluded studies). Of these RCTs, 14 were able to be synthesized and involved 4 mAbs (nirsevimab, motavizumab, palivizumab, and suptavumab),^13,14,32,33,34,35,36,37,38,39,40,41,42,43^ with a total of 18 042 participants. Figure 2 shows a network plot of all included studies; the comparison of palivizumab and placebo was the most common.

Detailed baseline information for all included studies is listed in Table 1 and eTables 3 and 4 in Supplement 1. Participants were from 32 countries in the northern and southern hemispheres, with a median age at entry of 3.99 months (IQR, 3.25-6.58 months), a median gestational age of 33.1 weeks (IQR, 31.1-35 weeks), a median proportion of females of 47.63% (IQR, 46.15%-49.51%), and a median proportion of males of 52.37% (IQR, 50.49%-53.85%). The median proportion of Asian participants was 1.40% (IQR, 1.16%-1.83%); Black participants, 13.21% (IQR, 8.55%-24.88%); Hispanic participants, 10.92% (IQR, 8.58%-11.11%); White participants, 70.86% (IQR, 53.50%-78.57%); and other racial and ethnic groups, 6.15% (IQR, 4.00%-8.63%). Of all participants, 8.08% were reported as having chronic lung disease at entry, 13.98% as having congenital heart disease at entry, 4.51% as having bronchopulmonary dysplasia at entry, and 66.88% as being born prematurely. The minimum follow-up time was 150 days, and the maximum was 3 to 6 years.

**Table 1.** Study Characteristics

Source	Population						Intervention (dose)/comparison
	Sample	Age at start, mean (SD), mo	Gestational age, mean (SD), wk	Males, %	Intervention sample, No./comparison sample, No	Follow-up time	
Griffin et al,^13^ 2020	Healthy preterm infants	3.29 (2.25)	32.7 (14.3)	52.37	969/484	150 d	Nirsevimab (1 dose of 50 mg)/placebo
O’Brien et al,^33^ 2015	Healthy infants	2.1 (1.91)	36	50.49	1417/710	150 d to 3 y	Motavizumab (5 doses of 15 mg/kg)/placebo
Subramanian et al,^34^ 1998	Preterm infants with or without BPD	6.78 (1.46)	NR	NR	(10, 10, or 22)/20	150 d	Palivizumab (5 doses of 3, 10, or 15 mg/kg)/placebo
Hammitt et al,^14^ 2022	Healthy late-preterm and term infants	2.59	NR	48.4	993/497	150-360 d	Nirsevimab (1 dose of 50 mg [<5 kg]/100 mg [≥5 kg])/placebo
Blanken et al,^35^ 2013	Healthy preterm infants	NR	34	51.05	214/215	1 y	Palivizumab (2-5 doses of 15 mg/kg)/placebo
Feltes et al,^36^ 2011	Children aged <24 mo with CHD	8.33 (6.46)	3.85 (2.05)	53.04	623/612	150 d	Motavizumab (5 doses of 15 mg/kg)/palivizumab (5 doses of 15 mg/kg)
Carbonell-Estrany et al,^37^ 2010	Preterm infants with CLD	3.99 (3.77)	31.1 (3.1)	54.66	3329/3306	150 d	Motavizumab (5 doses of 15 mg/kg)/palivizumab (5 doses of 15 mg/kg)
Feltes et al,^38^ 2003	Children aged ≤24 mo with CHD	6.65 (0.2)	38.5 (0.1)	53.85	639/648	150 d	Palivizumab (5 doses of 15 mg/kg)/placebo
Impact-RSV Study Group,^32^ 1998	Preterm infants with BPD	5.8 (0.17)	29 (0.11)	56.86	1002/500	150 d	Palivizumab (5 doses of 15 mg/kg)/placebo
Simões et al,^39^ 2021	Healthy preterm infants	3.2 (6.9)	NR	53.26	(386 or 383)/385	150-237 d	Suptavumab (1 or 2 doses of 30 mg)/placebo
Scheltema et al,^40^ 2018	Healthy preterm infants	NR	34	51.05	214/215	6 y	Palivizumab (NR)/placebo
Domachowske et al,^41^ 2018	Healthy preterm infants	6.5 (2.64)	33.1 (0.8)	40.8	(8, 31, or 33)/20	360 d	Nirsevimab (1 dose of 10, 25, or 50 mg)/placebo
Tavsu et al,^42^ 2014	Preterm infants and NICU children	NR	29.55 (17.1)	46.25	41/42	1-2 y	Palivizumab (5 doses of 15 mg/kg)/placebo
Fernández et al,^43^ 2010	Preterm infants with or without CLD	3.7 (2.6)	31.1 (2.7)	54.6	(83 or 84)/93	150 d	2 Doses motavizumab +3 doses palivizumab or 2 doses palivizumab +3 doses motavizumab/5 doses motavizumab (15 mg/kg each dose)

Abbreviations: BPD, bronchopulmonary dysplasia; CHD, congenital heart disease; CLD, chronic lung disease; NICU, neonatal intensive care unit; NR, not reported.

The risk of bias assessment is shown in eTables 5 and 6 and eFigure 1 in Supplement 1. Overall, for the dichotomous outcomes, 4 studies^39,41,42,43^ were judged as “some concerns,” mainly because of no information on the allocation concealment procedures. One study^42^ comparing palivizumab with no intervention reported that only physicians responsible for follow-up and neurodevelopmental examination were blinded to the interventions; therefore, we considered serious risk of bias in deviations from the intended intervention. For the continuous outcomes, 7 studies^33,37,38,39,41,42,43^ were judged as “some concerns,” mainly because of missing SDs.

**Figure 3.**
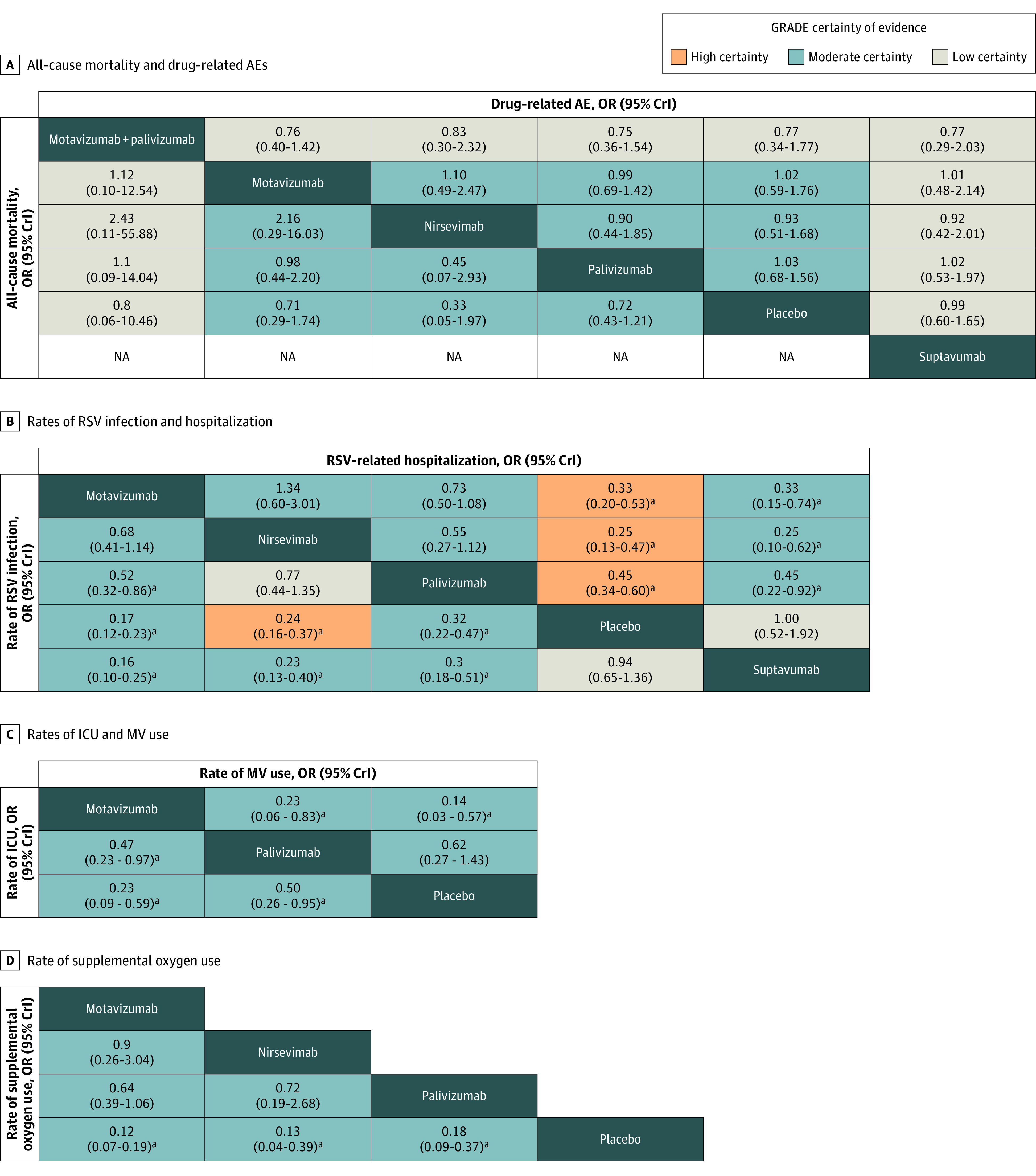
League Tables of Outcome Analyses The league tables report comparisons of the treatments in the column vs row in the left lower cells, and row vs column in the right upper cells. The estimates are odds ratios (ORs) with 95% credible intervals (CrIs) in common between the column and the row. In the left lower cells of the tables, ORs less than 1 favor the column-defining treatment, and in the upper right cells, the row-defining treatment. AE indicates adverse event; GRADE, Grading of Recommendations, Assessments, Developments, and Evaluation; ICU, intensive care unit; MV, mechanical ventilation; NA, not applicable; and RSV, respiratory syncytial virus. ^a^Statistically significant difference.

eFigures 2 to 9 in Supplement 1 present the network plots for each outcome; eFigures 10 to 44 in Supplement 1 present the forest plots of each pairwise comparison; eTables 7 to 20 in Supplement 1 present the league tables of network meta-analyses (a league table is a square matrix showing all pairwise comparisons in a network meta-analysis) (eResults in Supplement 1); eTable 21 in Supplement 1 presents the cumulative ranking of interventions for different outcomes; and eTables 22 to 29 in Supplement 1 present the certainty of evidence for each outcome of each comparison. The certainty of evidence was generally moderate, downgraded mainly because of serious risk of bias or serious imprecision. Figure 3 presents the league tables of the network estimates for all outcomes, and Table 2 summarizes the absolute effects of all outcomes. eTables 30 to 45 in Supplement 1 show the results of sensitivity analyses, eFigures 67 to 68 in Supplement 1 show the results of subgroup analyses (no significant subgroup differences were found), and eFigures 69 to 71 in Supplement 1 show the results of meta-regression. eFigure 72 in Supplement 1 shows the comparison of efficacy and safety outcomes of different mAbs. The present results were robust, and no serious heterogeneity was detected among all outcomes. Node-split plots are presented in eFigures 46 to 59 in Supplement 1, and contribute plots are presented in eFigures 60 to 66 in Supplement 1.

**Table 2.** Summary of Associations of Monoclonal Antibodies With Benefit and Harm Outcomes

Intervention	Estimated absolute difference, per 1000 participants (95% CI)^a^														
	Benefit outcomes												Safety outcomes		
	Rate of RSV infection		RSV-related hospitalization		Rate of ICU admission in all participants		Rate of supplemental oxygen use in all participants		Rate of MV use in all participants		Rate of wheezing		Drug-related AEs		All-cause mortality
**Compared with placebo**															
Placebo, No. per 1000 participants	170	74		10		6		3		279		43		5	
Nirsevimab	–123 (–138 to –100)^b^	–54 (–64 to –38)^b^		–10 (–10 to –2)^b^^,^^c^		–59 (–65 to –40)^b^		–3 (–3 to 9)^c^^,^^d^		NA		–3 (–21 to 27)^e^		–3 (–5 to 5)^e^	
Motavizumab	–136 (–146 to –125)^b^	–48 (–58 to –33)^b^		–8 (–9 to –4)^b^		–59 (–63 to –54)^b^		–3 (–3 to –1)^b^		–12 (–52 to 33)^c^^,^^f^		1 (–17 to 30)^e^		–1 (–4 to 4)^e^	
Palivizumab	–108 (–127 to –82)^d^	–39 (–48 to –28)^d^		–5 (–7 to 0)^d^		–55 (–61 to –42)^b^		–1 (–2 to 1)^f^		–111 (–172 to –27)^b^^,^^c^		1 (–13 to 23)^e^		–1 (–3 to 1)^e^	
Suptavumab	8 (–40 to 69)^g^	0 (–34 to 59)^g^		NA		NA		NA		NA		0 (–16 to 27)^f^		2 (–5 to 139)^c^^,^^f^	
Motavizumab plus palivizumab	NA	NA		NA		NA		NA		NA		–10 (–28 to 31)^f^		–1 (–5 to 45)^f^	
**Compared with other intervention**															
Nirsevimab vs palivizumab	–14 (–34 to 2)^h^	–15 (–25 to 4)^d^		NA		–4 (–11 to 21)^d^		NA		NA		–4 (–24 to 34)^e^		–2 (–4 to 8)^e^	
Nirsevimab vs suptavumab	–131 (–151 to –98)^b^	–54 (–66 to –27)^b^		NA		NA		NA		NA		–3 (–24 to 40)^f^		NA	
Motavizumab vs nirsevimab	–15 (–27 to 6)^d^	7 (–8 to 38)^d^		NA		–1 (–7 to 18)^d^		NA		NA		4 (–20 to 53)^e^		2 (–1 to 29)^e^	
Motavizumab vs palivizumab	–29 (–41 to –8)^b^	–9 (–17 to 3)^d^		–3 (–4 to 0)^b^		–5 (–8 to 1)^d^		–2 (–2 to 0)^b^		NA		0 (–13 to 17)^e^		0 (–2 to 5)^e^	
Motavizumab vs suptavumab	–145 (–157 to –127)^b^	–48 (–62 to –18)^b^		NA		NA		NA		NA		0 (–22 to 45)^f^		NA^i^	
Palivizumab vs suptavumab	–117 (–140 to –79)^b^	–39 (–57 to –6)^b^		NA		NA		NA		NA		1 (–20 to 38)^f^		NA^i^	
Motavizumab plus palivizumab vs nirsevimab	NA	NA		NA		NA		NA		NA		–7 (–28 to 48)^f^		3 (–2 to 99)^f^	
Motavizumab plus palivizumab vs motavizumab	NA	NA		NA		NA		NA		NA		–10 (–26 to 17)^i^		0 (–4 to 44)^i^	
Motavizumab plus palivizumab vs palivizumab	NA	NA		NA		NA		NA		NA		–11 (–28 to 22)^i^		0 (–4 to 49)^i^	
Motavizumab plus palivizumab vs suptavumab	NA	NA		NA		NA		NA		NA		–10 (–30 to 41)^i^		NA	

Abbreviations: AEs, adverse events; ICU, intensive care unit; MV, mechanical ventilation; NA not applicable; RSV, respiratory syncytial virus.

^a^ Compared with standard care. For the rates of ICU admission, supplemental oxygen use, and MV use, the included studies stated these analyses were done in all participants.

^b^ Better than placebo and some other interventions, with high or moderate certainty.

^c^ Obtained from direct evidence.

^d^ Better than placebo but no better than any other interventions, with high or moderate certainty.

^e^ No more harmful than placebo, with high or moderate certainty.

^f^ May be better than placebo and some alternatives or may be no more harmful than placebo, with low or very low certainty.

^g^ May be no better than placebo or may be more harmful than placebo and some alternatives, with low or very low certainty.

^h^ May be better than placebo but no better than other interventions or may be more harmful than placebo but no worse than other interventions, with low or very low certainty.

^i^ No better than placebo, with high or moderate certainty.

Nine RCTs^13,14,32,34,35,37,38,39,42^ involving 14 167 participants reported the rate of RSV-related hospitalization. Results showed that nirsevimab (OR, 0.25; 95% CI, 0.13-0.47; difference, −54 [95% CI, −64 to −38] per 1000 participants; high certainty), motavizumab (OR, 0.33; 95% CI, 0.20-0.53; difference, −48 [95% CI, −58 to −33] per 1000 participants; high certainty), and palivizumab (OR, 0.45; 95% CI, 0.34-0.60; difference, −39 [95% CI, −48 to −28] per 1000 participants; high certainty) were associated with significant reductions in the rate of hospitalization. Suptavumab showed no association with hospitalization. Among these mAbs, no statistically significant differences were found.

Seven RCTs^13,14,33,34,35,39,42^ involving 7030 participants reported the rate of RSV infection. Motavizumab (OR, 0.17; 95% CI, 0.12-0.23; difference, −136 [95% CI, −146 to −125] per 1000 participants; moderate certainty), nirsevimab (OR, 0.24; 95% CI, 0.16-0.37; difference, −123 [95% CI, −138 to −1000] per 1000 participants; high certainty), and palivizumab (OR, 0.32; 95% CI, 0.22-0.47; difference, −108 [95% CI, −127 to −82] per 1000 participants; moderate certainty) were significantly associated with reductions in the rate of RSV infection. Suptavumab showed no association with RSV infection. No significant differences were found between motavizumab and nirsevimab; however, a significantly larger reduction was found for motavizumab than for palivizumab (OR, 0.52; 95% CI, 0.32-0.86; difference, −29 [95% CI, −41 to −8] per 1000 participants; moderate certainty).

Three RCTs^13,33,37^ involving 10 209 participants reported the rate of supplemental oxygen use. Motavizumab (OR, 0.12; 95% CI, 0.07-0.19; difference, −59 [95% CI, −63 to −54] per 1000 participants; moderate certainty), nirsevimab (OR, 0.13; 95% CI, 0.04-0.39; difference, −59 [95% CI, −65 to −40] per 1000 participants; moderate certainty), and palivizumab (OR, 0.18; 95% CI, 0.09-0.37; difference, −55 [95% CI, −61 to −42] per 1000 participants; moderate certainty) all were associated with significant reductions in supplemental oxygen use compared with placebo. However, no significant differences were found among interventions.

Four RCTs^13,33,37,38^ involving 11 496 participants reported the rate of MV use and the rate of ICU admission. Compared with placebo, motavizumab was associated with significant reductions in both MV use (OR, 0.14; 95% CI, 0.03-0.57; difference, −3 [95% CI, −3 to −1] per 1000 participants; moderate certainty) and ICU admission (OR, 0.23; 95% CI, 0.09-0.59; difference, −8 [95% CI, −9 to −4] per 1000 participants; moderate certainty), while palivizumab was only associated with a reduction in ICU admissions (OR, 0.50; 95% CI, 0.26-0.95; difference, −5 [95% CI, −7 to 0] per 1000 participants; moderate certainty). Motavizumab was associated with a larger reduction in both outcomes than was palivizumab (MV use: OR, 0.23, 95% CI, 0.06-0.83; difference, −2 [95% CI, −2 to 0] per 1000 participants; moderate certainty; ICU admission: OR, 0.47; 95% CI, 0.23-0.97; difference, −3 [95% CI, −4 to 0] per 1000 participants; moderate certainty). The results for nirsevimab were obtained from direct evidence because the intervention group had no events, and results showed that nirsevimab was associated with a reduced rate of ICU admission (OR, 0.04; 95% CI, 0.00-0.81; difference, −10 [95% CI, −10 to −2] per 1000 participants; moderate certainty).

Eleven RCTs^13,14,32,33,34,36,37,38,39,41,43^ involving 17 203 participants reported drug-related adverse events or serious adverse events and all-cause mortality, including all 4 mAbs (only data on adverse events were available for suptavumab). One trial^13^ comparing nirsevimab with placebo reported no serious adverse events; moderate- to low-certainty evidence indicated that no significant differences were found in outcomes (Figure 3). The funnel plot was symmetric in distribution, which indicated there was no evidence of publication bias (eFigure 45 in Supplement 1).

Four continuous outcomes were reported in 4 RCTs^13,33,37,38^ involving 11 502 participants; since most studies did not report SDs, we did not synthesize the data. The duration of MV use, ICU admission, supplemental oxygen use, and RSV-related hospitalization were assessed with 3 mAbs, and the intervention group had a significantly shorter duration than the placebo group (eTable 46 in Supplement 1). The results of sensitivity analyses were similar to those of the primary analysis, indicating the robustness of our findings.

## Discussion

Our study compared the efficacy and safety of 4 mAbs (motavizumab, nirsevimab, palivizumab, and suptavumab) for the prevention of RSV infection in children using a network meta-analysis and GRADE approach. High to low certainty of evidence indicated that motavizumab, nirsevimab, and palivizumab were likely to be associated with reducing the rate of RSV-related hospitalizations compared with placebo; however, there were no significant differences among interventions. Evidence of high or moderate certainty showed that motavizumab, nirsevimab, and palivizumab were associated with reduced rates of RSV infection compared with placebo, with 108 to 136 fewer infections per 1000 participants. Motavizumab was associated with a larger reduction of infection than was palivizumab. With regard to the rate of RSV-related hospitalization and RSV infection, we did not find significant subgroup differences between patients with and without comorbidities. We also did not find significant subgroup differences for the proportion of males or gestational age. Moderate evidence showed that both motavizumab and palivizumab were associated with a significant reduction in the rate of ICU admissions and supplemental oxygen use, while motavizumab was associated with a larger reduction in ICU admissions than was palivizumab. Similarly, nirsevimab was associated with reduced supplemental oxygen use compared with placebo; however, there were no significant differences among interventions. In terms of the rate of MV use, in the nirsevimab group, no use of MV among participants was reported; only the results of motavizumab and palivizumab were synthesized, and moderate certainty of evidence showed that motavizumab was associated with reduced use of MV. None of the 4 mAbs showed a significant difference in safety outcomes compared with placebo, possibly because we did not analyze adverse events separately. However, significant skin reactions were reported with motavizumab use, which is the reason why it was not licensed.^3^ The results of 1 trial, conducted by Abarca and colleagues,^31^ were not synthesized; however, the sensitivity analysis showed that this trial did not affect the overall results.

### Comparison With Other Studies

Our review summarized the efficacy and safety of both approved and candidate mAbs to prevent RSV infection in children. In comparison with a Cochrane review,^11^ our review included 3 more mAbs (motavizumab, nirsevimab, and suptavumab) and 10 additional RCTs. Our results were similar to a previous study on palivizumab,^10^ which found that palivizumab was associated with reductions in the rate of RSV-related hospitalizations and RSV infections and with few to no differences in mortality and adverse events. In contrast, our systematic review and network meta-analyses added the results that palivizumab was associated with reduced rates of ICU admissions and supplemental oxygen use. We would have liked to have focused on wheezing; however, a previous review of recurrent childhood wheezing^44^ analyzed this in a comprehensive manner. Both American Academy of Pediatrics–published guidance for palivizumab prophylaxis in 2014^3^ and an expert consensus published in 2018^45^ recommended prophylaxis with palivizumab in preterm infants. Our review, by calculating the absolute differences in outcomes, showed similar results, which enhances the strength of the evidence. Motavizumab was not recommended by the American Academy of Pediatrics considering its lack of greater clinical efficacy and more hypersensitivity reactions noted by the US Food and Drug Administration. Since the Food and Drug Administration report, several large RCTs have been published; by summarizing all the available evidence, our review showed that motavizumab was associated with a significant reduction in the rate of RSV infection, RSV-related hospitalization, ICU admission, supplemental oxygen use, and MV use. Although motavizumab is currently unlicensed, the results of this review may shed some light on future preventive measures.

### Implications of the Study

During the review process, we found that insufficient evidence led to imprecision. In addition, the safety outcomes of mAbs were mostly no different from those of placebo. Future clinical studies could explore more about the safety of mAbs in different populations (eg, in southern and northern hemisphere populations). Considering the current status of the different mAbs, palivizumab currently remains the only licensed mAb with low use due to its high cost; motavizumab was not licensed; there are plans for regulatory approval for nirsevimab in 2022; and another half-life–extended monoclonal antibody, clesrovimab, is currently in phase 3 development. The analysis of phase 1b/2a study data showed that compared with placebo, a single dose of clesrovimab could provide 74.2% efficacy for the prevention of medically attended lower respiratory tract RSV infection for a duration of 5 months in infants. However, data on clesrovimab have currently only been presented at the 12th International RSV Symposium in 2022.^46^ Vaccines, mAbs, or other preventive interventions for RSV should be developed, including for use in pregnant individuals and especially for use in low- and middle-income countries.

### Strengths and Limitations

Strengths of our systematic review and network meta-analysis included a comprehensive search for all mAb prophylaxes against RSV; study selection, data extraction, and a rigorous risk of bias assessment performed independently by 2 reviewers; a focus on the differences in patient-important outcomes among patients with different health status, gestational age, and sex; and application of the GRADE framework to assess the certainty of evidence. Most of the studies included had low risk of bias, and the certainty of evidence was mostly rated as moderate or high. Furthermore, we estimated the absolute differences for all comparisons and outcomes and presented a summary table by highlighting the certainty of evidence.^47^ Sensitivity analyses were conducted in fixed-effect models using bayesian methods.

This study also has several limitations. First, some comparison groups lacked direct evidence and could only be assessed through indirect comparisons. Second, among the comparison groups we assessed, some were rated as low certainty of evidence, mainly because of risk of bias and imprecision due to lack of evidence or a wide credible interval. Third, because of insufficient data, we could not perform subgroup analyses for a single comorbidity (bronchopulmonary dysplasia, congenital heart disease, or chronic lung disease) separately, geographic or race and ethnicity factors, and different RSV strains. In addition, we did not specifically focus on the effect of different drug doses. Fourth, the reduction in the rate of RSV-related hospitalizations, ICU admissions, and a few other outcomes may be associated with a reduction in the overall number of RSV infections; however, due to insufficient data, we were unable to perform analyses on the hospitalized population. Fifth, some factors, such as the thresholds for oxygen supplementation, the indication for hospitalization, or the difference in hospitalization rates caused by the large time span of the trials, may have affected the outcome; however, due to the lack of data in the included trials, these factors could not be considered in the data synthesis. Sixth, we did not take into account the economic cost of all the prophylaxes and patient preferences throughout the study. Future studies are needed to address these issues.

## Conclusions

Our systematic review and network meta-analysis comparing the efficacy and safety of 4 mAbs for preventing RSV infection in infants and children found associations with improvement of clinically important outcomes and no significant associations with RSV-related adverse effects and mortality. These findings suggest that motavizumab, nirsevimab, and palivizumab are associated with reduced rates of RSV infections and hospitalizations. Similar results were observed in the rate of supplemental oxygen use. Motavizumab was associated with significant differences in both the rate of MV use and ICU admissions; palivizumab was only associated with a reduction in ICU admissions.

## Notes

Supplement 1.
eMethods.

eResults.
**eTable 1.** Detailed Search Strategy **eTable 2.** Excluded Studies List **eTable 3.** Baseline Characteristics of Included Trials **eTable 4.** Baseline Characteristics of Included Trials **eTable 5.** Risk of Bias Assessment in Dichotomous Outcomes **eTable 6.** Risk of Bias Assessment in Continuous Outcomes **eTable 7.** League Table of All-Cause Mortality in Fixed-effect Model **eTable 8.** League Table of All-Cause Mortality in Random-effects Model **eTable 9.** League Table of Rate of RSV Infection in Fixed-effect Model **eTable 10.** League Table of Rate of RSV Infection in Random-effects Model **eTable 11.** League Table of RSV-Related Hospitalization in Fixed-effect Model **eTable 12.** League Table of RSV-Related Hospitalization in Random-effects Model **eTable 13.** League Table of Rate of ICU Admission in Fixed-effect Model **eTable 14.** League Table of Rate of ICU Admission in Random-effects Model **eTable 15.** League Table of Rate of Mechanical Ventilation Use in Fixed-effect Model **eTable 16.** League Table of Rate of Mechanical Ventilation Use in Random-effects Model **eTable 17.** League Table of Drug-related Adverse Events in Fixed-effect Model **eTable 18.** League Table of Drug-related Adverse Events in Random-effects Model **eTable 19.** League Table of Rate of Supplemental Oxygen Use in Fixed-effect Model **eTable 20.** League Table of Rate of Supplemental Oxygen Use in Random-effects Model **eTable 21.** Cumulative Ranking of Interventions for Different Outcomes **eTable 22.** Details of GRADE Assessment in All-cause Mortality **eTable 23.** Details of GRADE Assessment in RSV-related Hospitalization **eTable 24.** Details of GRADE Assessment in Rate of RSV Infection **eTable 25.** Details of GRADE Assessment in Drug-related Adverse Events **eTable 26.** Details of GRADE Assessment in Supplemental Oxygen Use **eTable 27.** Details of GRADE Assessment in Rate of Mechanical Ventilation Use **eTable 28.** Details of GRADE Assessment in Rate of ICU Admission **eTable 29.** Details of GRADE Assessment in Drug-related Serious Adverse Events **eTable 30.** Bayesian League Table of Rate of RSV Infection in Random-effects Model **eTable 31.** Bayesian League Table of Rate of RSV Infection in Fixed-effect Model **eTable 32.** Bayesian League Table of All-cause Mortality in Random-effects Model **eTable 33.** Bayesian League Table of All-cause Mortality in Fixed-effect Model **eTable 34.** Bayesian League Table of RSV-related Hospitalization in Random-effects Model **eTable 35.** Bayesian League Table of RSV-related Hospitalization in Fixed-effect Model **eTable 36.** Bayesian League Table of Rate of Supplemental Oxygen Use in Random-effects Model **eTable 37.** Bayesian League Table of Rate of Supplemental Oxygen Use in Fixed-effect Model **eTable 38.** Bayesian League Table of Rate of Mechanical Ventilation Use in Random-effects Model **eTable 39.** Bayesian League Table of Rate of Mechanical Ventilation Use in Fixed-effect Model **eTable 40.** Bayesian League Table of Rate of ICU Admission in Random-effects Model **eTable 41.** Bayesian League Table of Rate of ICU Admission in Fixed-effect Model **eTable 42.** Bayesian League Table of Drug-related Adverse Events in Random-effects Model **eTable 43.** Bayesian League Table of Drug-related Adverse Events in Fixed-effect Model **eTable 44.** Bayesian League Table of Drug-related Serious Adverse Events in Random-effects Model **eTable 45.** Bayesian League Table of Drug-related Serious Adverse Events in Fixed-effect Model **eTable 46.** Details of Continuous Outcomes **eFigure 1.** Risk-of-Bias Assessment **eFigure 2.** Network Plot of Rate of RSV Infection **eFigure 3.** Network Plot of All-cause Mortality **eFigure 4.** Network Plot of RSV-related Hospitalization **eFigure 5.** Network Plot of Supplemental Oxygen Use **eFigure 6.** Network Plot of Rate of Mechanical Ventilation Use **eFigure 7.** Network Plot of Rate of ICU Admission **eFigure 8.** Network Plot of Drug-related Adverse Events **eFigure 9.** Network Plot of Drug-related Serious Adverse Events **eFigure 10.** Results of Motavizumab Compared With Placebo in All-cause Mortality **eFigure 11.** Results of Nirsevimab Compared With Placebo in All-cause Mortality **eFigure 12.** Results of Palivizumab Compared With Placebo in All-cause Mortality **eFigure 13.** Results of Suptavumab Compared With Placebo in All-cause Mortality **eFigure 14.** Results of Motavizumab Compared With Palivizumab in All-cause Mortality **eFigure 15.** Results of Motavizumab in Combination With Palivizumab Compared With Motavizumab in All-cause Mortality **eFigure 16.** Results of Nirsevimab Compared With Placebo in Drug-related Adverse Events **eFigure 17.** Results of Palivizumab Compared With Placebo in Drug-related Adverse Events **eFigure 18.** Results of Suptavumab Compared With Placebo in Drug-related Adverse Events **eFigure 19.** Results of Motavizumab Compared With Palivizumab in Drug-related Adverse Events **eFigure 20.** Results of Motavizumab in Combination With Palivizumab Compared With Motavizumab in Drug-related Adverse Events **eFigure 21.** Results of Motavizumab Compared With Placebo in Drug-related Serious Adverse Events **eFigure 22.** Results of Nirsevimab Compared With Placebo in Drug-related Serious Adverse Events **eFigure 23.** Results of Palivizumab Compared With Placebo in Drug-related Serious Adverse Events **eFigure 24.** Results of Motavizumab Compared With Palivizumab in Drug-related Serious Adverse Events **eFigure 25.** Results of Motavizumab in Combination With Palivizumab Compared With Motavizumab in Drug-related Serious Adverse Events **eFigure 26.** Results of Nirsevimab Compared With Placebo in RSV-related Hospitalization **eFigure 27.** Results of Palivizumab Compared With Placebo in RSV-related Hospitalization **eFigure 28.** Results of Suptavumab Compared With Placebo in RSV-related Hospitalization **eFigure 29.** Results of Motavizumab Compared With Palivizumab in RSV-related Hospitalization **eFigure 30.** Results of Motavizumab Compared With Placebo in RSV Infection **eFigure 31.** Results of Nirsevimab Compared With Placebo in RSV Infection **eFigure 32.** Results of Palivizumab Compared With Placebo in RSV Infection **eFigure 33.** Results of Suptavumab Compared With Placebo in RSV Infection **eFigure 34.** Results of Motavizumab Compared With Placebo in Supplemental Oxygen Use **eFigure 35.** Results of Nirsevimab Compared With Placebo in Supplemental Oxygen Use **eFigure 36.** Results of Motavizumab Compared With Palivizumab in Supplemental Oxygen Use **eFigure 37.** Results of Motavizumab Compared With Placebo in Mechanical Ventilation Use **eFigure 38.** Results of Nirsevimab Compared With Placebo in Mechanical Ventilation Use **eFigure 39.** Results of Palivizumab Compared With Placebo in Mechanical Ventilation Use **eFigure 40.** Results of Motavizumab Compared With Palivizumab in Mechanical Ventilation Use **eFigure 41.** Results of Motavizumab Compared With Placebo in ICU Admission **eFigure 42.** Results of Nirsevimab Compared With Placebo in ICU Admission **eFigure 43.** Results of Palivizumab Compared With Placebo in ICU Admission **eFigure 44.** Results of Motavizumab Compared With Palivizumab in ICU Admission **eFigure 45.** Funnel Plot of All-cause Mortality **eFigure 46.** Node-split Plot of All-cause Mortality in Fixed-effect Model **eFigure 47.** Node-split Plot of All-cause Mortality in Random-effects Model **eFigure 48.** Node-split Plot of Drug-related Adverse Events in Fixed-effect Model **eFigure 49.** Node-split Plot of Drug-related Adverse Events in Random-effects Model **eFigure 50.** Node-split Plot of Rate of ICU Admission in Fixed-effect Model **eFigure 51.** Node-split Plot of Rate of ICU Admission in Random-effects Model **eFigure 52.** Node-split Plot of Rate of Mechanical Ventilation Use in Fixed-effect Model **eFigure 53.** Node-split Plot of Rate of Mechanical Ventilation Use in Random-effects Model **eFigure 54.** Node-split Plot of Rate of RSV Infection in Fixed-effect Model **eFigure 55.** Node-split Plot of Rate of RSV Infection in Random-effects Model **eFigure 56.** Node-split Plot of Rate of Supplemental Oxygen Use in Fixed-effect Model **eFigure 57.** Node-split Plot of Rate of Supplemental Oxygen Use in Random-effects Model **eFigure 58.** Node-split Plot of RSV-related Hospitalization in Fixed-effect Model **eFigure 59.** Node-split Plot of RSV-related Hospitalization in Random-effects Model **eFigure 60.** Contribute Plot of All-cause Mortality **eFigure 61.** Contribute Plot of Rate of RSV Infection **eFigure 62.** Contribute Plot of RSV-related Hospitalization **eFigure 63.** Contribute Plot of Rate of ICU Admission **eFigure 64.** Contribute Plot of Rate of Supplemental Oxygen Use **eFigure 65.** Contribute Plot of Rate of Mechanical Ventilation Use **eFigure 66.** Contribute Plot of Drug-related Adverse Events **eFigure 67.** Subgroup Analysis of Rate of RSV Infection **eFigure 68.** Subgroup Analysis of RSV-related Hospitalization **eFigure 69.** Meta-regression of Age in RSV-related Hospitalization **eFigure 70.** Meta-regression of Gender in RSV-related Hospitalization **eFigure 71.** Meta-regression of Publication Year in RSV-related Hospitalization **eFigure 72.** Comparison of Different Monoclonal Antibodies on Efficacy and Safety Outcomes

Supplement 2.
Data Sharing Statement
