# Supplementary material for: Monoclonal Antibody for the Prevention of Respiratory Syncytial Virus in Infants and Children: A Systematic Review and Network Meta-analysis
Source: JAMA Netw Open. 2023 Feb 17;6(2):e230023. doi: 10.1001/jamanetworkopen.2023.0023 (PMC9938429; doi:10.1001/jamanetworkopen.2023.0023)
Supplement: Supplement 1. — eMethods. eResults. eTable 1. Detailed Search Strategy eTable 2. Excluded Studies List eTable 3. Baseline Characteristics of Included Trials eTable 4. Baseline Characteristics of Included Trials eTable 5. Risk of Bias Assessment in Dichotomous Outcomes eTable 6. Risk of Bias Assessment in Continuous Outcomes eTable 7. League Table of All-Cause Mortality in Fixed-effect Model eTable 8. League Table of All-Cause Mortality in Random-effects Model eTable 9. League Table of Rate of RSV Infection in Fixed-effect Model eTable 10. League Table of Rate of RSV Infection in Random-effects Model eTable 11. League Table of RSV-Related Hospitalization in Fixed-effect Model eTable 12. League Table of RSV-Related Hospitalization in Random-effects Model eTable 13. League Table of Rate of ICU Admission in Fixed-effect Model eTable 14. League Table of Rate of ICU Admission in Random-effects Model eTable 15. League Table of Rate of Mechanical Ventilation Use in Fixed-effect Model eTable 16. League Table of Rate of Mechanical Ventilation Use in Random-effects Model eTable 17. League Table of Drug-related Adverse Events in Fixed-effect Model eTable 18. League Table of Drug-related Adverse Events in Random-effects Model eTable 19. League Table of Rate of Supplemental Oxygen Use in Fixed-effect Model eTable 20. League Table of Rate of Supplemental Oxygen Use in Random-effects Model eTable 21. Cumulative Ranking of Interventions for Different Outcomes eTable 22. Details of GRADE Assessment in All-cause Mortality eTable 23. Details of GRADE Assessment in RSV-related Hospitalization eTable 24. Details of GRADE Assessment in Rate of RSV Infection eTable 25. Details of GRADE Assessment in Drug-related Adverse Events eTable 26. Details of GRADE Assessment in Supplemental Oxygen Use eTable 27. Details of GRADE Assessment in Rate of Mechanical Ventilation Use eTable 28. Details of GRADE Assessment in Rate of ICU Admission eTable 29. Details of GRADE Assessment in Drug-related Serious Adverse Event [file jamanetwopen-e230023-s001.pdf]

Supplementary Online Content

Sun M, Lai H, Na F, et al. Monoclonal antibody for the prevention of respiratory syncytial virus in infants and children: a systematic review and network meta-analysis. *JAMA Netw Open*. 2023;6(2):e230023. doi:10.1001/jamanetworkopen.2023.0023

eMethods.
eResults.
eTable 1. Detailed Search Strategy
eTable 2. Excluded Studies List
eTable 3. Baseline Characteristics of Included Trials
eTable 4. Baseline Characteristics of Included Trials
eTable 5. Risk of Bias Assessment in Dichotomous Outcomes
eTable 6. Risk of Bias Assessment in Continuous Outcomes
eTable 7. League Table of All-Cause Mortality in Fixed-effect Model
eTable 8. League Table of All-Cause Mortality in Random-effects Model
eTable 9. League Table of Rate of RSV Infection in Fixed-effect Model
eTable 10. League Table of Rate of RSV Infection in Random-effects Model
eTable 11. League Table of RSV-Related Hospitalization in Fixed-effect Model
eTable 12. League Table of RSV-Related Hospitalization in Random-effects Model
eTable 13. League Table of Rate of ICU Admission in Fixed-effect Model
eTable 14. League Table of Rate of ICU Admission in Random-effects Model
eTable 15. League Table of Rate of Mechanical Ventilation Use in Fixed-effect Model
eTable 16. League Table of Rate of Mechanical Ventilation Use in Random-effects Model
eTable 17. League Table of Drug-related Adverse Events in Fixed-effect Model
eTable 18. League Table of Drug-related Adverse Events in Random-effects Model
eTable 19. League Table of Rate of Supplemental Oxygen Use in Fixed-effect Model
eTable 20. League Table of Rate of Supplemental Oxygen Use in Random-effects Model
eTable 21. Cumulative Ranking of Interventions for Different Outcomes
eTable 22. Details of GRADE Assessment in All-cause Mortality
eTable 23. Details of GRADE Assessment in RSV-related Hospitalization
eTable 24. Details of GRADE Assessment in Rate of RSV Infection
eTable 25. Details of GRADE Assessment in Drug-related Adverse Events
eTable 26. Details of GRADE Assessment in Supplemental Oxygen Use
eTable 27. Details of GRADE Assessment in Rate of Mechanical Ventilation Use
eTable 28. Details of GRADE Assessment in Rate of ICU Admission
eTable 29. Details of GRADE Assessment in Drug-related Serious Adverse Events
eTable 30. Bayesian League Table of Rate of RSV Infection in Random-effects Model
eTable 31. Bayesian League Table of Rate of RSV Infection in Fixed-effect Model
eTable 32. Bayesian League Table of All-cause Mortality in Random-effects Model
eTable 33. Bayesian League Table of All-cause Mortality in Fixed-effect Model
eTable 34. Bayesian League Table of RSV-related Hospitalization in Random-effects Model
eTable 35. Bayesian League Table of RSV-related Hospitalization in Fixed-effect Model
eTable 36. Bayesian League Table of Rate of Supplemental Oxygen Use in Random-effects Model
eTable 37. Bayesian League Table of Rate of Supplemental Oxygen Use in Fixed-effect Model
eTable 38. Bayesian League Table of Rate of Mechanical Ventilation Use in Random-effects Model
eTable 39. Bayesian League Table of Rate of Mechanical Ventilation Use in Fixed-effect Model

© 2023 Sun M et al. *JAMA Network Open*.

eTable 40. Bayesian League Table of Rate of ICU Admission in Random-effects Model
eTable 41. Bayesian League Table of Rate of ICU Admission in Fixed-effect Model
eTable 42. Bayesian League Table of Drug-related Adverse Events in Random-effects Model
eTable 43. Bayesian League Table of Drug-related Adverse Events in Fixed-effect Model
eTable 44. Bayesian League Table of Drug-related Serious Adverse Events in Random-effects Model
eTable 45. Bayesian League Table of Drug-related Serious Adverse Events in Fixed-effect Model
eTable 46. Details of Continuous Outcomes
eFigure 1. Risk of Bias Assessment
eFigure 2. Network Plot of Rate of RSV Infection
eFigure 3. Network Plot of All-cause Mortality
eFigure 4. Network Plot of RSV-related Hospitalization
eFigure 5. Network Plot of Supplemental Oxygen Use
eFigure 6. Network Plot of Rate of Mechanical Ventilation Use
eFigure 7. Network Plot of Rate of ICU Admission
eFigure 8. Network Plot of Drug-related Adverse Events
eFigure 9. Network Plot of Drug-related Serious Adverse Events
eFigure 10. Results of Motavizumab Compared With Placebo in All-cause Mortality
eFigure 11. Results of Nirsevimab Compared With Placebo in All-cause Mortality
eFigure 12. Results of Palivizumab Compared With Placebo in All-cause Mortality
eFigure 13. Results of Suptavumab Compared With Placebo in All-cause Mortality
eFigure 14. Results of Motavizumab Compared With Palivizumab in All-cause Mortality
eFigure 15. Results of Motavizumab in Combination With Palivizumab Compared With Motavizumab in All-cause Mortality
eFigure 16. Results of Nirsevimab Compared With Placebo in Drug-related Adverse Events
eFigure 17. Results of Palivizumab Compared With Placebo in Drug-related Adverse Events
eFigure 18. Results of Suptavumab Compared With Placebo in Drug-related Adverse Events
eFigure 19. Results of Motavizumab Compared With Palivizumab in Drug-related Adverse Events
eFigure 20. Results of Motavizumab in Combination With Palivizumab Compared With Motavizumab in Drug-related Adverse Events
eFigure 21. Results of Motavizumab Compared With Placebo in Drug-related Serious Adverse Events
eFigure 22. Results of Nirsevimab Compared With Placebo in Drug-related Serious Adverse Events
eFigure 23. Results of Palivizumab Compared With Placebo in Drug-related Serious Adverse Events
eFigure 24. Results of Motavizumab Compared With Palivizumab in Drug-related Serious Adverse Events
eFigure 25. Results of Motavizumab in Combination With Palivizumab Compared With Motavizumab in Drug-related Serious Adverse Events
eFigure 26. Results of Nirsevimab Compared With Placebo in RSV-related Hospitalization
eFigure 27. Results of Palivizumab Compared With Placebo in RSV-related Hospitalization
efigure 28. Results of Suptavumab Compared With Placebo in RSV-related Hospitalization
eFigure 29. Results of Motavizumab Compared With Palivizumab in RSV-related Hospitalization
eFigure 30. Results of Motavizumab Compared With Placebo in RSV Infection
eFigure 31. Results of Nirsevimab Compared With Placebo in RSV Infection
eFigure 32. Results of Palivizumab Compared With Placebo in RSV Infection
eFigure 33. Results of Suptavumab Compared With Placebo in RSV Infection
eFigure 34. Results of Motavizumab Compared With Placebo in Supplemental Oxygen Use
eFigure 35. Results of Nirsevimab Compared With Placebo in Supplemental Oxygen Use

© 2023 Sun M et al. *JAMA Network Open*.

eFigure 36. Results of Motavizumab Compared With Palivizumab in Supplemental Oxygen Use
eFigure 37. Results of Motavizumab Compared With Placebo in Mechanical Ventilation Use
eFigure 38. Results of Nirsevimab Compared With Placebo in Mechanical Ventilation Use
eFigure 39. Results of Palivizumab Compared With Placebo in Mechanical Ventilation Use
eFigure 40. Results of Motavizumab Compared With Palivizumab in Mechanical Ventilation Use
eFigure 41. Results of Motavizumab Compared With Placebo in ICU Admission
eFigure 42. Results of Nirsevimab Compared With Placebo in ICU Admission
eFigure 43. Results of Palivizumab Compared With Placebo in ICU Admission
eFigure 44. Results of Motavizumab Compared With Palivizumab in ICU Admission
eFigure 45. Funnel Plot of All-cause Mortality
eFigure 46. Node-split Plot of All-cause Mortality in Fixed-effect Model
eFigure 47. Node-split Plot of All-cause Mortality in Random-effects Model
eFigure 48. Node-split Plot of Drug-related Adverse Events in Fixed-effect Model
eFigure 49. Node-split Plot of Drug-related Adverse Events in Random-effects Model
eFigure 50. Node-split Plot of Rate of ICU Admission in Fixed-effect Model
eFigure 51. Node-split Plot of Rate of ICU Admission in Random-effects Model
eFigure 52. Node-split Plot of Rate of Mechanical Ventilation Use in Fixed-effect Model
eFigure 53. Node-split Plot of Rate of Mechanical Ventilation Use in Random-effects Model
eFigure 54. Node-split Plot of Rate of RSV Infection in Fixed-effect Model
eFigure 55. Node-split Plot of Rate of RSV Infection in Random-effects Model
eFigure 56. Node-split Plot of Rate of Supplemental Oxygen Use in Fixed-effect Model
eFigure 57. Node-split Plot of Rate of Supplemental Oxygen Use in Random-effects Model
eFigure 58. Node-split Plot of RSV-related Hospitalization in Fixed-effect Model
eFigure 59. Node-split Plot of RSV-related Hospitalization in Random-effects Model
eFigure 60. Contribute Plot of All-cause Mortality
eFigure 61. Contribute Plot of Rate of RSV Infection
eFigure 62. Contribute Plot of RSV-related Hospitalization
eFigure 63. Contribute Plot of Rate of ICU Admission
eFigure 64. Contribute Plot of Rate of Supplemental Oxygen Use
eFigure 65. Contribute Plot of Rate of Mechanical Ventilation Use
eFigure 66. Contribute Plot of Drug-related Adverse Events
eFigure 67. Subgroup Analysis of Rate of RSV Infection
eFigure 68. Subgroup Analysis of RSV-related Hospitalization
eFigure 69. Meta-regression of Age in RSV-related Hospitalization
eFigure 70. Meta-regression of Gender in RSV-related Hospitalization
eFigure 71. Meta-regression of Publication Year in RSV-related Hospitalization
eFigure 72. Comparison of Different Monoclonal Antibodies on Efficacy and Safety Outcomes

This supplementary material has been provided by the authors to give readers additional information about their work.

© 2023 Sun M et al. *JAMA Network Open*.

eMethods

Statistical Analysis

Network meta-analysis: We conducted a random-effect model network meta-analysis using a consistency model with “netmeta V1.0-1” package under a frequentist framework in R 1.3.1093 software. Frequentist results were shown in league tables. A league table is a square matrix showing all pairwise comparisons in a network meta-analysis. Typically, both treatment estimates and confidence intervals are shown. The netsplit function was used to assess the inconsistency between direct and indirect estimates and obtain indirect effect estimates. Network estimates that have indirect and direct evidence, these estimates are split into three components. The network estimate, indirect estimate and direct estimate are inspected for consistency. Consistency is assessed mainly by inspection of the point estimate and the confidence intervals. P-scores were used to rank each intervention with the interpretation of the mean extent of certainty that one intervention was better than the other. We used contribute plots to presents the contribution of every interventions. This fuction generates the contribution of direct comparisons to every network treatment comparison. The contribution matrix shows how much each direct treatment effect contributes to each treatment effect estimate from network meta-analysis. The output is a matrix where rows represent network treatment effects and columns represent the contribution of direct treatment effects.

Pairwise meta-analyses: Pairwise meta-analyses were performed using RevMan 5.4.1, and the data were synthesized with a random-effects model to obtain direct effect estimates for each pairwise comparison.

Subgroup analysis, meta-regression, and sensitivity analysis: We conducted the subgroup analysis in direct comparisons for a priori specified variables including health status (healthy vs unhealthy, a predefined hypothesis of larger effect in unhealthy population; unhealthy was defined as participants reported having high-risk factors at baseline). We also performed meta-regression in gestational age, and ratio of male and female. Meta-regression is similar to simple regression, where the outcome of interest is predicted on the basis of one or more explanatory variables. Besides, we performed sensitivity analyses to observe the robustness of results by repeating the analyses using a bayesian hierarchical model, and using both fixed-effects model and random-effects model.

© 2023 Sun M et al. *JAMA Network Open*.

eResults

Network meta-analysis: Frequentist results were shown in league tables (appendix table S7-20). P-scores were used to rank each intervention with the interpretation of the mean extent of certainty that one intervention was better than the other (appendix table S21). The network plots were shown in appendix figure S2-9. The line width is proportional to the number of studies comparing each pair of interventions, and the size of each node is proportional to the number of participants (sample size). The node-split plots were shown in appendix figure S48-61. The contribute plots were shown in appendix figure S62-68, the matrix showed how much each direct treatment effect contributes to each treatment effect estimate from network meta-analysis.
Pairwise meta-analyses: Results of pairwise meta-analyses were shown in forest plots (appendix figure S10-46). Funnel plot of all-cause mortality was presented in appendix figure S47, showing that there may be no publication bias.
Subgroup analysis, meta-regression, and sensitivity analysis: Bayesian results were shown in league tables as sensitivity analyses (appendix table S31-46).

© 2023 Sun M et al. *JAMA Network Open*.

Appendix table S1: detailed search strategy

Database: PubMed <Mar 13, 2022>

Search Strategy:

#	Searches	Results
1	"Palivizumab" [Mesh]	825
2	((MEDI 493[Title/Abstract]) OR (MEDI-493[Title/Abstract]) OR (MEDI493[Title/Abstract]) OR (Monoclonal Antibody MEDI-493[Title/Abstract]) OR (Monoclonal Antibody MEDI 493[Title/Abstract]) OR (Monoclonal Antibody MEDI493[Title/Abstract]))	9
3	"Synagis" [Title/Abstract]	107
4	or/1-3	864
5	Motavizumab[Title/Abstract]	51
6	Nirsevimab[Title/Abstract]	14
7	MEDI8897[Title/Abstract]	7
8	Monoclonal antibod*[Title/Abstract]	197,940
9	Antibody, Monoclonal[Title/Abstract]	112
10	"Antibodies, Monoclonal"[Mesh]	261,297
11	or/4-10	345,203
12	"Respiratory Syncytial Viruses"[Mesh]	9,813
13	((Respiratory Syncytial Virus[Title/Abstract]) OR (Syncytial Virus, Respiratory[Title/Abstract]) OR (Syncytial Viruses, Respiratory[Title/Abstract]) OR (Virus, Respiratory Syncytial[Title/Abstract]) OR (Viruses, Respiratory Syncytia[Title/Abstract]) OR (Respiratory Syncytial Virus, Human) OR (Human respiratory syncytial virus))	15,694
14	#12 OR #13	16,669
15	"Respiratory Tract Infections"[Mesh]	518,149
16	((Infection, Respiratory Tract[Title/Abstract]) OR (Respiratory Tract Infection[Title/Abstract]) OR (Infections, Respiratory[Title/Abstract]) OR (Infections, Respiratory Tract[Title/Abstract]) OR (Respiratory Infections[Title/Abstract]))	28,593
17	#15 OR #16	53,1434
18	"Pneumonia"[Mesh]	242,236
19	"Bronchiolitis"[Mesh]	9,276
20	#14 OR #17 OR #18 OR #19	543,979
21	#11 and #20	7,413
22	“clinical trial”[Publication Type]	931,808
23	“Clinical Trials as Topic”[Mesh]	371,437
24	“Double-Blind Method”[Mesh]	170,548
25	(randomized[TIAB] AND (trial[TIAB] OR trials[tiab]))	411,238

© 2023 Sun M et al. *JAMA Network Open*.

26	((single[TIAB] OR double[TIAB] OR doubled[TIAB] OR triple[TIAB] OR tripled[TIAB] OR treble[TIAB] OR treble[TIAB]) AND (blind*[TIAB] OR mask*[TIAB]))	210,876
27	or/22-26	1,408,659
28	#21 and #27	890

Database: Embase <Mar 13, 2022>

Search Strategy:

#	Searches	Results
1	'palivizumab'/exp	3,293
2	palivizumab:ti,ab,kw	1,544
3	synagis:ti,ab,kw	179
4	'medi493':ti,ab,kw	12
5	'motavizumab'/exp	304
6	motavizumab:ti,ab,kw	69
7	'nirsevimab'/exp	71
8	nirsevimab:ti,ab,kw	20
9	MEDI8897:ti,ab,kw	15
10	'monoclonal antibody'/exp	673,595
11	or/1-10	673,737
12	'respiratory syncytial virus infection'/exp	6,669
13	'respiratory syncytial virus' OR rsv	32,136
14	'bronchiolitis'/exp	24,500
15	bronchiolit*:ti,ab,kw	19,154
16	'pneumonia'/exp	369,534
17	pneumon*:ti,ab,kw OR bronchopneumon*:ti,ab,kw OR pleuropneumon*:ti,ab,kw	320,840
18	'respiratory tract infection'/exp	517,513
19	'lower respiratory infection*':ti,ab,kw	2,305
20	'lower respiratory tract infection*':ti,ab,kw OR lrti:ti,ab,kw	11,138
21	'pneumovirus'/exp OR 'human respiratory syncytial virus'/exp	20,047
22	or/12-21	884,506
23	#11 and #22	44,911
24	rct:ti,ab,kw OR 'randomized controlled trial':ti,ab,kw	163,460
25	'randomized controlled trial'/exp	702,816
26	#24 or #25	756,536
27	#23 and 26	3,662

Database: CENTRAL <Mar 13, 2022>

Search Strategy:

#	Searches	Results
1	MeSH descriptor: [Palivizumab] explode all trees	49
2	(palivizumab):ti,ab,kw	146
3	(synagis):ti,ab,kw	33
4	(medi493):ti,ab,kw	0
5	(motavizumab):ti,ab,kw	23

© 2023 Sun M et al. *JAMA Network Open*.

6	(nirsevimab):ti,ab,kw	6
7	(MEDI8897):ti,ab,kw	14
8	MeSH descriptor: [Antibodies, Monoclonal] explode all trees	15,035
9	or/1-8	15,147
10	MeSH descriptor: [Respiratory Syncytial Virus] explode all trees	177
11	MeSH descriptor: [Respiratory Syncytial Virus Infections] explode all trees	330
12	((respiratory syncytial virus* or rsv)):ti,ab,kw	1290
13	MeSH descriptor: [Respiratory Syncytial Virus, Human] explode all trees	69
14	MeSH descriptor: [Bronchiolitis] in all MeSH products	398
15	(bronchiolit*):ti,ab,kw	1,536
16	((pneumon* or bronchopneumon* or pleuropneumon*)):ti,ab,kw	21,846
17	MeSH descriptor: [Respiratory Tract Infections] explode all trees	16,984
18	((lower respiratory infection*)):ti,ab,kw	4,409
19	((lower respiratory tract infection* or lrti)):ti,ab,kw	3,420
20	or/10-19	38,085
21	#9 and #20	765

Database: ClinicalTrials.gov <Mar 13, 2022>

**#**	**Searches**	**Results**
1	Intervention: palivizumab OR synagis OR med493 OR motavizumab OR nirsevimab OR MEDI8897	43

© 2023 Sun M et al. *JAMA Network Open*.

Appendix table S2: Excluded studies list

Author, Year	Population	Intervention	Outcome	Study design	Exclude reasons
Ramilo O, 2014	healthy infants	motavizumab	Clinical outcomes data collected included the duration of hospitalization, the need for supplemental oxygen and mechanical ventilation and admission to and length of stay in the intensive care unit (ICU). The incidence of ≥1 or ≥3 medically attended wheezing episodes	RCT	ineligible population
Meissner HC, 1999	preterm infants with or without bronchopulmonary dysplasia (BPD)	SB 209763	laboratory tests, Safety parameters, RSV disease symptoms, Pharmacokinetic parameters	RCT	unable to extract data
Malley R, 1998	RSV infection	palivizumab	serum concentration	RCT	ineligible population
Mori M, 2014	children ≤24 months	palivizumab	rate of RSV-associated hospitalizations occurring, Blood samples, Adverse events	NRCT	wrong study designs
SÁEZ-LLORENS X, 1998	preterm infants with or without bronchopulmonary dysplasia (BPD)	palivizumab	Adverse events, laboratory tests, Pharmacokinetic and immunogenicity assessments	NRCT	wrong study designs
Groothuis JR, 2003	preterm infants	palivizumab	need for hospitalization and hospital course, safety	NRCT	wrong study designs
Brunv, 2000	preterm infants	palivizumab	——	NRCT	wrong study designs
Alansari K, 2019	Infants with a confirmed diagnosis of RSV bronchiolitis	palivizumab	The primary efficacy outcome was readmission to either the infirmary- observation unit, hospital, or PICU during the 3 weeks after discharge.Final secondary outcomes were time to medical readiness for discharge on the initial admission, revisit (but not requiring admission) to any medical facility for the same illness in the 3- week follow-up period, and need for transfer to the PICU during the initial admission	RCT	ineligible population
Alyamovskaya, 2012	50–74 years old participants	long-acting beta-2-agonists	The forced expiratory volume in 1 s (FEV1), Skin blood perfusion changes	RCT	ineligible population
Lagos R, 2009	healthy children aged <2 years (gestational age >36 weeks)	motavizumab	lower respiratory disease scores, the durations of hospitalization, supplemental oxygen use during hospitalization, in- tensive care unit stay, and mechanical ventilation were also recorded. Adverse events (AEs) and serious AEs were monitored through study day 30	RCT	ineligible population
Null D, 2005	Children at 6 selected centers who had participated in IMpact-	palivizumab	Adverse events, palivizumab concentrations and anti-palivizumab reactivity	NRCT	wrong study designs

© 2023 Sun M et al. *JAMA Network Open*.

	RSV during the 1996–1997 winter season				
Sa´ez-Llorens X, 2004	children ≤24 months	palivizumab	RSV hospitalization and for 30 days after study drug administration for safety, determination of drug serum concentration and clinical outcome	RCT	ineligible population
Domachowske J, 2022	preterm infants with heart or lung disease	nirsevimab	Data on adverse events that occurred during treatment and adverse events of special interest were collected through day 361. Pharma cokinetics and antidrug antibody levels were assessed (see Section S3)	NRCT	wrong study designs
Marchac, 2006	——	palivizumab	——	NRCT	wrong study designs
Man WH, 2020	healthy preterm infants; Infants were younger than 6 months of age at the start of the RSV season	palivizumab	primary outcome was to assess the effect of palivizumab during infancy on the respiratory microbiota composition at age 1 year and 6 years; secondary outcomes were assessment of the effect of proven RSV infection in the first year of life on the respiratory microbiota composition at age 1 years and 6 years	RCT	unable to extract data
Makari D, 2014	preterm infants with chronic lung disease	palivizumab liquid	adverse events, Antidrug Antibodies	RCT	ineligible controls
Blanken M, 2012	Healthy preterm infants	palivizumab	The primary endpoint of the study was the number of wheezing days during the first year of life	RCT	unable to extract data

© 2023 Sun M et al. *JAMA Network Open*.

Appendix table S3: Baseline characteristics of included trials

First author, Year	Register number	Journal	Country	Weight (mean±SD)	Asian (%)	White (%)	Black (%)	Hispanic (%)	American Indian or Alaska Native (%)	Native Hawaiian or other Pacific Islander (%)	Other (%)	Multiple categories (%)
**Griffin, 2020**	NCT02878330	N Engl J Med	Argentina, Australia, Belgium, Brazil, Bulgaria, Canada, Chile, Czech Republic, Estonia, Finland, France, Hungary, Italy, Latvia, Lithuania, New Zealand, Poland, South Africa, Spain, Sweden, Turkey, United Kingdom, United States	4.57±1.93	1.03	72.18	17.6	NR	0.07	0.76	7.16	1.17
**O'Brien, 2015**	NCT00121108	Lancet Infect Dis	United States	5.16±1.79	NA	NA	NA	NR	98.78	NA	1.22	NA
**Subramanian, 1998**	NR	Pediatric Infectious Disease Journal	NR	4.67±0.72	NR	NR	NR	NR	NR	NR	NR	NR
**Hammitt, 2022**	NCT03979313	N Engl J Med	Northern (69%), Southern (31%): Austria, Belgium, Bulgaria, Canada, Estonia, Finland, France, Germany, Israel, Japan, Lativa, Lithuania, Poland, Republic of Korea, Russia, South Africa, Spain, Sweden, United Kingdom,	NR	3.6	53.5	28.4	NR	5.6	0.7	8.1	

**Feltes, 2011**	NCT00240890, NCT00538785	Pediatric Research	Austria, Belgium, Bulgaria, Canada, Czech Republic, Denmark, France, Germany, Hungary, Israel, Lebanon, Poland, Russia, Spain, Sweden, United Kingdom, United States	6.72±2.61	1.46	86.56	3.48	3.56	NR	NR	4.94	NR
**Carbonell-Estrany, 2010**	NCT00129766	Pediatrics	United States, Canada, Austria, Czech Republic, Denmark, France, Germany, Greece, Hungary, Italy, Poland, Spain, Sweden, United Kingdom, Argentina, Australia, Brazil, Bulgaria, Chile, Iceland, Israel, New Zealand, Russia, Turkey	4.45±1.99	1.28	78.57	7.85	8.58	NR	NR	3.69	NR
**Feltes, 2003**	NR	Journal of Pediatrics	United States, Canada, Poland, the United Kingdom, Germany, Sweden, France	6.05±0.1	NR	70.86	8.78	11.11	NR	NR	9.25	NR
**IMpact-RSV, 1998**	NR	Pediatrics	United States, the United Kingdom, Canada	4.83±0.1	2.2	58.06	23.7	10.92	NR	NR	5.13	NR
**Simões, 2021**	NCT02325791	Clin Infect Dis Clin Infect Dis.	18 countries	4.47±1.54	0.7	86.86	8.79	NR	0.17	0.17	2.35	NR
**Scheltema, 2018**	ISRCTN73641710	Lancet Respir Med	Netherlands	2.29	NR	NR	NR	NR	NR	NR	NR	NR
**Domachowske, 2018**	NCT02290340	Pediatric Infectious Disease Journal	United States, South Africa, Chile	6.82±1.9	1.4	11.3	57.7	NR	1.4	NR	25.4	2.8
**Tavsu, 2014**	NR	Am J Perinatol.	NR	1.36±0.26	NR	NR	NR	NR	NR	NR	NR	NR

© 2023 Sun M et al. *JAMA Network Open*

**Fernández, 2010**	NCT00316264	BMC Pediatrics	Chile, New Zealand, Australia	4.6±1.8	NR	27.3	NR	63.8	NR	NR	8.8	NR

© 2023 Sun M et al. *JAMA Network Open*

Appendix table S4: Baseline characteristics of included trials

First author, Year	≥1 smoker in household	Overall smoke exposure	Household crowding, >5 individuals	Bronchopulmonary dysplasia (BPD) (%)	Congenital heart disease (CHD) (%)	Chronic lung disease (CLD) (%)	Timpoint of trial (before/during/afte r RSV season)	Any atopy (%)	Asthma/ Wheezing (%)	Eczema (%)	Hay fever (%)	Funding
**Griffin, 2020**	NR	NR	NR	NA	NA	NA	before	NR	NR	NR	NR	MedImmune/AstraZeneca and Sanofi Pasteur
**O'Brien, 2015**	22.57	67.94	77.76	NA	NA	NA	during	20.73	18.85	4.33	8.6	MedImmune
**Subramanian, 1998**	NR	NR	NR	83.87	NR	NR	during	NR	NR	NR	NR	NR
**Hammitt, 2022**	NR	NR	NR	NA	NA	NA	before	NR	NR	NR	NR	MedImmune/ AstraZeneca and Sanofi
**Blanken, 2013**	16.08/27.74/ 38.93	NR	NR	NA	NA	NA	during	36.6/35 .66	10.72/11.19	18.18/13. 05	21.68/2 2.38	Abbott Laboratories and by the Netherlands Organization for Health Research and Development
**Feltes, 2011**	38.14	NR	NR	NA	100	NA	during	38.38	NR	NR	NR	MedImmune
**Carbonell-Estrany, 2010**	32.99	NR	NR	NR	NR	21.78	during	44.6	27.99	16.46	20.38	NR
**Feltes, 2003**	33.72	NR	NR	NA	100	NA	during	NR	28.21	NR	NR	NR

© 2023 Sun M et al. *JAMA Network Open*

**IMpact-RSV, 1998**	35.15	NR	NR	50.73	NA	NA	during	NR	35.82	16.44	28.96	NR
**Simões, 2021**	NR	NR	NR	NA	NA	NA	over 3 RSV seasons	NR	NR	NR	NR	Regeneron Pharmaceuticals
**Scheltema, 2018**	16.08/27.74/38.93	NR	NR	NA	NA	NA	during	36.6/35.66	10.72/11.19	18.18/13.05	21.68/22.38	AbbVie
**Domachowske, 2018**	NR	NR	NR	NA	NA	NA	NR	NR	NR	NR	NR	MedImmune
**Tavsu, 2014**	NR	NR	NR	NA	NA	NA	during	NR	NR	NR	NR	NR
**Fernández, 2010**	NR	NR	NR	NR	NR	15.8	during	NR	NR	NR	NR	MedImmune

© 2023 Sun M et al. *JAMA Network Open*

Appendix table S5: risk of bias assessment in dichotomous outcomes

Study	Randomization process generated	Deviations from the intended intervention	Missing data	Measurement of the outcome	Selection of the reported results	Overall
Griffin MP, 2020	Low	Low	Low	Low	Low	Low
O'Brien KL, 2015	Low	Low	Low	Low	Low	Low
Subramanian KN, 1998	Low	Low	Low	Low	Low	Low
Hammitt LL, 2022	Low	Low	Low	Low	Low	Low
Blanken MO, 2013	Low	Low	Low	Low	Low	Low
FELTES TF, 2011	Low	Low	Low	Low	Low	Low
Carbonell-Estrany X, 2010	Low	Low	Low	Low	Low	Low
FELTES TF, 2003	Low	Low	Low	Low	Low	Low
IMpact-RSV, 1998	Low	Low	Low	Low	Low	Low
Simões EAF, 2021	Someconcerns	Low	Low	Low	Low	Someconcerns
Scheltema NM, 2018	Low	Low	Low	Low	Low	Low
Domachowske JB, 2018	Someconcerns	Low	Low	Low	Low	Someconcerns
Tavsu I, 2014	Someconcerns	Someconcerns	Low	Low	Low	Someconcerns
Fernández P, 2010	Low	Low	Low	Low	Low	Someconcerns*

* MedImmune was involved in study design, and analysis and interpretation of data.

© 2023 Sun M et al. *JAMA Network Open*.

Appendix table S6: risk of bias assessment in continuous outcomes

Study	Randomization process generated	Deviations from the intended intervention	Missing data	Measurement of the outcome	Selection of the reported results	Overall
Griffin MP, 2020	Low	Low	Low	Low	Low	Low
O'Brien KL, 2015	Low	Low	Low	Low	Someconcerns	Someconcerns
Subramanian KN, 1998	Low	Low	Low	Low	Low	Low
Hammitt LL, 2022	Low	Low	Low	Low	Low	Low
Blanken MO, 2013	Low	Low	Low	Low	Low	Low
FELTES TF, 2011	Low	Low	Low	Low	Low	Low
Carbonell-Estrany X, 2010	Low	Low	Low	Low	Someconcerns	Someconcerns
FELTES TF, 2003	Low	Low	Low	Low	Someconcerns	Someconcerns
IMpact-RSV, 1998	Low	Low	Low	Low	Low	Low
Simões EAF, 2021	Someconcerns	Low	Low	Low	Low	Someconcerns
Scheltema NM, 2018	Low	Low	Low	Low	Low	Low
Domachowske JB, 2018	Someconcerns	Low	Low	Low	Low	Someconcerns
Tavsu I, 2014	Someconcerns	Someconcerns	Low	Low	Low	Someconcerns
Fernández P, 2010	Low	Low	Low	Low	Low	Someconcerns*

* MedImmune was involved in study design, and analysis and interpretation of data.

© 2023 Sun M et al. *JAMA Network Open*.

Appendix table S7-S20: league tables of outcomes in fixed and random models

Outcomes were reported as odds ratio (95% credible interval). All tables list the treatments in alphabetical order. The estimate is in the cell in common between the column-defining intervention and the row-defining intervention. The league table contains the network estimates in the lower triangle and the direct treatment estimates from pairwise comparisons in the upper triangle. The blanks indicate that there is no comparison group for this outcome.

*Included studies for each outcome assessment:

Author, year	Intervention vs comparison	all-cause mortality	rate of RSV infection	RSV-related hospitalization	rate of ICU admission	rate of mechanical ventilation use	drug-related adverse events	rate of supplemental oxygen use
Griffin, 2020	Nirsevimab vs placebo	√	√	√	√	√	√	√
O'Brien, 2015	Motavizumab vs placebo	√	√		√	√		√
Subramanian, 1998	Palivizumab vs placebo	√	√	√			√	
Hammitt, 2022	Nirsevimab vs placebo	√	√	√			√	
Blanken, 2013	Palivizumab vs placebo		√	√				
Feltes, 2011	Motavizumab vs Palivizumab	√					√	
Carbonell-Estrany, 2010	Motavizumab vs Palivizumab			√	√	√	√	√
Feltes, 2003	Palivizumab vs placebo	√		√	√	√	√	
IMpact-RSV, 1998	Palivizumab vs placebo	√		√				
Simões, 2021	Suptavumab vs placebo	√	√	√			√	
Scheltema, 2018	Palivizumab vs placebo		√					
Domachowske, 2018	Nirsevimab vs placebo	√					√	
Tavsu, 2014	Palivizumab vs placebo		√	√				
Fernández, 2010	M+P vs Motavizumab	√					√	

© 2023 Sun M et al. *JAMA Network Open*.

Appendix table S7: league table of all-cause mortality in fixed effects model

M+P	1.12<br>(0.10 - 12.54)	.	.	.
1.12<br>(0.10 - 12.54)	Motavizumab	.	0.89<br>(0.36 - 2.20)	1.00<br>(0.18 - 5.48)
2.43<br>(0.11 - 55.88)	2.16<br>(0.29 - 16.03)	Nirsevimab	.	0.33<br>(0.05 - 1.97)
1.10<br>(0.09 - 14.04)	0.98<br>(0.44 - 2.20)	0.45<br>(0.07 - 2.93)	Palivizumab	0.70<br>(0.41 - 1.19)
0.80<br>(0.06 - 10.46)	0.71<br>(0.29 - 1.74)	0.33<br>(0.05 - 1.97)	0.72<br>(0.43 - 1.21)	Placebo

© 2023 Sun M et al. *JAMA Network Open*.

Appendix table S8: league table of all-cause mortality in random effects model

M+P	1.12 (0.10 - 12.54)	.	.	.
1.12 (0.10 - 12.54)	Motavizumab	.	0.89 (0.36 - 2.20)	1.00 (0.18 - 5.48)
2.43 (0.11 - 55.88)	2.16 (0.29 - 16.03)	Nirsevimab	.	0.33 (0.05 - 1.97)
1.10 (0.09 - 14.04)	0.98 (0.44 - 2.20)	0.45 (0.07 - 2.93)	Palivizumab	0.70 (0.41 - 1.19)
0.80 (0.06 - 10.46)	0.71 (0.29 - 1.74)	0.33 (0.05 - 1.97)	0.72 (0.43 - 1.21)	Placebo

© 2023 Sun M et al. *JAMA Network Open*.

Appendix table S9: league table of rate of RSV infection in fixed effects model

Motavizumab	.	.	**0.17 (0.12 - 0.23)**	.
0.68 (0.41 - 1.14)	Nirsevimab	.	**0.24 (0.16 - 0.37)**	.
**0.52 (0.32 - 0.86)**	0.77 (0.44 - 1.35)	Palivizumab	**0.32 (0.22 - 0.47)**	.
**0.17 (0.12 - 0.23)**	**0.24 (0.16 - 0.37)**	**0.32 (0.22 - 0.47)**	Placebo	0.94 (0.65 - 1.36)
**0.16 (0.10 - 0.25)**	**0.23 (0.13 - 0.40)**	**0.30 (0.18 - 0.51)**	0.94 (0.65 - 1.36)	Suptavumab

© 2023 Sun M et al. *JAMA Network Open*.

Appendix table S10: league table of rate of RSV infection in random effects model

Motavizumab	.	.	**0.17 (0.12 - 0.23)**	.
0.68 (0.41 - 1.14)	Nirsevimab	.	**0.24 (0.16 - 0.37)**	.
**0.52 (0.32 - 0.86)**	0.77 (0.44 - 1.35)	Palivizumab	**0.32 (0.22 - 0.47)**	.
**0.17 (0.12 - 0.23)**	**0.24 (0.16 - 0.37)**	**0.32 (0.22 - 0.47)**	Placebo	0.94 (0.65 - 1.36)
**0.16 (0.10 - 0.25)**	**0.23 (0.13 - 0.40)**	**0.30 (0.18 - 0.51)**	0.94 (0.65 - 1.36)	Suptavumab

© 2023 Sun M et al. *JAMA Network Open*.

Appendix table S11: league table of RSV-related hospitalization in fixed effects model

Motavizumab	.	0.73 (0.50 - 1.08)	.	.
1.34 (0.60 - 3.01)	Nirsevimab	.	**0.25 (0.13 - 0.47)**	.
0.73 (0.50 - 1.08)	0.55 (0.27 - 1.12)	Palivizumab	**0.45 (0.34 - 0.60)**	.
**0.33 (0.20 - 0.53)**	**0.25 (0.13 - 0.47)**	**0.45 (0.34 - 0.60)**	Placebo	1.00 (0.52 - 1.92)
**0.33 (0.15 - 0.74)**	**0.25 (0.10 - 0.62)**	**0.45 (0.22 - 0.92)**	1.00 (0.52 - 1.92)	Suptavumab

© 2023 Sun M et al. *JAMA Network Open*.

Appendix table S12: league table of RSV-related hospitalization in random effects model

Motavizumab	.	0.73 (0.50 - 1.08)	.	.
1.34 (0.60 - 3.01)	Nirsevimab	.	**0.25 (0.13 - 0.47)**	.
0.73 (0.50 - 1.08)	0.55 (0.27 - 1.12)	Palivizumab	**0.45 (0.34 - 0.60)**	.
**0.33 (0.20 - 0.53)**	**0.25 (0.13 - 0.47)**	**0.45 (0.34 - 0.60)**	Placebo	1.00 (0.52 - 1.92)
**0.33 (0.15 - 0.74)**	**0.25 (0.10 - 0.62)**	**0.45 (0.22 - 0.92)**	1.00 (0.52 - 1.92)	Suptavumab

© 2023 Sun M et al. *JAMA Network Open*.

Appendix table S13: league table of rate of ICU admission in fixed effects model

Motavizumab	0.52<br>(0.24 - 1.12)	**0.10**<br>**(0.01 - 0.85)**
**0.47**<br>**(0.23 - 0.97)**	Palivizumab	0.54<br>(0.27 - 1.07)
**0.23**<br>**(0.09 - 0.59)**	**0.50**<br>**(0.26 - 0.95)**	Placebo

© 2023 Sun M et al. *JAMA Network Open*.

Appendix table S14: league table of rate of ICU admission in random effects model

Motavizumab	0.52 (0.24 - 1.12)	**0.10 (0.01 - 0.85)**
**0.47 (0.23 - 0.97)**	Palivizumab	0.54 (0.27 - 1.07)
**0.23 (0.09 - 0.59)**	**0.50 (0.26 - 0.95)**	Placebo

© 2023 Sun M et al. *JAMA Network Open*.

Appendix table S15: league table of rate of mechanical ventilation use in fixed effects model

Motavizumab	**0.18 (0.04 - 0.81)**	0.25 (0.02 - 2.76)
**0.23 (0.06 - 0.83)**	Palivizumab	0.57 (0.24 - 1.38)
**0.14 (0.03 - 0.57)**	0.62 (0.27 - 1.43)	Placebo

© 2023 Sun M et al. *JAMA Network Open*.

Appendix table S16: league table of rate of mechanical ventilation use in random effects model

Motavizumab	**0.18 (0.04 - 0.81)**	0.25 (0.02 - 2.76)
**0.23 (0.06 - 0.83)**	Palivizumab	0.57 (0.24 - 1.38)
**0.14 (0.03 - 0.57)**	0.62 (0.27 - 1.43)	Placebo

© 2023 Sun M et al. *JAMA Network Open*.

Appendix table S17: league table of drug-related adverse events in fixed effects model

M+P	0.76 (0.40 - 1.42)	.	.	.	.
0.76 (0.40 - 1.42)	Motavizumab	.	0.99 (0.69 - 1.42)	.	.
0.83 (0.30 - 2.32)	1.10 (0.49 - 2.47)	Nirsevimab	.	0.93 (0.51 - 1.68)	.
0.75 (0.36 - 1.54)	0.99 (0.69 - 1.42)	0.90 (0.44 - 1.85)	Palivizumab	1.03 (0.68 - 1.56)	.
0.77 (0.34 - 1.77)	1.02 (0.59 - 1.76)	0.93 (0.51 - 1.68)	1.03 (0.68 - 1.56)	Placebo	0.99 (0.60 - 1.65)
0.77 (0.29 - 2.03)	1.01 (0.48 - 2.14)	0.92 (0.42 - 2.01)	1.02 (0.53 - 1.97)	0.99 (0.60 - 1.65)	Suptavumab

© 2023 Sun M et al. *JAMA Network Open*.

Appendix table S18: league table of drug-related adverse events in random effects model

M+P	0.76 (0.40 - 1.42)	.	.	.	.
0.76 (0.40 - 1.42)	Motavizumab	.	0.99 (0.69 - 1.42)	.	.
0.83 (0.30 - 2.32)	1.10 (0.49 - 2.47)	Nirsevimab	.	0.93 (0.51 - 1.68)	.
0.75 (0.36 - 1.54)	0.99 (0.69 - 1.42)	0.90 (0.44 - 1.85)	Palivizumab	1.03 (0.68 - 1.56)	.
0.77 (0.34 - 1.77)	1.02 (0.59 - 1.76)	0.93 (0.51 - 1.68)	1.03 (0.68 - 1.56)	Placebo	0.99 (0.60 - 1.65)
0.77 (0.29 - 2.03)	1.01 (0.48 - 2.14)	0.92 (0.42 - 2.01)	1.02 (0.53 - 1.97)	0.99 (0.60 - 1.65)	Suptavumab

© 2023 Sun M et al. *JAMA Network Open*.

Appendix table S19: league table of rate of supplemental oxygen use in fixed effects model

Motavizumab	.	0.64 (0.39 - 1.06)	**0.12 (0.07 - 0.19)**
0.90 (0.26 - 3.04)	Nirsevimab	.	**0.13 (0.04 - 0.39)**
0.64 (0.39 - 1.06)	0.72 (0.19 - 2.68)	Palivizumab	.
**0.12 (0.07 - 0.19)**	**0.13 (0.04 - 0.39)**	**0.18 (0.09 - 0.37)**	Placebo

© 2023 Sun M et al. *JAMA Network Open*.

Appendix table S20: league table of rate of supplemental oxygen use in random effects model

Motavizumab	.	0.64<br>(0.39 - 1.06)	**0.12**<br>**(0.07 - 0.19)**
0.90<br>(0.26 - 3.04)	Nirsevimab	.	**0.13**<br>**(0.04 - 0.39)**
0.64<br>(0.39 - 1.06)	0.72<br>(0.19 - 2.68)	Palivizumab	.
**0.12**<br>**(0.07 - 0.19)**	**0.13**<br>**(0.04 - 0.39)**	**0.18**<br>**(0.09 - 0.37)**	Placebo

© 2023 Sun M et al. *JAMA Network Open*.

Appendix table S21: cumulative ranking of interventions for different outcomes

Strategies were ranked according to P score, which is the mean extent of certainty that one intervention is better than another, averaged over all competing interventions.

Outcome	Rank	Intervention
Rate of RSV infection	1	Motavizumab
	2	Nirsevimab
	3	Palivizumab
	4	Placebo
	5	Suptavumab
All-cause mortality	1	Nirsevimab
	2	Palivizumab
	3	Motavizumab
	4	M+P
	5	Placebo
RSV-related hospitalization	1	Nirsevimab
	2	Motavizumab
	3	Palivizumab
	4	Suptavumab
	5	Placebo
Rate of supplemental oxygen use	1	Motavizumab
	2	Nirsevimab
	3	Palivizumab
	4	Placebo
Rate of mechanical ventilation use	1	Motavizumab
	2	Palivizumab
	3	Placebo
Rate of ICU admission	1	Motavizumab
	2	Palivizumab
	3	Placebo
Drug-related adverse events	1	M+P
	2	Nirsevimab
	3	Placebo
	4	Suptavumab
	5	Motavizumab
	6	Palivizumab

© 2023 Sun M et al. *JAMA Network Open*.

Appendix table S22: details of GRADE assessment in all-cause mortality

**All-cause mortality**						
Comparison	Direct odds ratio (95% CI)	Certainty of evidence without imprecision	Indirect odds ratio (95% CI)	Certainty of evidence without imprecision	Network odds ratio (95% CI)	Certainty of evidence
Nirsevimab vs. Placebo	0.33 [0.05, 1.97]	High	——	——	0.33 [0.05, 1.97]	Moderate‡
Motavizumab vs. Placebo	1.00 [0.18, 5.48]	High	0.62 [0.22, 1.78]	High	0.71 [0.29, 1.74]	Moderate‡
Palivizumab vs. Placebo	0.70 [0.41, 1.19]	High	1.13 [0.16, 7.73]	High	0.72 [0.43, 1.21]	Moderate‡
Suptavumab vs. Placebo	1.37 [0.06, 33.61]	Moderate*	——	——	1.37 [0.06, 33.61]	Low§
Motavizumab vs. Palivizumab	0.89 [0.36, 2.20]	High	1.43 [0.24, 8.49]	High	0.98 [0.44, 2.20]	Moderate‡
M+P vs. Motavizumab	1.12 [0.10, 12.54]	Moderate†	——	——	1.12 [0.10, 12.54]	Low§
M+P vs. Nirsevimab	——	——	2.43 [0.11, 55.88]	Moderate†	2.43 [0.11, 55.88]	Low§
M+P vs. Palivizumab	——	——	1.10 [0.09, 14.04]	Moderate†	1.10 [0.09, 14.04]	Low§
M+P vs. Placebo	——	——	0.80 [0.06, 10.46]	Moderate†	0.80 [0.06, 10.46]	Low§
Motavizumab vs. Nirsevimab	——	——	2.16 [0.29, 16.03]	High	2.16 [0.29, 16.03]	Moderate‡
Nirsevimab vs. Palivizumab	——	——	0.45 [0.07, 2.93]	High	0.45 [0.07, 2.93]	Moderate‡
Suptavumab vs. M+P	——	——	——	——	——	——
Suptavumab vs. Motavizumab	——	——	——	——	——	——
Suptavumab vs. Nirsevimab	——	——	——	——	——	——
Suptavumab vs. Palivizumab	——	——	——	——	——	——

*Downgraded 1 level due to risk of bias.

†Downgraded 1 level due to risk of bias.

‡Downgraded 1 level due to imprecision.

§Downgraded 2 level due to imprecision and risk of bias.

© 2023 Sun M et al. *JAMA Network Open*.

Appendix table S23: details of GRADE assessment in RSV-related hospitalization

RSV-related hospitalization						
Comparison	Direct odds ratio (95% CI)	Certainty of evidence without imprecision	Indirect odds ratio (95% CI)	Certainty of evidence without imprecision	Network odds ratio (95% CI)	Certainty of evidence
Nirsevimab vs. Placebo	0.25 [0.13, 0.47]	High	——	——	0.25 [0.13, 0.47]	High
Palivizumab vs. Placebo	0.45 [0.34, 0.60]	High	——	——	0.45 [0.34, 0.60]	High
Motavizumab vs. Palivizumab	0.73 [0.50, 1.08]	High	——	——	0.73 [0.50, 1.08]	Moderate†
Suptavumab vs. Placebo	1.00 [0.52, 1.92]	Moderate*	——	——	1.00 [0.52, 1.92]	Low‡
Motavizumab vs. Nirsevimab	——	——	1.34 [0.60, 3.01]	High	1.34 [0.60, 3.01]	Moderate†
Motavizumab vs. Placebo	——	——	0.33 [0.20, 0.53]	High	0.33 [0.20, 0.53]	High
Motavizumab vs. Suptavumab	——	——	0.33 [0.15, 0.74]	Moderate*	0.33 [0.15, 0.74]	Moderate*
Nirsevimab vs. Palivizumab	——	——	0.55 [0.27, 1.12]	High	0.55 [0.27, 1.12]	Moderate†
Nirsevimab vs. Suptavumab	——	——	0.25 [0.10, 0.62]	Moderate*	0.25 [0.10, 0.62]	Moderate*
Palivizumab vs. Suptavumab	——	——	0.45 [0.22, 0.92]	Moderate*	0.45 [0.22, 0.92]	Moderate*

*Downgraded 1 level due to risk of bias.

†Downgraded 1 level due to imprecision.

‡Downgraded 2 level due to imprecision and risk of bias.

© 2023 Sun M et al. *JAMA Network Open*.

Appendix table S24: details of GRADE assessment in rate of RSV infection

Rate of RSV infection						
Comparison	Direct odds ratio (95% CI)	Certainty of evidence without imprecision	Indirect odds ratio (95% CI)	Certainty of evidence without imprecision	Network odds ratio (95% CI)	Certainty of evidence
Nirsevimab vs. Placebo	0.24 [0.16, 0.37]	High	——	——	0.24 [0.16, 0.37]	High
Motavizumab vs. Placebo	0.17 [0.12, 0.23]	High	——	——	0.17 [0.12, 0.23]	Moderate‡
Palivizumab vs. Placebo	0.32 [0.22, 0.47]	Moderate*	——	——	0.32 [0.22, 0.47]	Moderate*
Suptavumab vs. Placebo	1.06 [0.73, 1.53]	Moderate*	——	——	1.06 [0.73, 1.53]	Low†
Motavizumab vs. Nirsevimab	——	——	0.68 [0.41, 1.14]	High	0.68 [0.41, 1.14]	Moderate‡
Motavizumab vs. Palivizumab	——	——	0.52 [0.32, 0.86]	Moderate*	0.52 [0.32, 0.86]	Moderate*
Motavizumab vs. Suptavumab	——	——	0.16 [0.10, 0.25]	Moderate*	0.16 [0.10, 0.25]	Moderate*
Nirsevimab vs. Palivizumab	——	——	0.77 [0.44, 1.35]	Moderate*	0.77 [0.44, 1.35]	Low†
Nirsevimab vs. Suptavumab	——	——	0.23 [0.13, 0.40]	Moderate*	0.23 [0.13, 0.40]	Moderate*
Palivizumab vs. Suptavumab	——	——	0.30 [0.18, 0.51]	Moderate*	0.30 [0.18, 0.51]	Moderate*

*Downgraded 1 level due to risk of bias.

†Downgraded 2 level due to risk of bias and imprecision.

‡Downgraded 1 level due to imprecision.

© 2023 Sun M et al. *JAMA Network Open*.

Appendix table S25: details of GRADE assessment in drug-related adverse events

**Drug-related AE**						
Comparison	Direct odds ratio (95% CI)	Certainty of evidence without imprecision	Indirect odds ratio (95% CI)	Certainty of evidence without imprecision	Network odds ratio (95% CI)	Certainty of evidence
Nirsevimab vs. Placebo	0.93 [0.51, 1.68]	High	——	——	0.93 [0.51, 1.68]	Moderate†
Palivizumab vs. Placebo	1.03 [0.68, 1.56]	High	——	——	1.03 [0.68, 1.56]	Moderate†
Motavizumab vs. Palivizumab	0.99 [0.69, 1.42]	High	——	——	0.99 [0.69, 1.42]	Moderate†
M+P vs. Motavizumab	0.76 [0.40, 1.42]	Moderate*	——	——	0.76 [0.40, 1.42]	Low‡
Suptavumab vs. Placebo	1.01 [0.61, 1.68]	Moderate*	——	——	1.01 [0.61, 1.68]	Low‡
M+P vs. Nirsevimab	——	——	0.83 [0.30, 2.32]	Moderate*	0.83 [0.30, 2.32]	Low‡
M+P vs. Palivizumab	——	——	0.75 [0.36, 1.54]	Moderate*	0.75 [0.36, 1.54]	Low‡
M+P vs. Placebo	——	——	0.77 [0.34, 1.77]	Moderate*	0.77 [0.34, 1.77]	Low‡
M+P vs. Suptavumab	——	——	0.77 [0.29, 2.03]	Moderate*	0.77 [0.29, 2.03]	Low‡
Motavizumab vs. Nirsevimab	——	——	1.10 [0.49, 2.47]	High	1.10 [0.49, 2.47]	Moderate†
Motavizumab vs. Placebo	——	——	1.02 [0.59, 1.76]	High	1.02 [0.59, 1.76]	Moderate†
Motavizumab vs. Suptavumab	——	——	1.01 [0.48, 2.14]	Moderate*	1.01 [0.48, 2.14]	Low‡
Nirsevimab vs. Palivizumab	——	——	0.90 [0.44, 1.85]	High	0.90 [0.44, 1.85]	Moderate†
Nirsevimab vs. Suptavumab	——	——	0.92 [0.42, 2.01]	Moderate*	0.92 [0.42, 2.01]	Low‡
Palivizumab vs. Suptavumab	——	——	1.02 [0.53, 1.97]	Moderate*	1.02 [0.53, 1.97]	Low‡

*Downgraded 1 level due to risk of bias.

†Downgraded 1 level due to imprecision.

‡Downgraded 2 level due to imprecision and risk of bias.

© 2023 Sun M et al. *JAMA Network Open*.

Appendix table S26: details of GRADE assessment in supplemental oxygen use

**Rate of supplemental oxygen use**						
Comparison	Direct odds ratio (95% CI)	Certainty of evidence without imprecision	Indirect odds ratio (95% CI)	Certainty of evidence without imprecision	Network odds ratio (95% CI)	Certainty of evidence
Nirsevimab vs. Placebo	0.13 [0.04, 0.39]	High	——	——	0.13 [0.04, 0.39]	Moderate*
Motavizumab vs. Placebo	0.12 [0.07, 0.19]	High	——	——	0.12 [0.07, 0.19]	Moderate*
Motavizumab vs. Palivizumab	0.64 [0.39, 1.06]	High	——	——	0.64 [0.39, 1.06]	Moderate*
Motavizumab vs. Nirsevimab	——	——	0.90 [0.26, 3.04]	High	0.90 [0.26, 3.04]	Moderate*
Nirsevimab vs. Palivizumab	——	——	0.72 [0.19, 2.68]	High	0.72 [0.19, 2.68]	Moderate*
Palivizumab vs. Placebo	——	——	0.18 [0.09, 0.37]	High	0.18 [0.09, 0.37]	Moderate*

*Downgraded 1 level due to imprecision.

© 2023 Sun M et al. *JAMA Network Open*.

Appendix table S27: details of GRADE assessment in rate of mechanical ventilation use

**Rate of MV use**						
Comparison	Direct odds ratio (95% CI)	Certainty of evidence without imprecision	Indirect odds ratio (95% CI)	Certainty of evidence without imprecision	Network odds ratio (95% CI)	Certainty of evidence
Motavizumab vs. Placebo	0.25 [0.02, 2.76]	High	0.10 [0.02, 0.59]	High	0.14 [0.03, 0.57]	Moderate*
Palivizumab vs. Placebo	0.57 [0.24, 1.38]	High	1.39 [0.08, 23.67]	High	0.62 [0.27, 1.43]	Moderate*
Nirsevimab vs. Placebo	0.16 [0.01, 4.05]	High	——	——	0.16 [0.01, 4.05]	Moderate*
Motavizumab vs. Palivizumab	0.18 [0.04, 0.81]	High	0.44 [0.03, 5.61]	High	0.23 [0.06, 0.83]	Moderate*
Motavizumab vs. Nirsevimab	——	——	——	——	——	——
Palivizumab vs. Nirsevimab	——	——	——	——	——	——

*Downgraded 1 level due to imprecision.

© 2023 Sun M et al. *JAMA Network Open*.

Appendix table S28: details of GRADE assessment in rate of ICU admission

**Rate of ICU admission**						
Comparison	Direct odds ratio (95% CI)	Certainty of evidence without imprecision	Indirect odds ratio (95% CI)	Certainty of evidence without imprecision	Network odds ratio (95% CI)	Certainty of evidence
Motavizumab vs. Placebo	0.10 [0.01, 0.85]	High	0.28 [0.10, 0.79]	High	0.23 [0.09, 0.59]	Moderate*
Palivizumab vs. Placebo	0.54 [0.27, 1.07]	High	0.19 [0.02, 1.87]	High	0.50 [0.26, 0.95]	Moderate*
Nirsevimab vs. Placebo	0.04 [0.00, 0.81]	High	——	——	0.04 [0.00, 0.81]	Moderate*
Motavizumab vs. Palivizumab	0.52 [0.24, 1.12]	High	0.18 [0.02, 1.76]	High	0.47 [0.23, 0.97]	Moderate*
Motavizumab vs. Nirsevimab	——	——	——	——	——	——
Palivizumab vs. Nirsevimab	——	——	——	——	——	——

*Downgraded 1 level due to imprecision.

© 2023 Sun M et al. *JAMA Network Open*.

Appendix table S29: details of GRADE assessment in drug-related serious adverse events

Drug-related SAE		
Comparison	Direct odds ratio (95% CI)	Certainty of evidence
Motavizumab vs. Placebo	8.56 [0.49, 148.58]	Moderate*
Palivizumab vs. Placebo	0.14 [0.01, 2.80]	Moderate*
Nirsevimab vs. Placebo	——	——
Motavizumab vs. Palivizumab	0.82 [0.25, 2.71]	Moderate*
M+P vs. Motavizumab	1.12 [0.10, 12.54]	Low†

*Downgraded 1 level due to imprecision.

†Downgraded 2 level due to imprecision and risk of bias.

© 2023 Sun M et al. *JAMA Network Open*.

Appendix table S30-S45: Bayesian league tables of outcomes in fixed and random models

Outcomes were reported as odds ratio (95% credible interval). All tables list the treatments in alphabetical order. The estimate is in the cell in common between the column-defining intervention and the row-defining intervention. The league table contains pairwise comparisons of the treatment in the row versus the treatment in the column in the lower triangle and column versus row in the upper triangle.

© 2023 Sun M et al. *JAMA Network Open*.

Appendix table S30: Bayesian league table of rate of RSV infection in random effects model

Motavizumab	1.45 (0.37, 5.65)	1.97 (0.58, 7.97)	6.02 (2.02, 17.99)	6.40 (1.37, 30.44)
0.69 (0.18, 2.72)	Nirsevimab	1.36 (0.49, 4.48)	4.14 (1.83, 9.60)	4.40 (1.13, 17.82)
0.51 (0.13, 1.73)	0.74 (0.22, 2.04)	Palivizumab	3.05 (1.36, 5.94)	3.26 (0.81, 11.29)
0.17 (0.06, 0.50)	0.24 (0.10, 0.55)	0.33 (0.17, 0.74)	Placebo	1.06 (0.36, 3.23)
0.16 (0.03, 0.73)	0.23 (0.06, 0.88)	0.31 (0.09, 1.24)	0.94 (0.31, 2.79)	Suptavumab

© 2023 Sun M et al. *JAMA Network Open*.

Appendix table S31: Bayesian league table of rate of RSV infection in fixed effects model

Motavizumab	1.47 (0.87, 2.45)	1.91 (1.16, 3.13)	6.03 (4.43, 8.31)	6.42 (3.97, 10.45)
0.68 (0.41, 1.15)	Nirsevimab	1.30 (0.74, 2.28)	4.11 (2.75, 6.22)	4.37 (2.55, 7.64)
0.53 (0.32, 0.86)	0.77 (0.44, 1.35)	Palivizumab	3.17 (2.17, 4.65)	3.37 (2.00, 5.76)
0.17 (0.12, 0.23)	0.24 (0.16, 0.36)	0.32 (0.21, 0.46)	Placebo	1.06 (0.74, 1.54)
0.16 (0.1, 0.25)	0.23 (0.13, 0.39)	0.30 (0.17, 0.50)	0.94 (0.65, 1.35)	Suptavumab

© 2023 Sun M et al. *JAMA Network Open*.

Appendix table S32: Bayesian league table of all-cause mortality in random effects model

M+P	0.73 (0.02, 9.32)	0.91 (0.02, 21.63)	0.72 (0.02, 10.7)	1.04 (0.03, 15.68)	113751.74 (0.19, 1764267809536012800)
1.38 (0.11, 43.94)	Motavizumab	1.28 (0.23, 8.13)	1 (0.44, 2.28)	1.45 (0.58, 3.55)	171012.94 (0.49, 2485624818560194560)
1.09 (0.05, 49.59)	0.78 (0.12, 4.39)	Nirsevimab	0.79 (0.14, 3.69)	1.14 (0.22, 4.86)	130929.84 (0.34, 1885003463665145600)
1.38 (0.09, 47.81)	1 (0.44, 2.26)	1.26 (0.27, 7.04)	Palivizumab	1.44 (0.86, 2.43)	169161.91 (0.51, 2442958399732975616)
0.96 (0.06, 33.48)	0.69 (0.28, 1.71)	0.88 (0.21, 4.53)	0.69 (0.41, 1.16)	Placebo	117377.89 (0.36, 1628536804196702720)
0 (0, 5.4)	0 (0, 2.05)	0 (0, 2.94)	0 (0, 1.96)	0 (0, 2.77)	Suptavumab

© 2023 Sun M et al. *JAMA Network Open*.

Appendix table S33: Bayesian league table of all-cause mortality in fixed effects model

M+P	0.73 (0.02, 9.32)	0.91 (0.02, 21.63)	0.72 (0.02, 10.7)	1.04 (0.03, 15.68)	113751.74 (0.19, 1764267809536012800)
1.38 (0.11, 43.94)	Motavizumab	1.28 (0.23, 8.13)	1 (0.44, 2.28)	1.45 (0.58, 3.55)	171012.94 (0.49, 2485624818560194560)
1.09 (0.05, 49.59)	0.78 (0.12, 4.39)	Nirsevimab	0.79 (0.14, 3.69)	1.14 (0.22, 4.86)	130929.84 (0.34, 1885003463665145600)
1.38 (0.09, 47.81)	1 (0.44, 2.26)	1.26 (0.27, 7.04)	Palivizumab	1.44 (0.86, 2.43)	169161.91 (0.51, 2442958399732975616)
0.96 (0.06, 33.48)	0.69 (0.28, 1.71)	0.88 (0.21, 4.53)	0.69 (0.41, 1.16)	Placebo	117377.89 (0.36, 1628536804196702720)
0 (0, 5.4)	0 (0, 2.05)	0 (0, 2.94)	0 (0, 1.96)	0 (0, 2.77)	Suptavumab

© 2023 Sun M et al. *JAMA Network Open*.

Appendix table S34: Bayesian league table of RSV-related hospitalization in random effects model

Motavizumab	1.52<br>(0.02, 260.25)	1.37<br>(0.04, 51.21)	5.9<br>(0.16, 472.6)	6.02<br>(0.04, 1972.77)
0.66<br>(0, 52.44)	Nirsevimab	0.88<br>(0.03, 14.59)	3.91<br>(0.3, 53.02)	3.98<br>(0.05, 344.37)
0.73<br>(0.02, 25.9)	1.13<br>(0.07, 36.1)	Palivizumab	4.43<br>(1.06, 35.88)	4.44<br>(0.11, 357.65)
0.17<br>(0, 6.29)	0.26<br>(0.02, 3.38)	0.23<br>(0.03, 0.94)	Placebo	1.01<br>(0.03, 38.5)
0.17<br>(0, 25.25)	0.25<br>(0, 20.55)	0.23<br>(0, 8.76)	0.99<br>(0.03, 36.09)	Suptavumab

© 2023 Sun M et al. *JAMA Network Open*.

Appendix table S35: Bayesian league table of RSV-related hospitalization in fixed effects model

Motavizumab	0.84 (0.37, 1.87)	1.37 (0.93, 2.01)	3.5 (2.17, 5.65)	3.54 (1.59, 8.16)
1.19 (0.54, 2.73)	Nirsevimab	1.62 (0.81, 3.4)	4.15 (2.2, 8.21)	4.22 (1.69, 11.05)
0.73 (0.5, 1.07)	0.62 (0.29, 1.24)	Palivizumab	2.56 (1.94, 3.39)	2.59 (1.28, 5.43)
0.29 (0.18, 0.46)	0.24 (0.12, 0.45)	0.39 (0.29, 0.52)	Placebo	1.01 (0.53, 2.01)
0.28 (0.12, 0.63)	0.24 (0.09, 0.59)	0.39 (0.18, 0.78)	0.99 (0.5, 1.88)	Suptavumab

© 2023 Sun M et al. *JAMA Network Open*.

Appendix table S36: Bayesian league table of rate of supplemental oxygen use in random effects model

Motavizumab	1.04<br>(0.02, 54.33)	1.55<br>(0.1, 24.05)	8.84<br>(0.57, 135.5)
0.96<br>(0.02, 52.12)	Nirsevimab	1.5<br>(0.01, 191.09)	8.43<br>(0.49, 154.15)
0.64<br>(0.04, 9.81)	0.67<br>(0.01, 84.42)	Palivizumab	5.68<br>(0.12, 270.02)
0.11<br>(0.01, 1.75)	0.12<br>(0.01, 2.06)	0.18<br>(0, 8.5)	Placebo

© 2023 Sun M et al. *JAMA Network Open*.

Appendix table S37: Bayesian league table of rate of supplemental oxygen use in fixed effects model

Motavizumab	1.06<br>(0.26, 3.45)	1.56<br>(0.95, 2.6)	8.79<br>(5.37, 15.11)
0.94<br>(0.29, 3.82)	Nirsevimab	1.48<br>(0.41, 6.49)	8.27<br>(2.91, 30.8)
0.64<br>(0.38, 1.05)	0.68<br>(0.15, 2.45)	Palivizumab	5.65<br>(2.77, 11.7)
0.11<br>(0.07, 0.19)	0.12<br>(0.03, 0.34)	0.18<br>(0.09, 0.36)	Placebo

© 2023 Sun M et al. *JAMA Network Open*.

Appendix table S38: Bayesian league table of rate of mechanical ventilation use in random effects model

Motavizumab	0<br>(0, 11.96)	5.03<br>(0.63, 45.73)	7.7<br>(0.74, 83.84)
24058.21<br>(0.08, 855629723170699904)	Nirsevimab	122529.85<br>(0.48, 4252162126839230976)	183653.94<br>(0.87, 5821885953627409408)
0.2<br>(0.02, 1.59)	0<br>(0, 2.1)	Palivizumab	1.54<br>(0.21, 10.2)
0.13<br>(0.01, 1.35)	0<br>(0, 1.16)	0.65<br>(0.1, 4.82)	Placebo

© 2023 Sun M et al. *JAMA Network Open*.

Appendix table S39: Bayesian league table of rate of mechanical ventilation use in fixed effects model

Motavizumab	0<br>(0, 7.71)	4.96<br>(1.52, 23.27)	8.27<br>(2.12, 43.62)
26691.14<br>(0.13, 56102833244432992)	Nirsevimab	138661.44<br>(0.76, 309977006714859136)	227408.63<br>(1.31, 518393465197570240)
0.2<br>(0.04, 0.66)	0<br>(0, 1.32)	Palivizumab	1.64<br>(0.72, 3.96)
0.12<br>(0.02, 0.47)	0<br>(0, 0.76)	0.61<br>(0.25, 1.4)	Placebo

© 2023 Sun M et al. *JAMA Network Open*.

Appendix table S40: Bayesian league table of rate of ICU admission in random effects model

Motavizumab	0<br>(0, 0.35)	2.53<br>(0.17, 54.84)	6.12<br>(0.4, 191.16)
5847429934.38<br>(2.86, 1.673431187075e+26)	Nirsevimab	15616450915.69<br>(8.23, 4.51961657999075e+26)	40371913834.37<br>(25.82, 1.03437981491577e+27)
0.4<br>(0.02, 5.99)	0<br>(0, 0.12)	Palivizumab	2.39<br>(0.16, 50.19)
0.16<br>(0.01, 2.5)	0<br>(0, 0.04)	0.42<br>(0.02, 6.26)	Placebo

© 2023 Sun M et al. *JAMA Network Open*.

Appendix table S41: Bayesian league table of rate of ICU admission in fixed effects model

Motavizumab	0<br>(0, 0.14)	2.22<br>(1.1, 4.81)	4.66<br>(1.92, 12.04)
217989677350.61<br>(6.9, 1.01180718649146e+31)	Nirsevimab	490291654606.98<br>(15.84, 2.24726925127156e+31)	1025081497855.31<br>(33.16, 4.47461804254501e+31)
0.45<br>(0.21, 0.91)	0<br>(0, 0.06)	Palivizumab	2.08<br>(1.1, 4.17)
0.21<br>(0.08, 0.52)	0<br>(0, 0.03)	0.48<br>(0.24, 0.91)	Placebo

© 2023 Sun M et al. *JAMA Network Open*.

Appendix table S42: Bayesian league table of drug-related adverse events in random effects model

M+P	1.32 (0.43, 3.99)	1.34 (0.23, 8.62)	1.29 (0.32, 4.76)	1.25 (0.24, 5.96)	1.27 (0.18, 8.34)
0.76 (0.25, 2.32)	Motavizumab	1.02 (0.26, 4.49)	0.98 (0.43, 2.04)	0.95 (0.29, 2.9)	0.96 (0.19, 4.45)
0.75 (0.12, 4.3)	0.98 (0.22, 3.92)	Nirsevimab	0.96 (0.26, 2.93)	0.93 (0.37, 2.02)	0.95 (0.23, 3.38)
0.78 (0.21, 3.17)	1.02 (0.49, 2.34)	1.04 (0.34, 3.82)	Palivizumab	0.97 (0.42, 2.32)	0.99 (0.26, 3.93)
0.8 (0.17, 4.13)	1.06 (0.34, 3.48)	1.07 (0.49, 2.69)	1.03 (0.43, 2.4)	Placebo	1.02 (0.35, 2.93)
0.79 (0.12, 5.58)	1.04 (0.22, 5.15)	1.05 (0.3, 4.39)	1.01 (0.25, 3.91)	0.99 (0.34, 2.83)	Suptavumab

© 2023 Sun M et al. *JAMA Network Open*.

Appendix table S43: Bayesian league table of drug-related adverse events in fixed effects model

M+P	1.32<br>(0.69, 2.46)	1.34<br>(0.48, 3.75)	1.33<br>(0.64, 2.74)	1.29<br>(0.55, 2.96)	1.31<br>(0.49, 3.49)
0.76<br>(0.41, 1.44)	Motavizumab	1.01<br>(0.46, 2.29)	1.01<br>(0.7, 1.45)	0.98<br>(0.56, 1.69)	0.99<br>(0.47, 2.12)
0.75<br>(0.27, 2.09)	0.99<br>(0.44, 2.18)	Nirsevimab	1<br>(0.48, 2.02)	0.97<br>(0.53, 1.71)	0.98<br>(0.45, 2.13)
0.75<br>(0.36, 1.57)	0.99<br>(0.69, 1.42)	1<br>(0.49, 2.09)	Palivizumab	0.97<br>(0.64, 1.46)	0.98<br>(0.51, 1.92)
0.78<br>(0.34, 1.8)	1.02<br>(0.59, 1.77)	1.03<br>(0.58, 1.9)	1.03<br>(0.68, 1.56)	Placebo	1.01<br>(0.61, 1.72)
0.76<br>(0.29, 2.04)	1.01<br>(0.47, 2.13)	1.02<br>(0.47, 2.24)	1.02<br>(0.52, 1.95)	0.99<br>(0.58, 1.63)	Suptavumab

© 2023 Sun M et al. *JAMA Network Open*.

Appendix table S44: Bayesian league table of drug-related serious adverse events in random effects model

M+P	0.71<br>(0.01, 48.35)	20222.9<br>(0, 1.84127748263721e+29)	0.17<br>(0, 26.53)	0.25<br>(0, 61.71)
1.4<br>(0.02, 147.72)	Motavizumab	31979.62<br>(0, 2.1964172568143e+29)	0.25<br>(0.01, 4.39)	0.37<br>(0.01, 12.1)
0<br>(0, 2985881300883712)	0<br>(0, 1697149564964730)	Nirsevimab	0<br>(0, 433416757509325)	0<br>(0, 511717295620109)
5.97<br>(0.04, 2028)	4.07<br>(0.23, 132.29)	142821.12<br>(0, 1.04766699009681e+30)	Palivizumab	1.41<br>(0.04, 84.76)
4.02<br>(0.02, 1803.64)	2.68<br>(0.08, 151.91)	94516.74<br>(0, 5.53272420784994e+29)	0.71<br>(0.01, 24.09)	Placebo

© 2023 Sun M et al. *JAMA Network Open*.

Appendix table S45: Bayesian league table of drug-related serious adverse events in fixed effects model

M+P	0.74<br>(0.02, 9.58)	14312.42<br>(0, 6.39254240399028e+28)	0.45<br>(0.01, 7.68)	0.33<br>(0.01, 6.43)
1.35<br>(0.1, 44.04)	Motavizumab	21367.07<br>(0, 8.86040823969649e+28)	0.63<br>(0.18, 1.96)	0.47<br>(0.09, 1.88)
0<br>(0, 1347470867106858)	0<br>(0, 784023877300891)	Nirsevimab	0<br>(0, 504596108636899)	0<br>(0, 346926025479572)
2.2<br>(0.13, 84.25)	1.59<br>(0.51, 5.41)	33999.92<br>(0, 1.38689577917869e+29)	Palivizumab	0.74<br>(0.13, 3.63)
3.03<br>(0.16, 134.87)	2.12<br>(0.53, 11.6)	47959.76<br>(0, 1.86085058214622e+29)	1.35<br>(0.28, 7.75)	Placebo

© 2023 Sun M et al. *JAMA Network Open*.

Appendix table S46: details of continuous outcomes

First author, year	Intervention	Comparison	Participants (I/C)	mean (I)	mean (C)
**Duration of MV use (total days/100 children)**					
**O'Brien KL, 2015**	Motavizumab	Placebo	1417/710	0.21	1.83
**Carbonell-Estrany, 2010**	Motavizumab	Palivizumab	3329/3306	0.5	3.8
**Feltes, 2003**	Palivizumab	Placebo	639/648	6.5	54.7
**Duration of RSV-related hospital stay (total days/100 children)**					
**O'Brien KL, 2015**	Motavizumab	Placebo	1417/710	7.41	52.96
**Carbonell-Estrany, 2010**	Motavizumab	Palivizumab	3329/3306	9.1	18.1
**Feltes, 2003**	Palivizumab	Placebo	639/648	57.4	129
**Griffin MP, 2020**	Nirsevimab	Placebo	969/484	5.37	28.93
**Duration of RSV ICU stay (total days/100 children)**					
**O'Brien KL, 2015**	Motavizumab	Placebo	1417/710	0.28	3.66
**Carbonell-Estrany, 2010**	Motavizumab	Palivizumab	3329/3306	2	6.3
**Feltes, 2003**	Palivizumab	Placebo	639/648	15.9	71.2
**Duration of supplemental oxygen use**					
**O'Brien KL, 2015**	Motavizumab	Placebo	1417/710	6.28	44.93
**Carbonell-Estrany, 2010**	Motavizumab	Palivizumab	3329/3306	4.1	9.5
**Feltes, 2003**	Palivizumab	Placebo	639/648	27.9	101.5

© 2023 Sun M et al. *JAMA Network Open*.

APPENDIX FIGURES

Appendix figure S1: risk of bias assessment

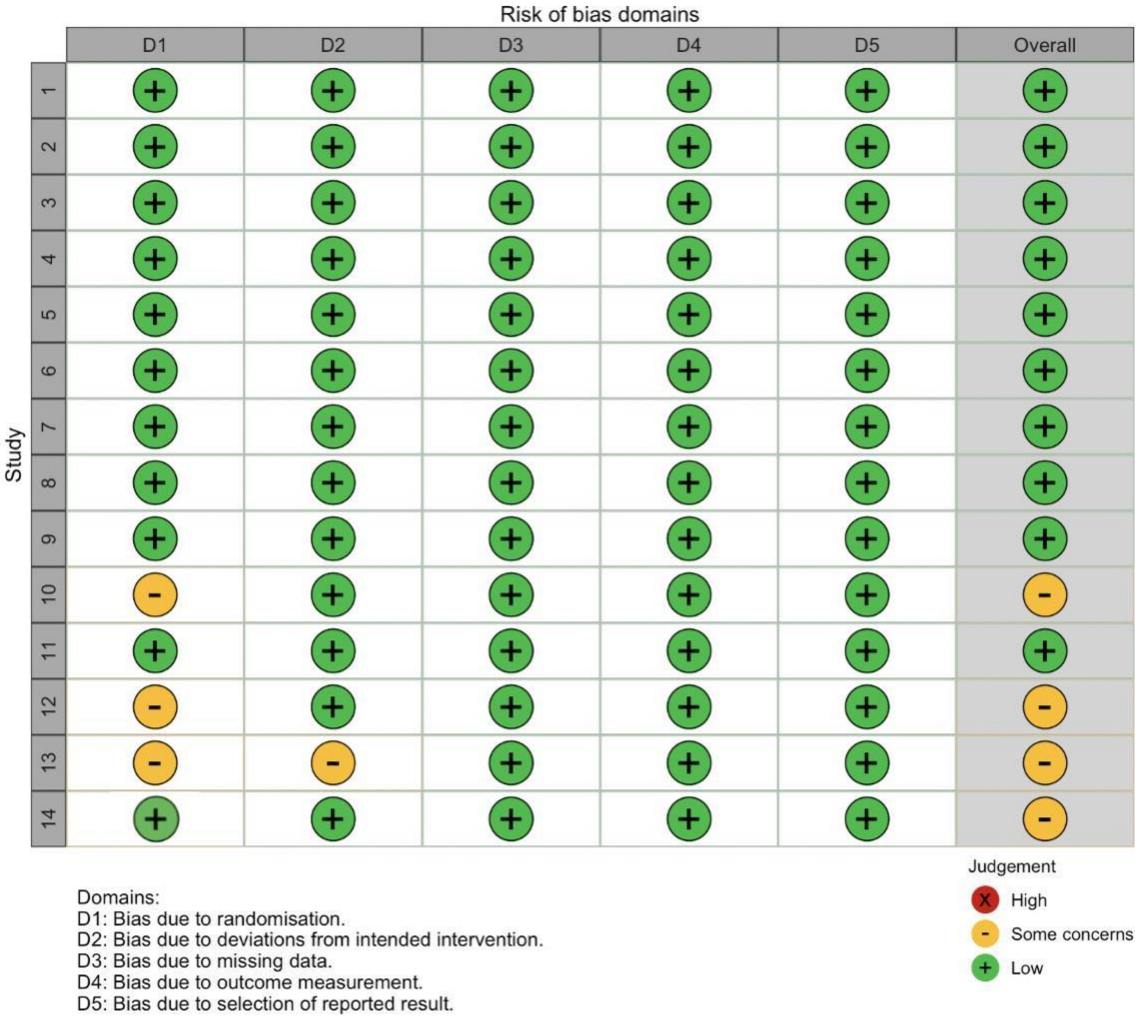

Appendix figure S1-1: risk of bias assessment for dichotomous outcome

© 2023 Sun M et al. *JAMA Network Open*.

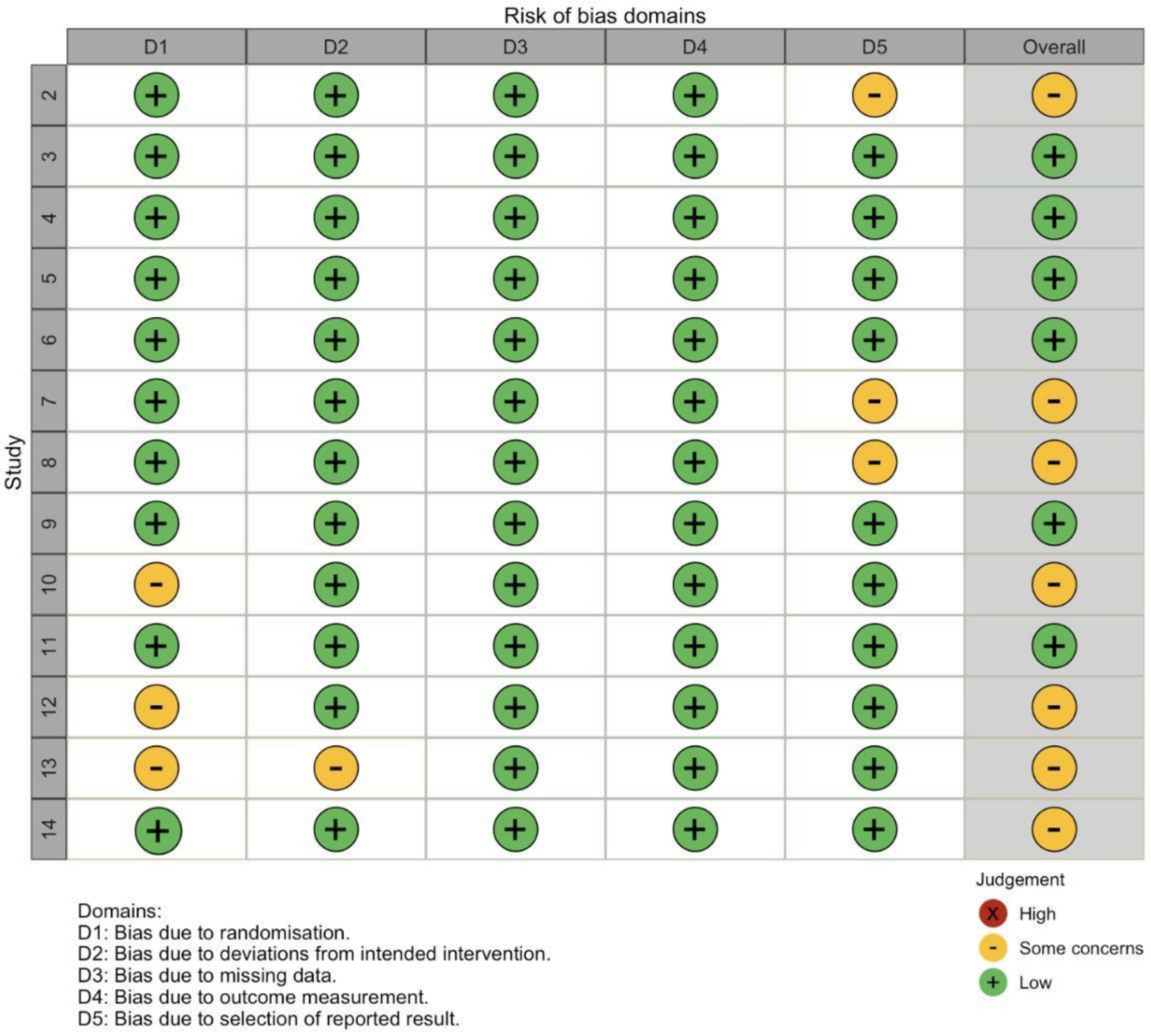

Appendix figure S1-2: risk of bias assessment for continuous outcome

© 2023 Sun M et al. *JAMA Network Open*.

Appendix figure S2-S9: network plots

Network plot comparing mAbs in preventing RSV in children. The line width is proportional to the number of studies comparing each pair of interventions, and the size of each node is proportional to the number of participants (sample size).

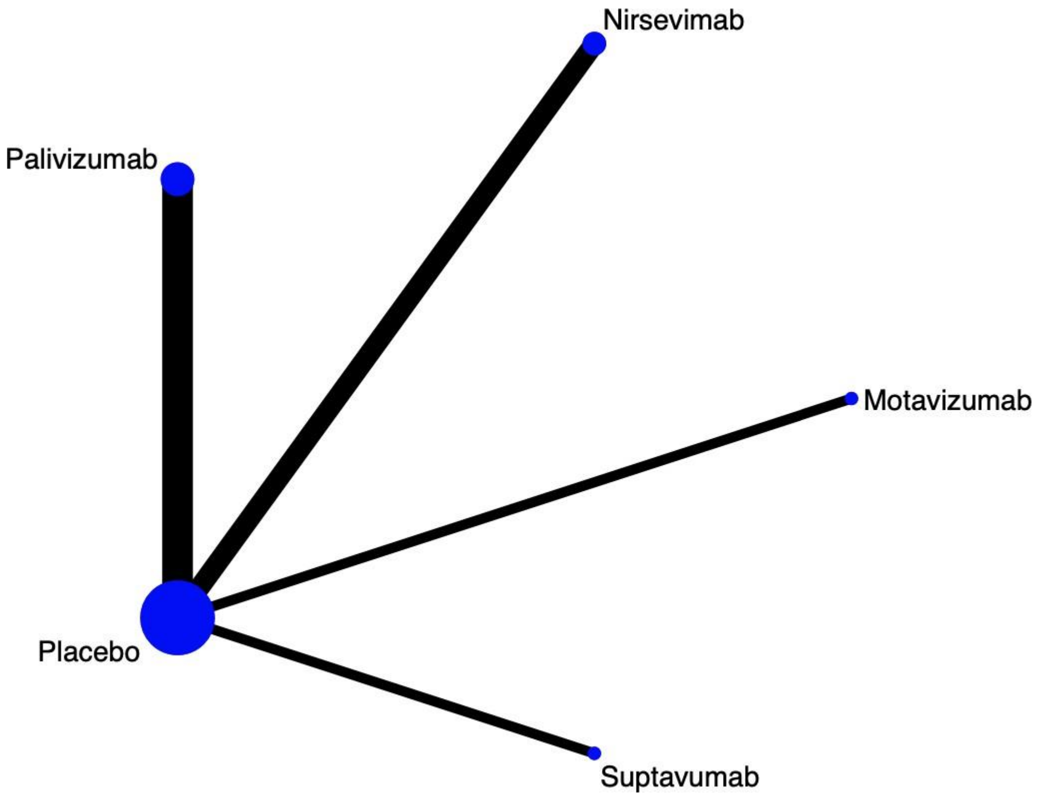

Appendix figure S2: network plot of rate of RSV infection

© 2023 Sun M et al. *JAMA Network Open*.

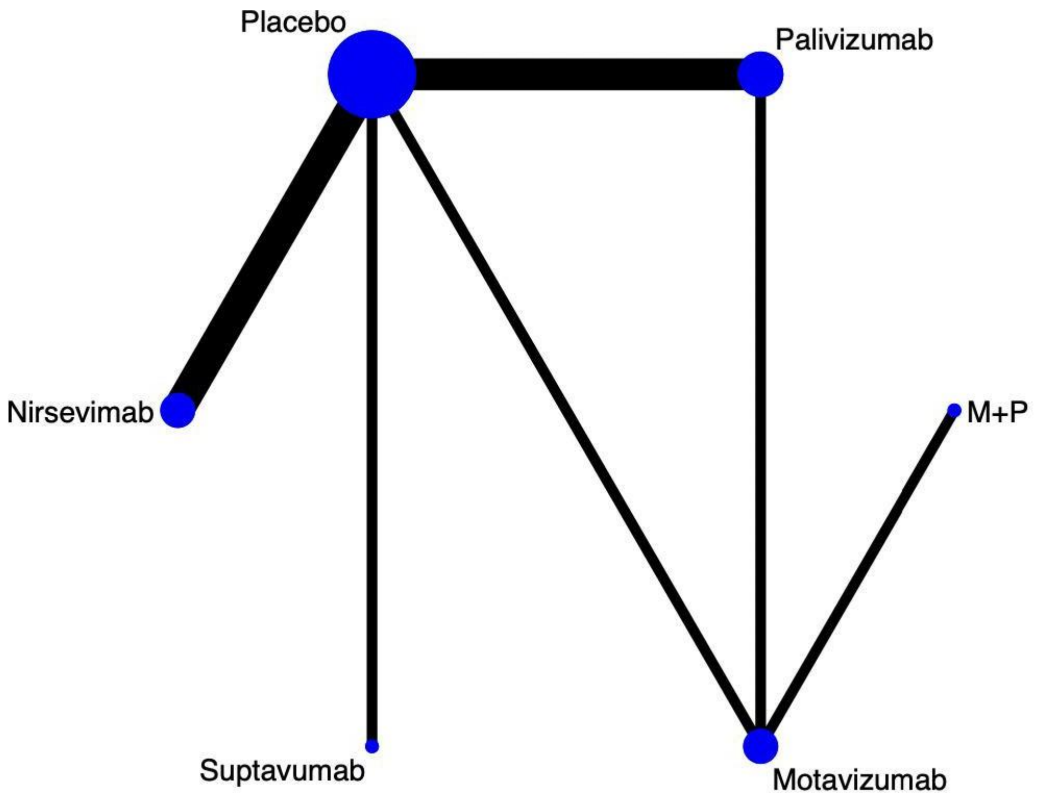

Appendix figure S3: network plot of all-cause mortality

© 2023 Sun M et al. *JAMA Network Open*.

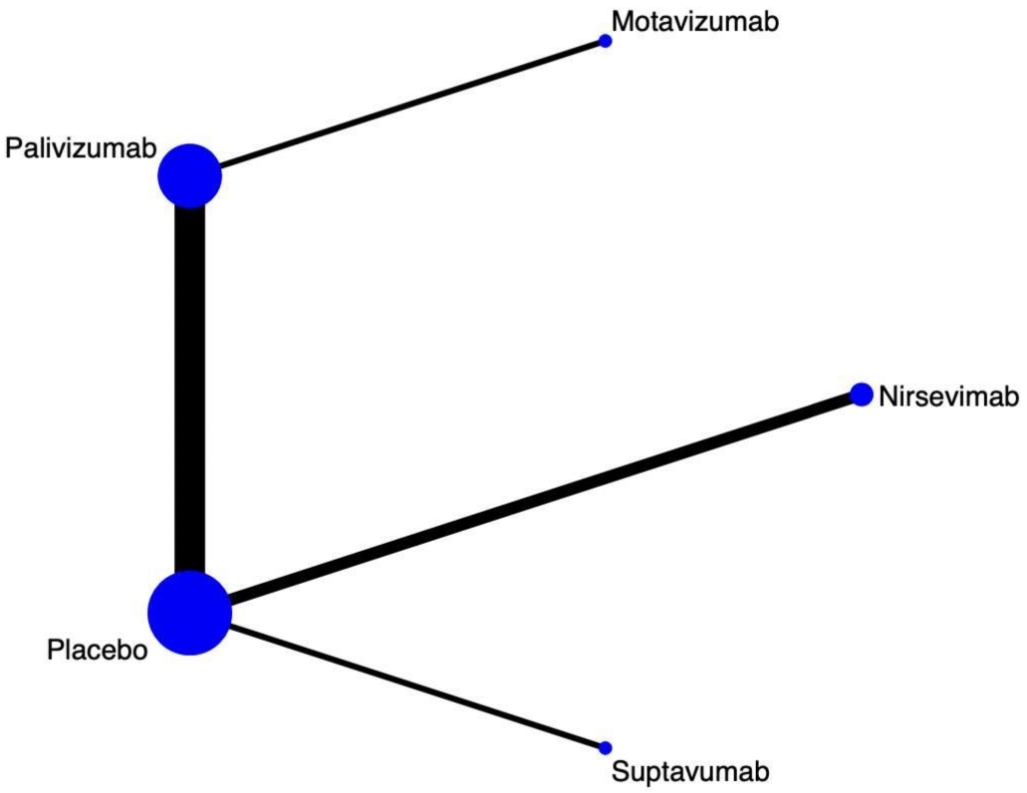

Appendix figure S4: network plot of RSV-related hospitalization

© 2023 Sun M et al. *JAMA Network Open*.

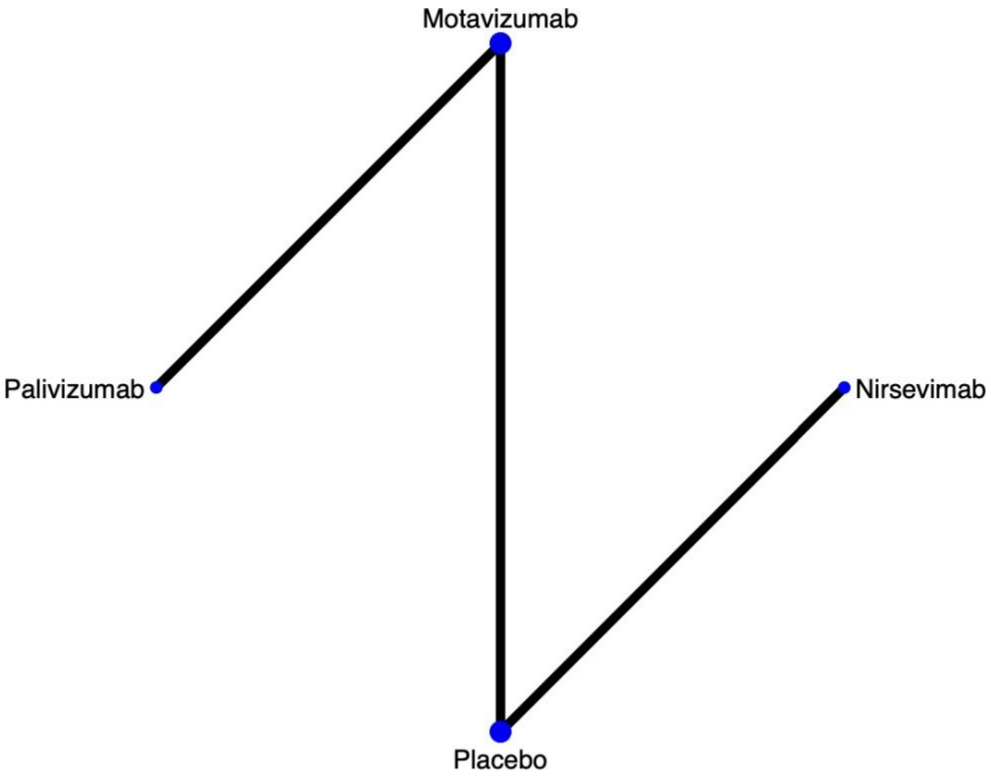

Appendix figure S5: network plot of supplemental oxygen use

© 2023 Sun M et al. *JAMA Network Open*.

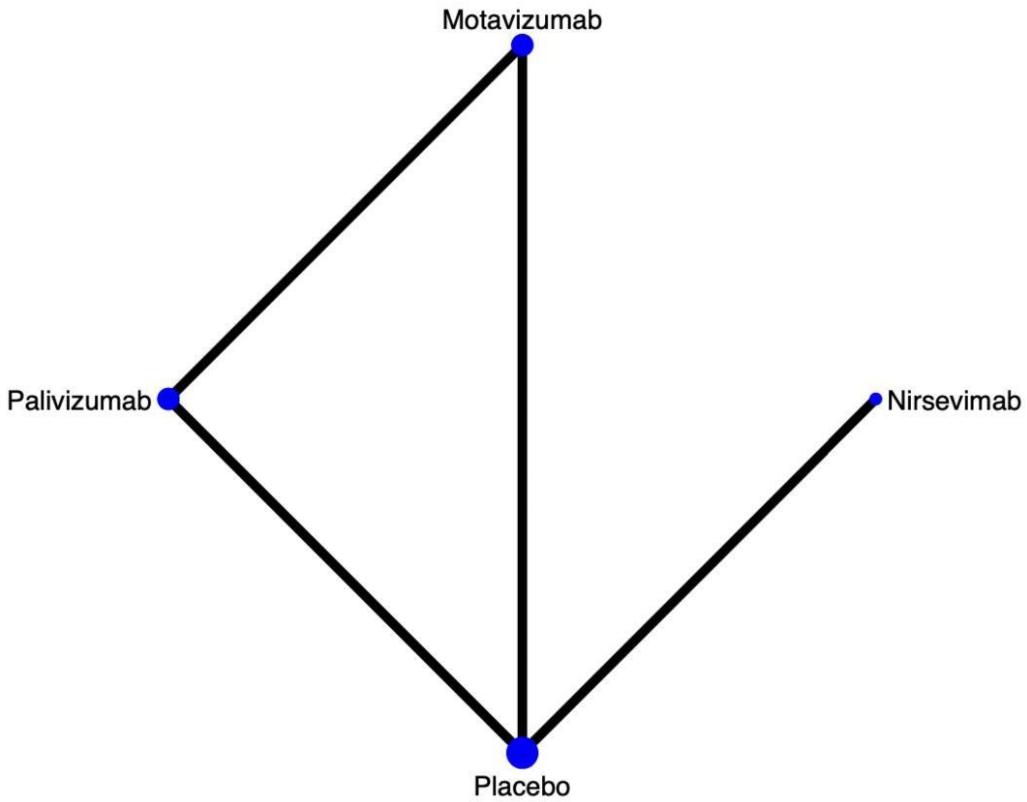

Appendix figure S6: network plot of rate of mechanical ventilation use

© 2023 Sun M et al. *JAMA Network Open*.

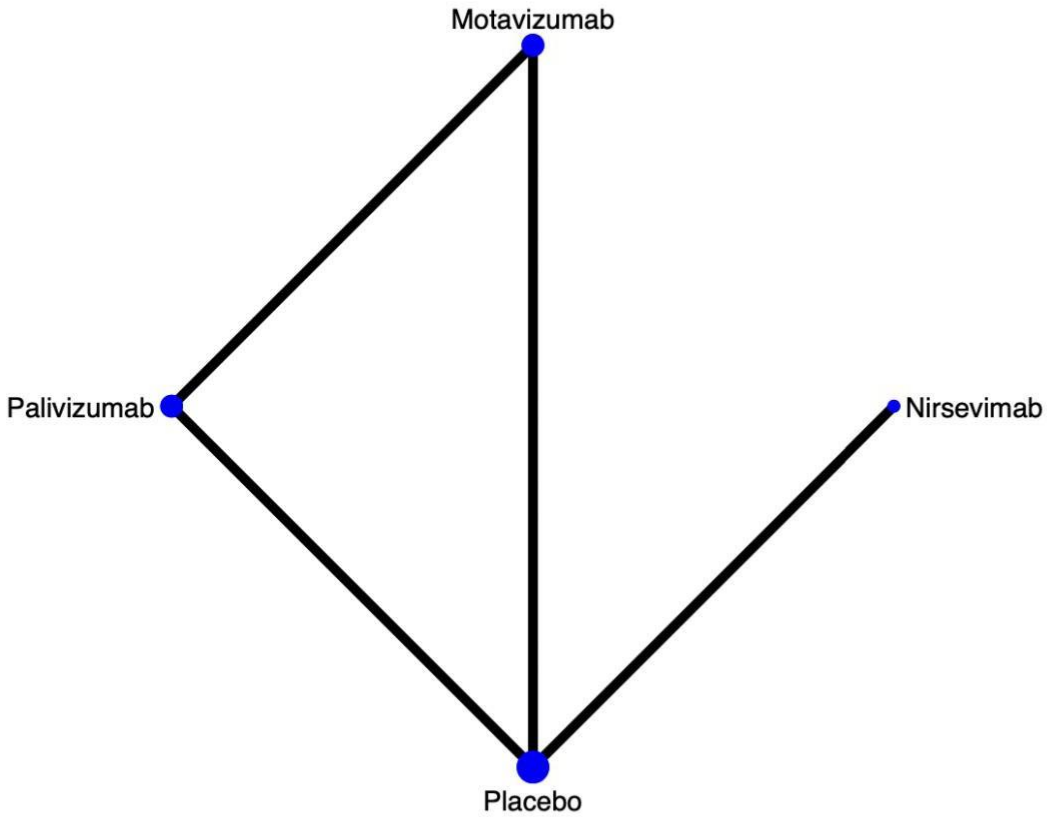

Appendix figure S7: network plot of rate of ICU admission

© 2023 Sun M et al. *JAMA Network Open*.

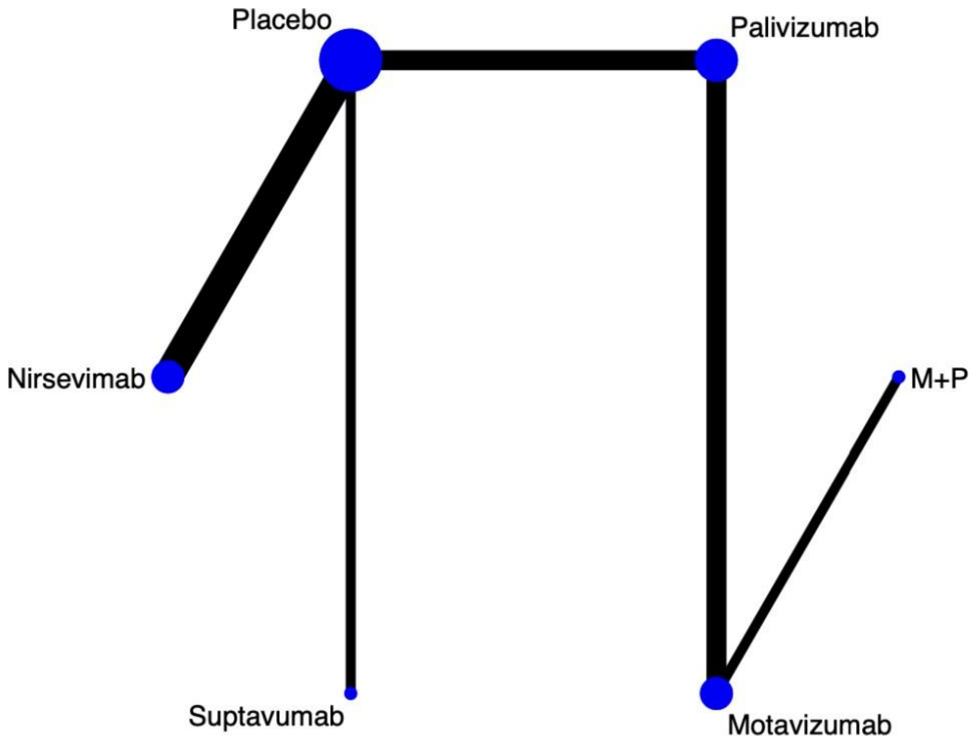

Appendix figure S8: network plot of drug-related adverse events

© 2023 Sun M et al. *JAMA Network Open*.

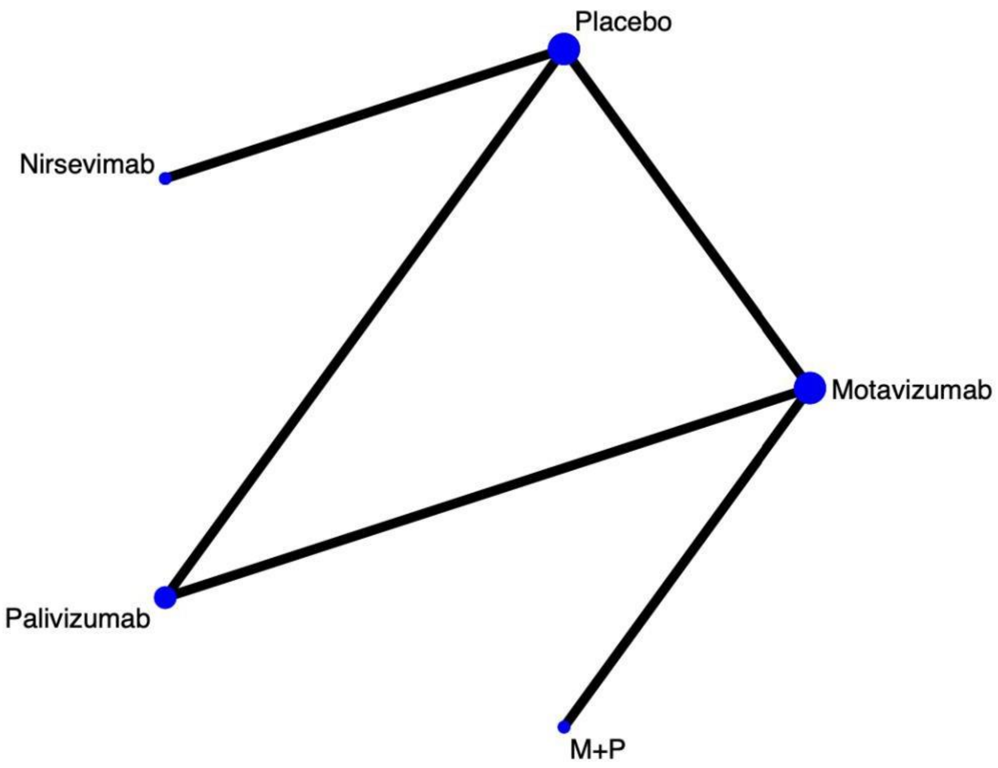

Appendix figure S9: network plot of drug-related serious adverse events

© 2023 Sun M et al. *JAMA Network Open*.

Appendix figure S10-S15: forest plots of all-cause mortality in pairwise meta-analysis

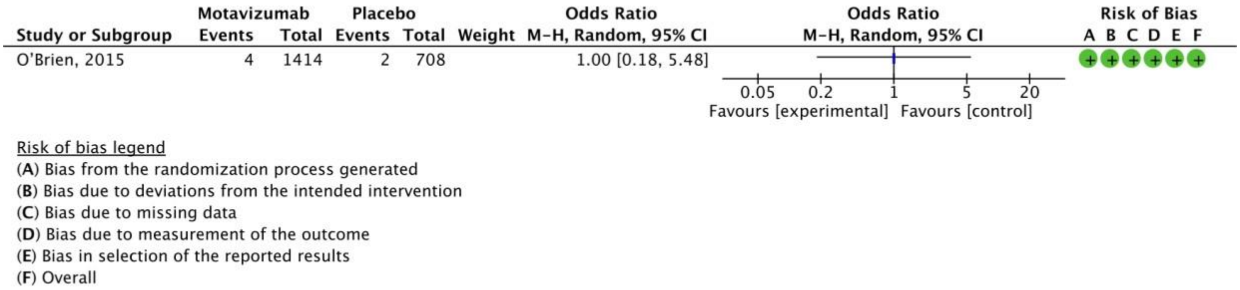

Appendix figure S10: results of motavizumab compared with placebo in all-cause mortality

© 2023 Sun M et al. *JAMA Network Open*.

Study or Subgroup	Nirsevimab Events	Nirsevimab Total	Placebo Events	Placebo Total	Weight	Odds Ratio M–H, Random, 95% CI	Risk of Bias A	B	C	D	E	F
Domachowske, 2018	0	71	0	18		Not estimable	?	+	+	+	+	?
Griffin, 2020	2	968	3	479	62.4%	0.33 [0.05, 1.97]	+	+	+	+	+	+
Hammitt, 2022	3	987	0	491	37.6%	3.49 [0.18, 67.79]	+	+	+	+	+	+
**Total (95% CI)**		**2026**		**988**	**100.0%**	**0.80 [0.08, 7.97]**						
Total events	5		3									

Heterogeneity: $Tau^2 = 1.37$; $Chi^2 = 1.88$, df = 1 (P = 0.17); $I^2 = 47\%$

Test for overall effect: Z = 0.19 (P = 0.85)

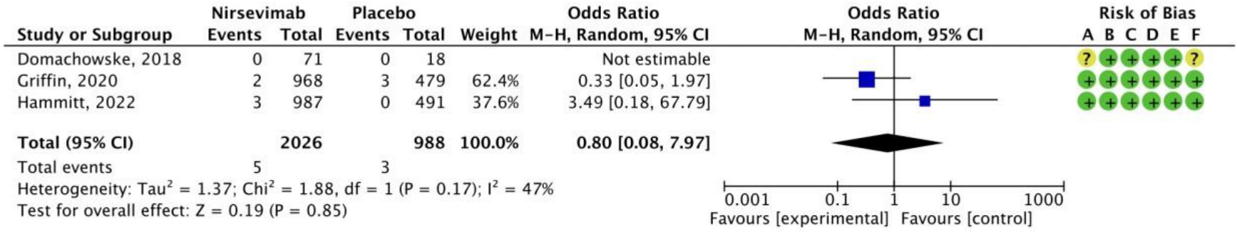

Risk of bias legend

(**A**) Bias from the randomization process generated

(**B**) Bias due to deviations from the intended intervention

(**C**) Bias due to missing data

(**D**) Bias due to measurement of the outcome

(**E**) Bias in selection of the reported results

(**F**) Overall

Appendix figure S11: results of nirsevimab compared with placebo in all-cause mortality

© 2023 Sun M et al. *JAMA Network Open*.

Study or Subgroup	Palivizumab Events	Palivizumab Total	Placebo Events	Placebo Total	Weight	Odds Ratio M–H, Random, 95% CI	A	B	C	D	E	F
Feltes, 2003	21	639	27	648	81.6%	0.78 [0.44, 1.40]	+	+	+	+	+	+
Subramanian, 1998	0	42	1	20	2.6%	0.15 [0.01, 3.93]	+	+	+	+	+	+
The IMpact–RSV Study Group, 1998	4	1002	5	500	15.8%	0.40 [0.11, 1.48]	+	+	+	+	+	+
**Total (95% CI)**		**1683**		**1168**	**100.0%**	**0.67 [0.40, 1.14]**						
Total events	25		33									

Heterogeneity: $Tau^2 = 0.00$; $Chi^2 = 1.67$, df = 2 (P = 0.43); $I^2 = 0\%$

Test for overall effect: Z = 1.48 (P = 0.14)

Odds Ratio M–H, Random, 95% CI

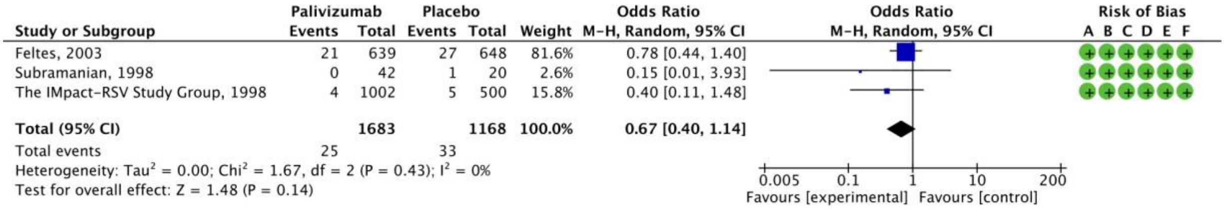

Risk of Bias

Risk of bias legend
(**A**) Bias from the randomization process generated
(**B**) Bias due to deviations from the intended intervention
(**C**) Bias due to missing data
(**D**) Bias due to measurement of the outcome
(**E**) Bias in selection of the reported results
(**F**) Overall

Appendix figure S12: results of palivizumab compared with placebo in all-cause mortality

© 2023 Sun M et al. *JAMA Network Open*.

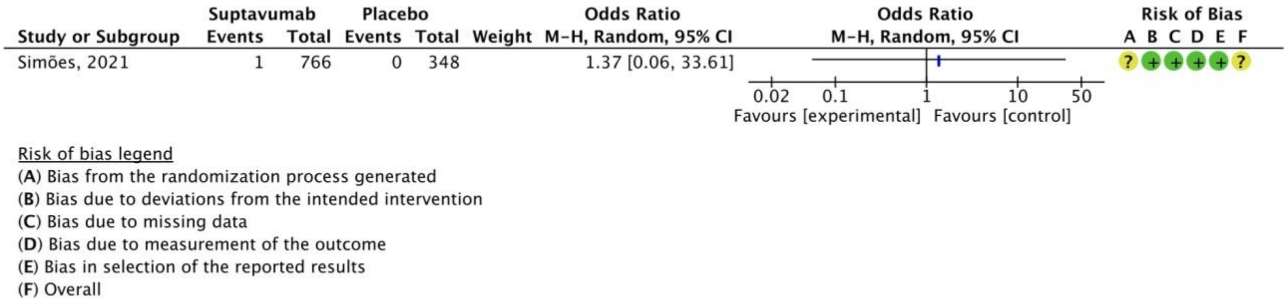

Appendix figure S13: results of suptavumab compared with placebo in all-cause mortality

© 2023 Sun M et al. *JAMA Network Open*.

	Motavizumab		Palivizumab			Odds Ratio	Risk of Bias					
Study or Subgroup	Events	Total	Events	Total	Weight	M–H, Random, 95% CI	A	B	C	D	E	F
Feltes, 2011	9	618	10	612		0.89 [0.36, 2.20]	+	+	+	+	+	+

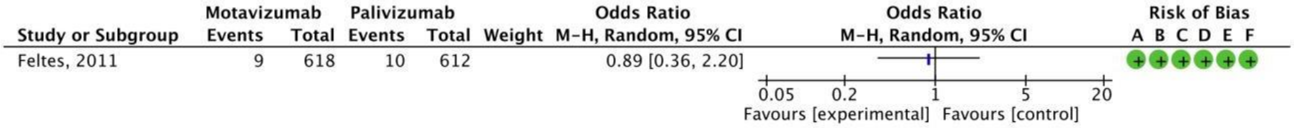

Risk of bias legend
(**A**) Bias from the randomization process generated
(**B**) Bias due to deviations from the intended intervention
(**C**) Bias due to missing data
(**D**) Bias due to measurement of the outcome
(**E**) Bias in selection of the reported results
(**F**) Overall

Appendix figure S14: results of motavizumab compared with palivizumab in all-cause mortality

© 2023 Sun M et al. *JAMA Network Open*.

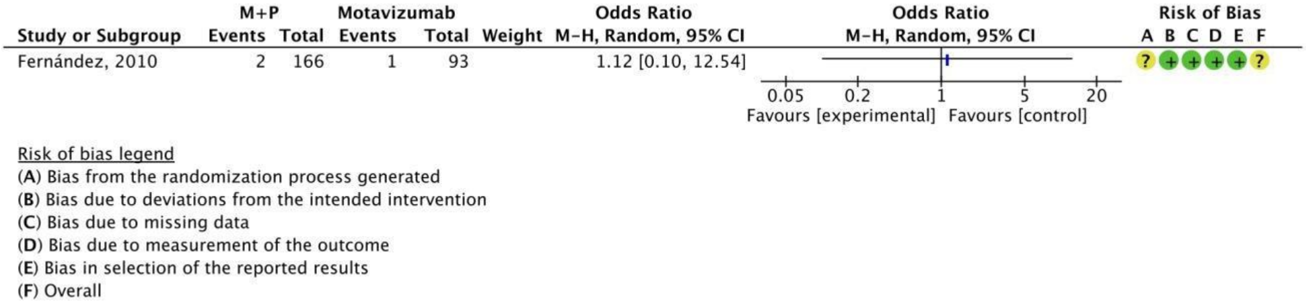

Appendix figure S15: results of motavizumab in combination with palivizumab compared with motavizumab in all-cause mortality

© 2023 Sun M et al. *JAMA Network Open*.

Appendix figure S16-S20: forest plots of drug-related adverse events in pairwise meta-analysis

Study or Subgroup	Nirsevimab Events	Nirsevimab Total	Placebo Events	Placebo Total	Weight	Odds Ratio M-H, Random, 95% CI	A	B	C	D	E	F
Domachowske, 2018	5	71	0	18	4.0%	3.06 [0.16, 57.92]	?	+	+	+	+	?
Griffin, 2020	22	968	10	479	59.9%	1.09 [0.51, 2.32]	+	+	+	+	+	+
Hammitt, 2022	10	987	7	491	36.2%	0.71 [0.27, 1.87]	+	+	+	+	+	+
**Total (95% CI)**		**2026**		**988**	**100.0%**	**0.97 [0.54, 1.74]**						
Total events	37		17									

Heterogeneity: $Tau^2 = 0.00$; $Chi^2 = 1.09$, df = 2 (P = 0.58); $I^2 = 0\%$

Test for overall effect: Z = 0.10 (P = 0.92)

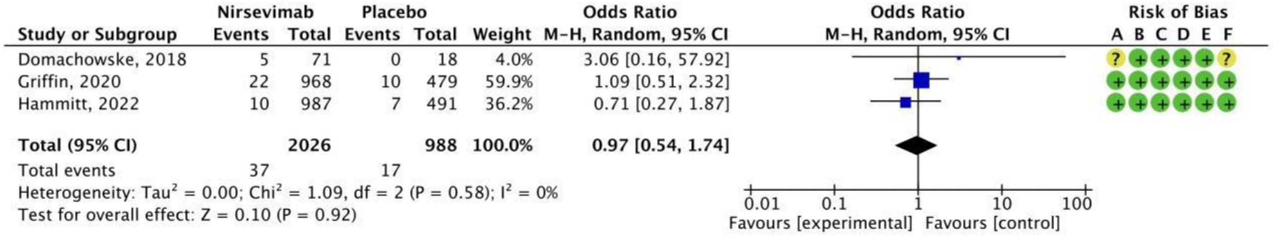

Risk of bias legend

(**A**) Bias from the randomization process generated

(**B**) Bias due to deviations from the intended intervention

(**C**) Bias due to missing data

(**D**) Bias due to measurement of the outcome

(**E**) Bias in selection of the reported results

(**F**) Overall

Appendix figure S16: results of nirsevimab compared with placebo in drug-related adverse events

© 2023 Sun M et al. *JAMA Network Open*.

Study or Subgroup	Palivizumab Events	Palivizumab Total	Placebo Events	Placebo Total	Weight	Odds Ratio M–H, Random, 95% CI
Feltes, 2003	46	639	45	648	92.5%	1.04 [0.68, 1.59]
Subramanian, 1998	6	42	3	20	7.5%	0.94 [0.21, 4.24]
**Total (95% CI)**		**681**		**668**	**100.0%**	**1.03 [0.68, 1.56]**
Total events	52		48			

Heterogeneity: $Tau^2 = 0.00$; $Chi^2 = 0.01$, df = 1 (P = 0.90); $I^2 = 0\%$
Test for overall effect: Z = 0.15 (P = 0.88)

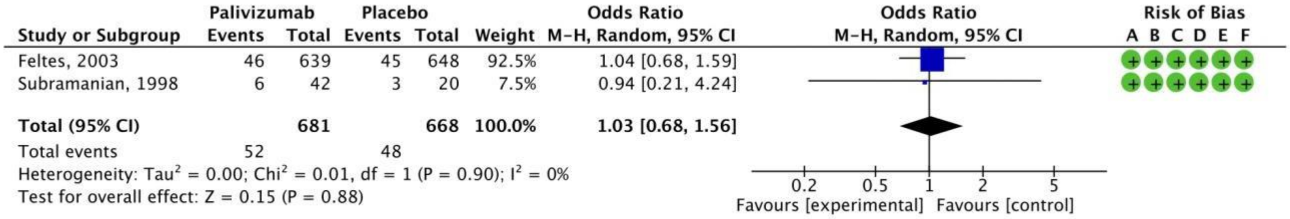

Risk of bias legend
(**A**) Bias from the randomization process generated
(**B**) Bias due to deviations from the intended intervention
(**C**) Bias due to missing data
(**D**) Bias due to measurement of the outcome
(**E**) Bias in selection of the reported results
(**F**) Overall

Appendix figure S17: results of palivizumab compared with placebo in drug-related adverse events

© 2023 Sun M et al. *JAMA Network Open*.

Study or Subgroup	Suptavumab Events	Suptavumab Total	Placebo Events	Placebo Total	Weight	Odds Ratio M-H, Random, 95% CI	Odds Ratio M-H, Random, 95% CI	Risk of Bias A	B	C	D	E	F
Simões, 2021	51	766	23	348		1.01 [0.61, 1.68]	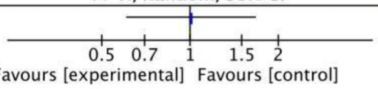	?	+	+	+	+	?

Risk of bias legend
(**A**) Bias from the randomization process generated
(**B**) Bias due to deviations from the intended intervention
(**C**) Bias due to missing data
(**D**) Bias due to measurement of the outcome
(**E**) Bias in selection of the reported results
(**F**) Overall

Appendix figure S18: results of suptavumab compared with placebo in drug-related adverse events

© 2023 Sun M et al. *JAMA Network Open*.

Study or Subgroup	Motavizumab Events	Motavizumab Total	Palivizumab Events	Palivizumab Total	Weight	Odds Ratio M-H, Random, 95% CI
Carbonell-Estrany, 2010	13	3315	10	3298	19.0%	1.29 [0.57, 2.96]
Feltes, 2011	51	618	54	612	81.0%	0.93 [0.62, 1.39]
**Total (95% CI)**		**3933**		**3910**	**100.0%**	**0.99 [0.69, 1.42]**
Total events	64		64			

Heterogeneity: Tau^2 = 0.00; Chi^2 = 0.50, df = 1 (P = 0.48); I^2 = 0%

Test for overall effect: Z = 0.06 (P = 0.96)

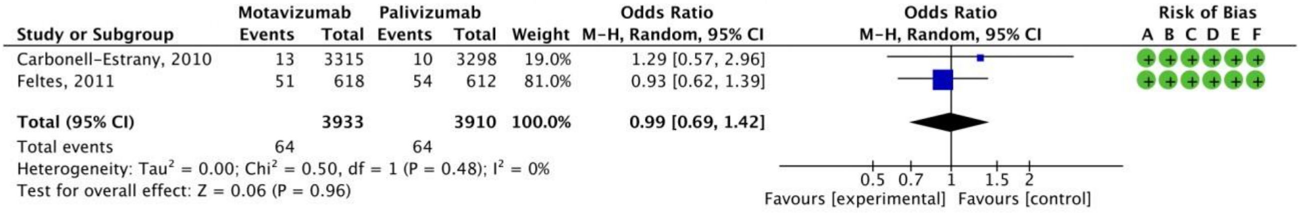

Risk of bias legend

(**A**) Bias from the randomization process generated

(**B**) Bias due to deviations from the intended intervention

(**C**) Bias due to missing data

(**D**) Bias due to measurement of the outcome

(**E**) Bias in selection of the reported results

(**F**) Overall

Appendix figure S19: results of motavizumab compared with palivizumab in drug-related adverse events

© 2023 Sun M et al. *JAMA Network Open*.

Study or Subgroup	M+P Events	M+P Total	Motavizumab Events	Motavizumab Total	Weight	Odds Ratio M-H, Random, 95% CI	Odds Ratio M-H, Random, 95% CI	Risk of Bias A	B	C	D	E	F
Fernández, 2010	30	166	21	93		0.76 [0.40, 1.42]		?	+	+	+	+	?

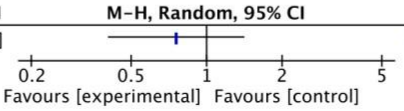

Risk of bias legend
(**A**) Bias from the randomization process generated
(**B**) Bias due to deviations from the intended intervention
(**C**) Bias due to missing data
(**D**) Bias due to measurement of the outcome
(**E**) Bias in selection of the reported results
(**F**) Overall

Appendix figure S20: results of motavizumab in combination with palivizumab compared with motavizumab in drug-related adverse events

© 2023 Sun M et al. *JAMA Network Open*.

Appendix figure S21-S25: forest plots of drug-related serious adverse events in pairwise meta-analysis

Study or Subgroup	Motavizumab Events	Motavizumab Total	Placebo Events	Placebo Total	Weight	Odds Ratio M–H, Random, 95% CI	Risk of Bias A	B	C	D	E	F
O'Brien, 2015	8	1414	0	708		8.56 [0.49, 148.58]	+	+	+	+	+	+

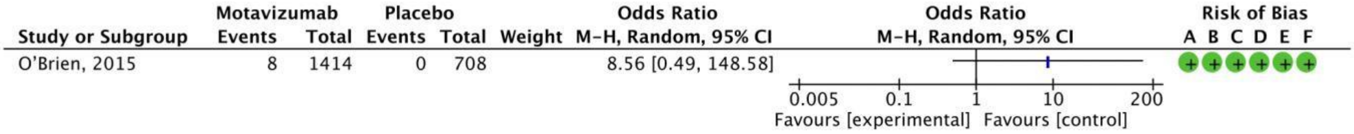

Risk of bias legend
(**A**) Bias from the randomization process generated
(**B**) Bias due to deviations from the intended intervention
(**C**) Bias due to missing data
(**D**) Bias due to measurement of the outcome
(**E**) Bias in selection of the reported results
(**F**) Overall

Appendix figure S21: results of motavizumab compared with placebo in drug-related serious adverse events

© 2023 Sun M et al. *JAMA Network Open*.

Study or Subgroup	Nirsevimab Events	Nirsevimab Total	Placebo Events	Placebo Total	Weight	Odds Ratio M-H, Random, 95% CI	Odds Ratio M-H, Random, 95% CI	Risk of Bias A	B	C	D	E	F
Griffin, 2020	0	968	0	479		Not estimable		+	+	+	+	+	+

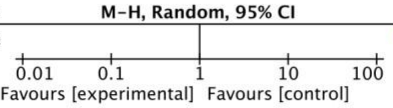

Risk of bias legend
(**A**) Bias from the randomization process generated
(**B**) Bias due to deviations from the intended intervention
(**C**) Bias due to missing data
(**D**) Bias due to measurement of the outcome
(**E**) Bias in selection of the reported results
(**F**) Overall

Appendix figure S22: results of nirsevimab compared with placebo in drug-related serious adverse events

© 2023 Sun M et al. *JAMA Network Open*.

Study or Subgroup	Palivizumab Events	Palivizumab Total	Placebo Events	Placebo Total	Weight	Odds Ratio M–H, Random, 95% CI	Risk of Bias A	B	C	D	E	F
Feltes, 2003	0	639	3	648		0.14 [0.01, 2.80]	+	+	+	+	+	+

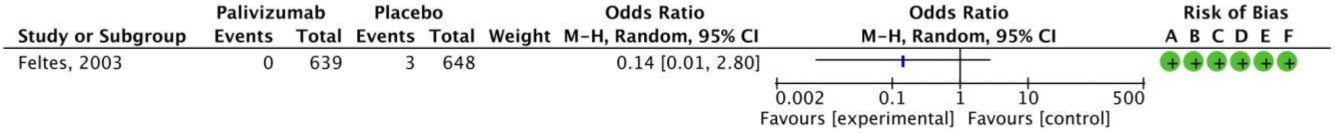

Risk of bias legend
(**A**) Bias from the randomization process generated
(**B**) Bias due to deviations from the intended intervention
(**C**) Bias due to missing data
(**D**) Bias due to measurement of the outcome
(**E**) Bias in selection of the reported results
(**F**) Overall

Appendix figure S23: results of palivizumab compared with placebo in drug-related serious adverse events

© 2023 Sun M et al. *JAMA Network Open*.

Study or Subgroup	Motavizumab Events	Motavizumab Total	Palivizumab Events	Palivizumab Total	Weight	Odds Ratio M-H, Random, 95% CI	Risk of Bias A	B	C	D	E	F
Feltes, 2011	5	618	6	612		0.82 [0.25, 2.71]	+	+	+	+	+	+

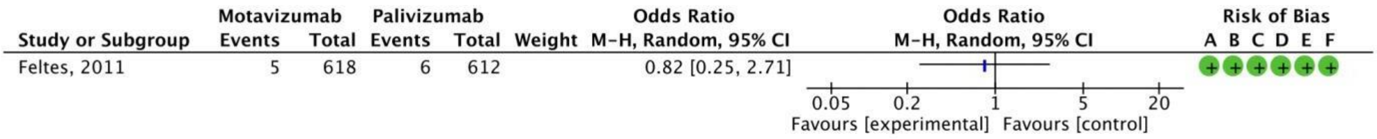

Risk of bias legend
(**A**) Bias from the randomization process generated
(**B**) Bias due to deviations from the intended intervention
(**C**) Bias due to missing data
(**D**) Bias due to measurement of the outcome
(**E**) Bias in selection of the reported results
(**F**) Overall

Appendix figure S24: results of motavizumab compared with palivizumab in drug-related serious adverse events

© 2023 Sun M et al. *JAMA Network Open*.

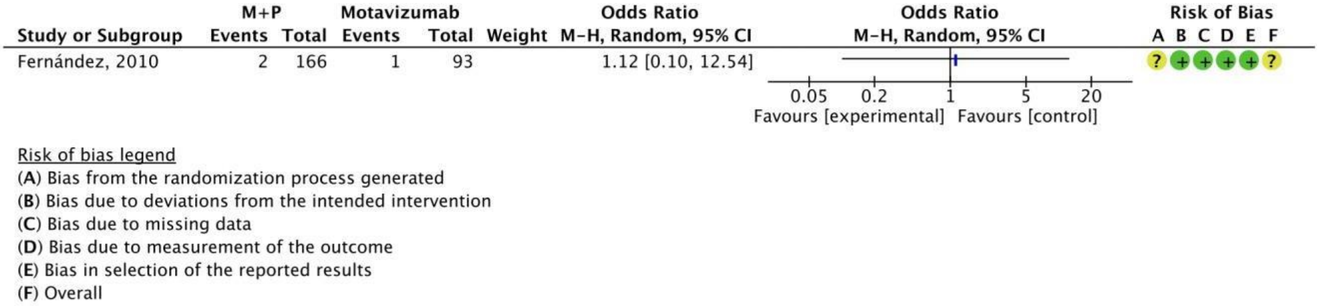

Appendix figure S25: results of motavizumab in combination with palivizumab compared with motavizumab in drug-related serious adverse events

© 2023 Sun M et al. *JAMA Network Open*.

Appendix figure S26-S29: forest plots of rate of RSV-related hospitalization in pairwise meta-analysis

Study or Subgroup	Nirsevimab Events	Nirsevimab Total	Placebo Events	Placebo Total	Weight	Odds Ratio M–H, Random, 95% CI	Risk of Bias A B C D E F
Griffin, 2020	8	969	20	484	62.3%	0.19 [0.08, 0.44]	+ + + + + +
Hammitt, 2022	6	994	8	496	37.7%	0.37 [0.13, 1.07]	+ + + + + +
**Total (95% CI)**		**1963**		**980**	**100.0%**	**0.25 [0.13, 0.47]**	
Total events	14		28				

Heterogeneity: $Tau^2 = 0.00$; $Chi^2 = 0.90$, df = 1 (P = 0.34); $I^2 = 0\%$
Test for overall effect: Z = 4.20 (P < 0.0001)

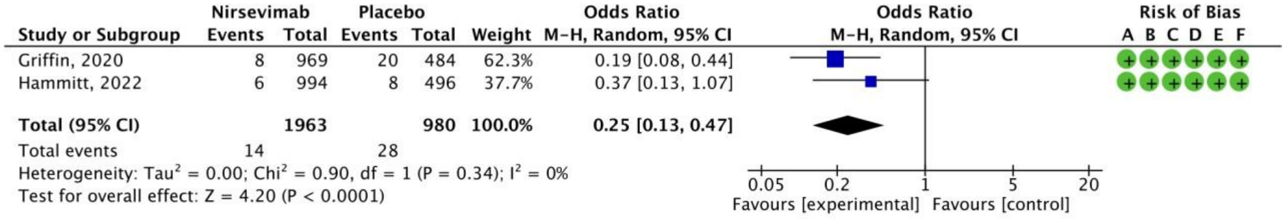

Risk of bias legend
(**A**) Bias from the randomization process generated
(**B**) Bias due to deviations from the intended intervention
(**C**) Bias due to missing data
(**D**) Bias due to measurement of the outcome
(**E**) Bias in selection of the reported results
(**F**) Overall

Appendix figure S26: results of nirsevimab compared with placebo in RSV-related hospitalization

© 2023 Sun M et al. *JAMA Network Open*.

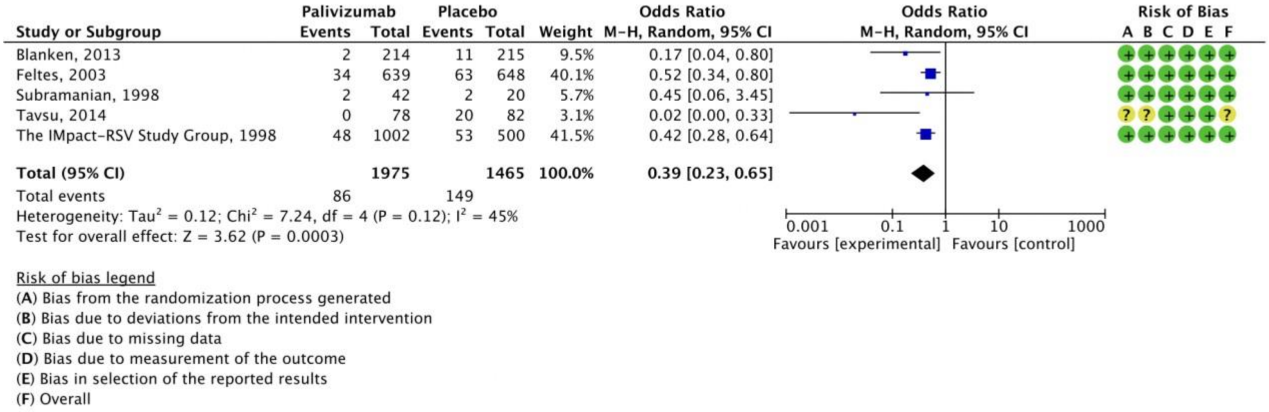

Appendix figure S27: results of palivizumab compared with placebo in RSV-related hospitalization

© 2023 Sun M et al. *JAMA Network Open*.

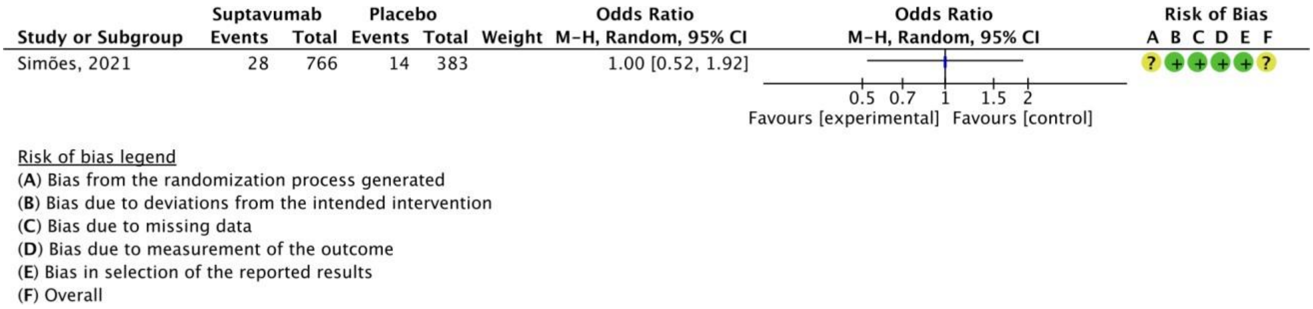

Appendix figure S28: results of suptavumab compared with placebo in RSV-related hospitalization

© 2023 Sun M et al. *JAMA Network Open*.

	Motavizumab		Palivizumab			Odds Ratio	Risk of Bias					
Study or Subgroup	Events	Total	Events	Total	Weight	M–H, Random, 95% CI	A	B	C	D	E	F
Carbonell–Estrany, 2010	46	3329	62	3306		0.73 [0.50, 1.08]	+	+	+	+	+	+

Odds Ratio
M–H, Random, 95% CI

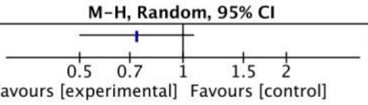

<u>Risk of bias legend</u>
(**A**) Bias from the randomization process generated
(**B**) Bias due to deviations from the intended intervention
(**C**) Bias due to missing data
(**D**) Bias due to measurement of the outcome
(**E**) Bias in selection of the reported results
(**F**) Overall

Appendix figure S29: results of motavizumab compared with palivizumab in RSV-related hospitalization

© 2023 Sun M et al. *JAMA Network Open*.

Appendix figure S30-S33: forest plots of rate of RSV infection in pairwise meta-analysis

Study or Subgroup	Motavizumab Events	Motavizumab Total	Placebo Events	Placebo Total	Weight	Odds Ratio M-H, Fixed, 95% CI	Risk of Bias A	B	C	D	E	F
O'Brien, 2015	61	1417	151	710		0.17 [0.12, 0.23]	+	+	+	+	+	+

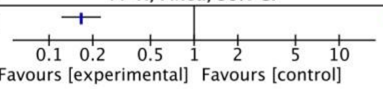

Risk of bias legend
(**A**) Bias from the randomization process generated
(**B**) Bias due to deviations from the intended intervention
(**C**) Bias due to missing data
(**D**) Bias due to measurement of the outcome
(**E**) Bias in selection of the reported results
(**F**) Overall

Appendix figure S30: results of motavizumab compared with placebo in RSV infection

© 2023 Sun M et al. *JAMA Network Open*.

Study or Subgroup	Nirsevimab Events	Nirsevimab Total	Placebo Events	Placebo Total	Weight	Odds Ratio M-H, Fixed, 95% CI
Griffin, 2020	25	969	46	484	64.5%	0.25 [0.15, 0.42]
Hammitt, 2022	12	994	25	496	35.5%	0.23 [0.11, 0.46]
**Total (95% CI)**		**1963**		**980**	**100.0%**	**0.24 [0.16, 0.37]**
Total events	37		71			

Heterogeneity: $Chi^2 = 0.04$, df = 1 (P = 0.84); $I^2 = 0\%$

Test for overall effect: Z = 6.80 (P < 0.00001)

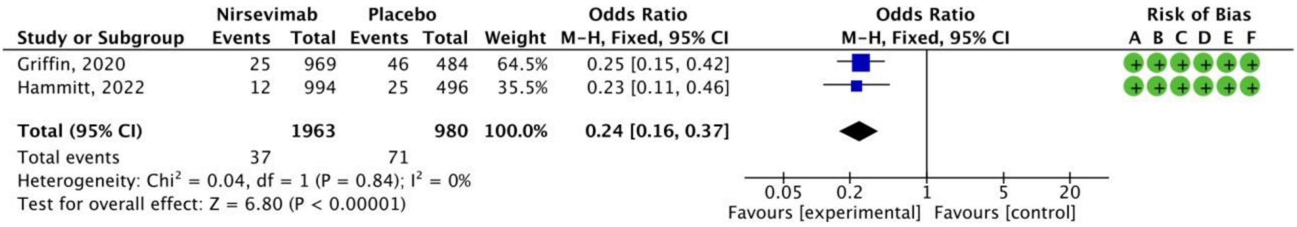

Risk of bias legend
(**A**) Bias from the randomization process generated
(**B**) Bias due to deviations from the intended intervention
(**C**) Bias due to missing data
(**D**) Bias due to measurement of the outcome
(**E**) Bias in selection of the reported results
(**F**) Overall

Appendix figure S31: results of nirsevimab compared with placebo in RSV infection

© 2023 Sun M et al. *JAMA Network Open*.

Study or Subgroup	Palivizumab Events	Palivizumab Total	Placebo Events	Placebo Total	Weight	Odds Ratio M-H, Fixed, 95% CI	Risk of Bias A	B	C	D	E	F
Blanken, 2013	10	214	30	215	29.4%	0.30 [0.14, 0.64]	+	+	+	+	+	+
Subramanian, 1998	6	42	4	20	4.8%	0.67 [0.17, 2.69]	+	+	+	+	+	+
Tavsu, 2014	40	156	88	164	65.8%	0.30 [0.19, 0.48]	?	?	+	+	+	?
**Total (95% CI)**		**412**		**399**	**100.0%**	**0.32 [0.22, 0.46]**						
Total events	56		122									

Heterogeneity: Chi^2 = 1.17, df = 2 (P = 0.56); I^2 = 0%
Test for overall effect: Z = 5.90 (P < 0.00001)

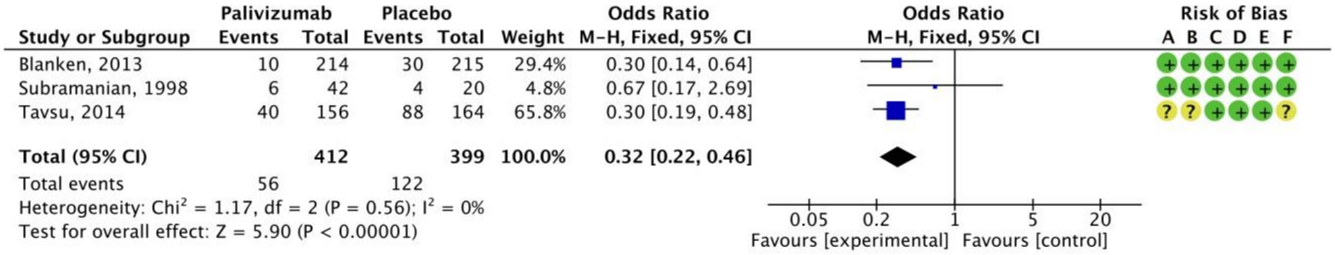

Risk of bias legend
(**A**) Bias from the randomization process generated
(**B**) Bias due to deviations from the intended intervention
(**C**) Bias due to missing data
(**D**) Bias due to measurement of the outcome
(**E**) Bias in selection of the reported results
(**F**) Overall

Appendix figure S32: results of palivizumab compared with placebo in RSV infection

© 2023 Sun M et al. *JAMA Network Open*.

Study or Subgroup	Suptavumab Events	Suptavumab Total	Placebo Events	Placebo Total	Weight	Odds Ratio M–H, Fixed, 95% CI	Risk of Bias A	B	C	D	E	F
Simões, 2021	101	766	48	383		1.06 [0.73, 1.53]	?	+	+	+	+	?

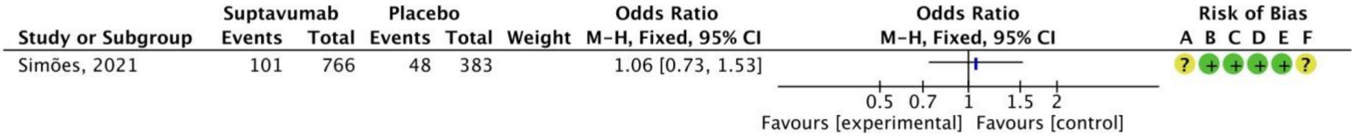

Risk of bias legend
(**A**) Bias from the randomization process generated
(**B**) Bias due to deviations from the intended intervention
(**C**) Bias due to missing data
(**D**) Bias due to measurement of the outcome
(**E**) Bias in selection of the reported results
(**F**) Overall

Appendix figure S33: results of suptavumab compared with placebo in RSV infection

© 2023 Sun M et al. *JAMA Network Open*.

Appendix figure S34-S36: forest plots of rate of supplemental oxygen use in pairwise meta-analysis

Study or Subgroup	Motavizumab Events	Motavizumab Total	Placebo Events	Placebo Total	Weight	Odds Ratio M–H, Random, 95% CI	Risk of Bias A	B	C	D	E	F
O'Brien, 2015	19	1417	75	710		0.12 [0.07, 0.19]	+	+	+	+	+	+

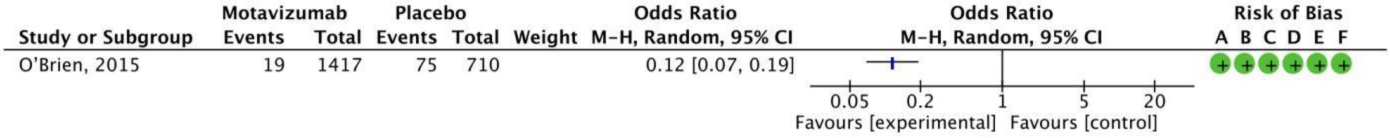

Risk of bias legend
(**A**) Bias from the randomization process generated
(**B**) Bias due to deviations from the intended intervention
(**C**) Bias due to missing data
(**D**) Bias due to measurement of the outcome
(**E**) Bias in selection of the reported results
(**F**) Overall

Appendix figure S34: results of motavizumab compared with placebo in supplemental oxygen use

© 2023 Sun M et al. *JAMA Network Open*.

	Nirsevimab		Placebo			Odds Ratio	Odds Ratio	Risk of Bias					
Study or Subgroup	Events	Total	Events	Total	Weight	M–H, Random, 95% CI	M–H, Random, 95% CI	A	B	C	D	E	F
Griffin, 2020	4	968	15	479		0.13 [0.04, 0.39]		+	+	+	+	+	+

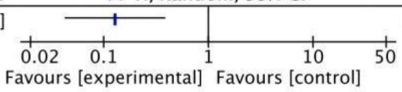

<u>Risk of bias legend</u>
(**A**) Bias from the randomization process generated
(**B**) Bias due to deviations from the intended intervention
(**C**) Bias due to missing data
(**D**) Bias due to measurement of the outcome
(**E**) Bias in selection of the reported results
(**F**) Overall

Appendix figure S35: results of nirsevimab compared with placebo in supplemental oxygen use

© 2023 Sun M et al. *JAMA Network Open*.

Study or Subgroup	Motavizumab Events	Motavizumab Total	Palivizumab Events	Palivizumab Total	Weight	Odds Ratio M-H, Random, 95% CI	Odds Ratio M-H, Random, 95% CI	Risk of Bias A B C D E F
Carbonell-Estrany, 2010	26	3329	40	3306		0.64 [0.39, 1.06]		+ + + + + +

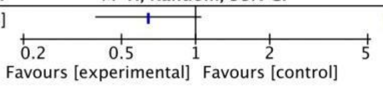

Risk of bias legend
(**A**) Bias from the randomization process generated
(**B**) Bias due to deviations from the intended intervention
(**C**) Bias due to missing data
(**D**) Bias due to measurement of the outcome
(**E**) Bias in selection of the reported results
(**F**) Overall

Appendix figure S36: results of motavizumab compared with palivizumab in supplemental oxygen use

© 2023 Sun M et al. *JAMA Network Open*.

Appendix figure S37-S40: forest plots of rate of mechanical ventilation use in pairwise meta-analysis

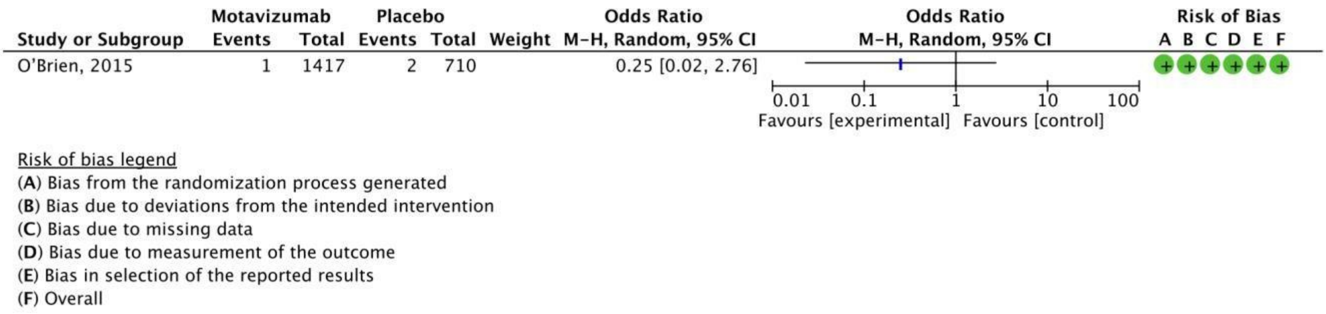

Appendix figure S37: results of motavizumab compared with placebo in mechanical ventilation use

© 2023 Sun M et al. *JAMA Network Open*.

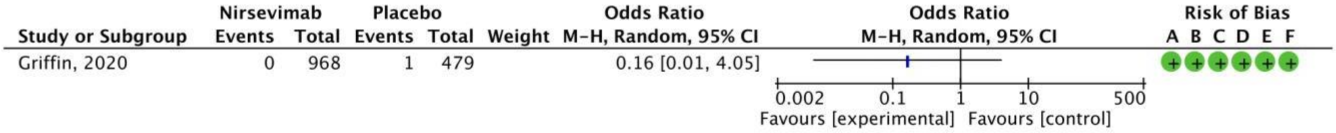

Risk of bias legend
(**A**) Bias from the randomization process generated
(**B**) Bias due to deviations from the intended intervention
(**C**) Bias due to missing data
(**D**) Bias due to measurement of the outcome
(**E**) Bias in selection of the reported results
(**F**) Overall

Appendix figure S38: results of nirsevimab compared with placebo in mechanical ventilation use

© 2023 Sun M et al. *JAMA Network Open*.

Study or Subgroup	Palivizumab Events	Palivizumab Total	Placebo Events	Placebo Total	Weight	Odds Ratio M-H, Random, 95% CI	Risk of Bias A	B	C	D	E	F
Feltes, 2003	8	639	14	648		0.57 [0.24, 1.38]	+	+	+	+	+	+

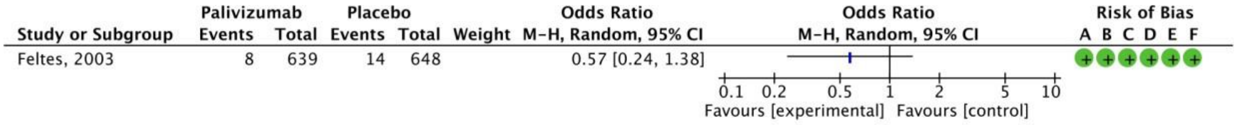

Risk of bias legend
(**A**) Bias from the randomization process generated
(**B**) Bias due to deviations from the intended intervention
(**C**) Bias due to missing data
(**D**) Bias due to measurement of the outcome
(**E**) Bias in selection of the reported results
(**F**) Overall

Appendix figure S39: results of palivizumab compared with placebo in mechanical ventilation use

© 2023 Sun M et al. *JAMA Network Open*.

Study or Subgroup	Motavizumab Events	Motavizumab Total	Palivizumab Events	Palivizumab Total	Weight	Odds Ratio M–H, Random, 95% CI	Risk of Bias A	B	C	D	E	F
Carbonell–Estrany, 2010	2	3329	11	3306		0.18 [0.04, 0.81]	+	+	+	+	+	+

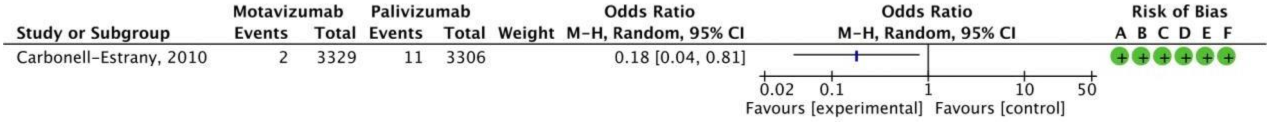

Risk of bias legend
(**A**) Bias from the randomization process generated
(**B**) Bias due to deviations from the intended intervention
(**C**) Bias due to missing data
(**D**) Bias due to measurement of the outcome
(**E**) Bias in selection of the reported results
(**F**) Overall

Appendix figure S40: results of motavizumab compared with palivizumab in mechanical ventilation use

© 2023 Sun M et al. *JAMA Network Open*.

Appendix figure S41-S44: forest plots of rate of ICU admission in pairwise meta-analysis

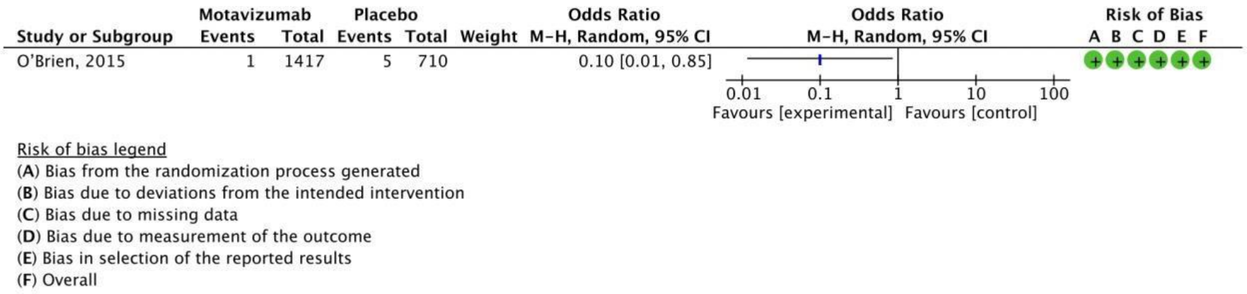

Appendix figure S41: results of motavizumab compared with placebo in ICU admission

© 2023 Sun M et al. *JAMA Network Open*.

Study or Subgroup	Nirsevimab Events	Nirsevimab Total	Placebo Events	Placebo Total	Weight	Odds Ratio M-H, Random, 95% CI	Risk of Bias A	B	C	D	E	F
Griffin, 2020	0	968	5	479		0.04 [0.00, 0.81]	+	+	+	+	+	+

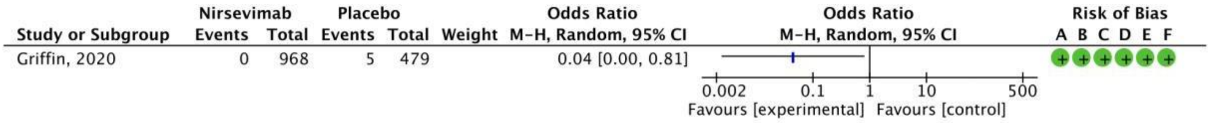

Risk of bias legend
(**A**) Bias from the randomization process generated
(**B**) Bias due to deviations from the intended intervention
(**C**) Bias due to missing data
(**D**) Bias due to measurement of the outcome
(**E**) Bias in selection of the reported results
(**F**) Overall

Appendix figure S42: results of nirsevimab compared with placebo in ICU admission

© 2023 Sun M et al. *JAMA Network Open*.

Study or Subgroup	Palivizumab Events	Palivizumab Total	Placebo Events	Placebo Total	Weight	Odds Ratio M-H, Random, 95% CI	Odds Ratio M-H, Random, 95% CI	Risk of Bias A	B	C	D	E	F
Feltes, 2003	13	639	24	648		0.54 [0.27, 1.07]	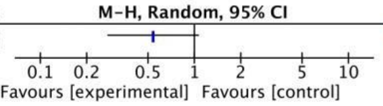	+	+	+	+	+	+

Risk of bias legend
(**A**) Bias from the randomization process generated
(**B**) Bias due to deviations from the intended intervention
(**C**) Bias due to missing data
(**D**) Bias due to measurement of the outcome
(**E**) Bias in selection of the reported results
(**F**) Overall

Appendix figure S43: results of palivizumab compared with placebo in ICU admission

© 2023 Sun M et al. *JAMA Network Open*.

Study or Subgroup	Motavizumab Events	Motavizumab Total	Palivizumab Events	Palivizumab Total	Weight	Odds Ratio M-H, Random, 95% CI	Odds Ratio M-H, Random, 95% CI	Risk of Bias A	B	C	D	E	F
Carbonell-Estrany, 2010	10	3329	19	3306		0.52 [0.24, 1.12]		+	+	+	+	+	+

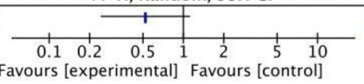

Risk of bias legend
(**A**) Bias from the randomization process generated
(**B**) Bias due to deviations from the intended intervention
(**C**) Bias due to missing data
(**D**) Bias due to measurement of the outcome
(**E**) Bias in selection of the reported results
(**F**) Overall

Appendix figure S44: results of motavizumab compared with palivizumab in ICU admission

© 2023 Sun M et al. *JAMA Network Open*.

Appendix figure S45: funnel plot of all-cause mortality

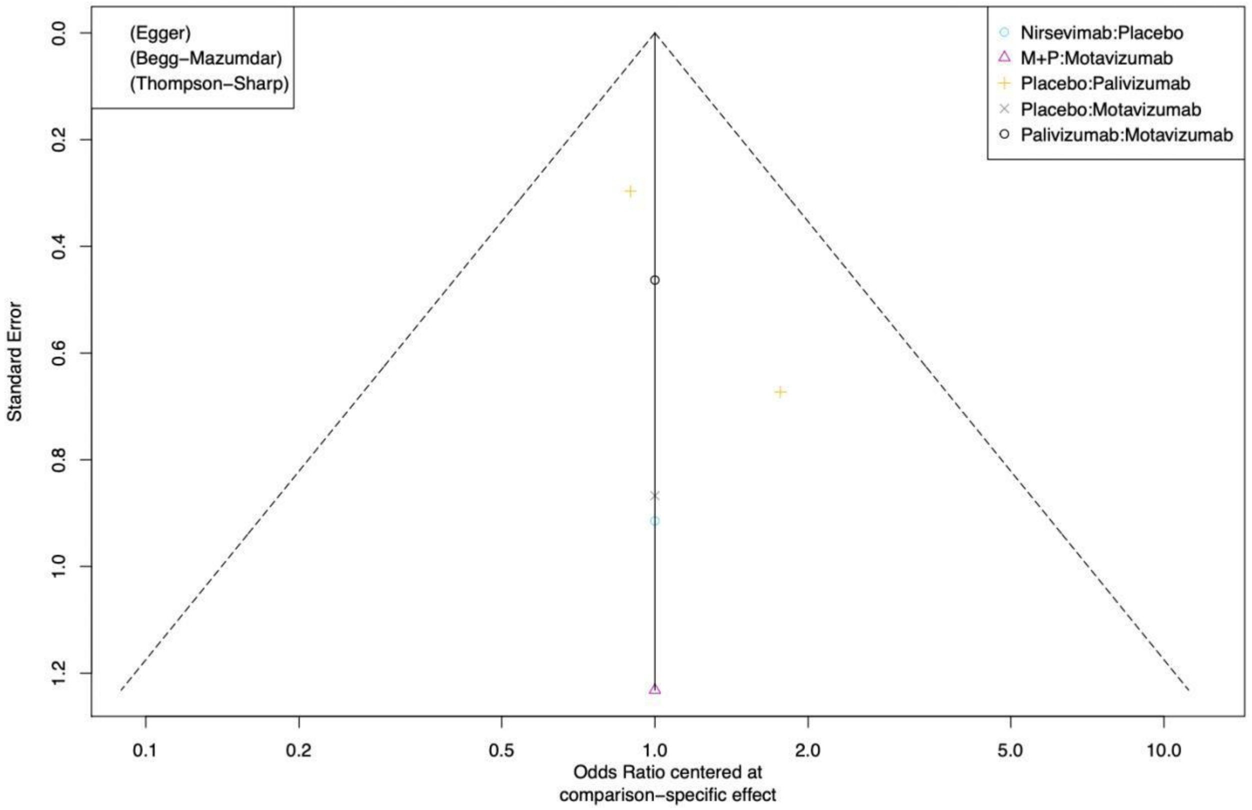

Appendix figure S39: funnel plot of all-cause mortality

© 2023 Sun M et al. *JAMA Network Open*.

Appendix figure S46-S59: node-split plots

The number of the direct evidence column is the P-value to test for differences between groups.

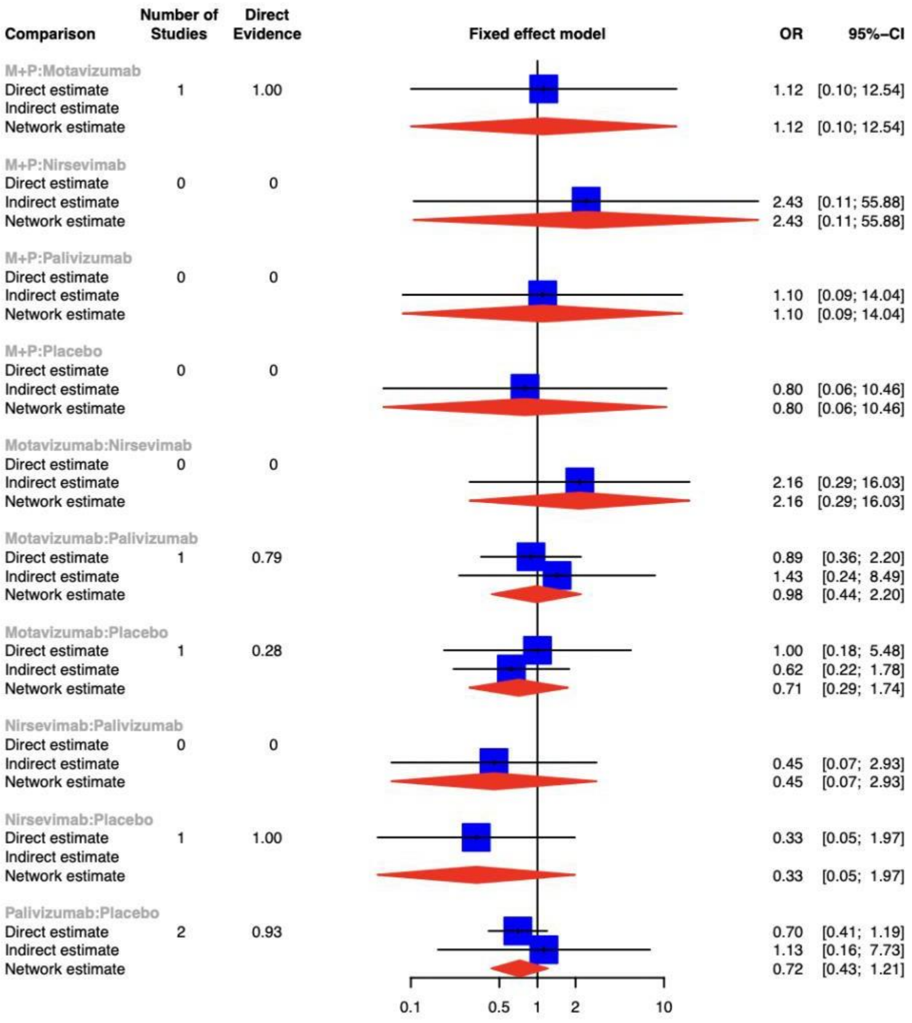

Appendix figure S46: node-split plot of all-cause mortality in fixed effects model

© 2023 Sun M et al. *JAMA Network Open*.

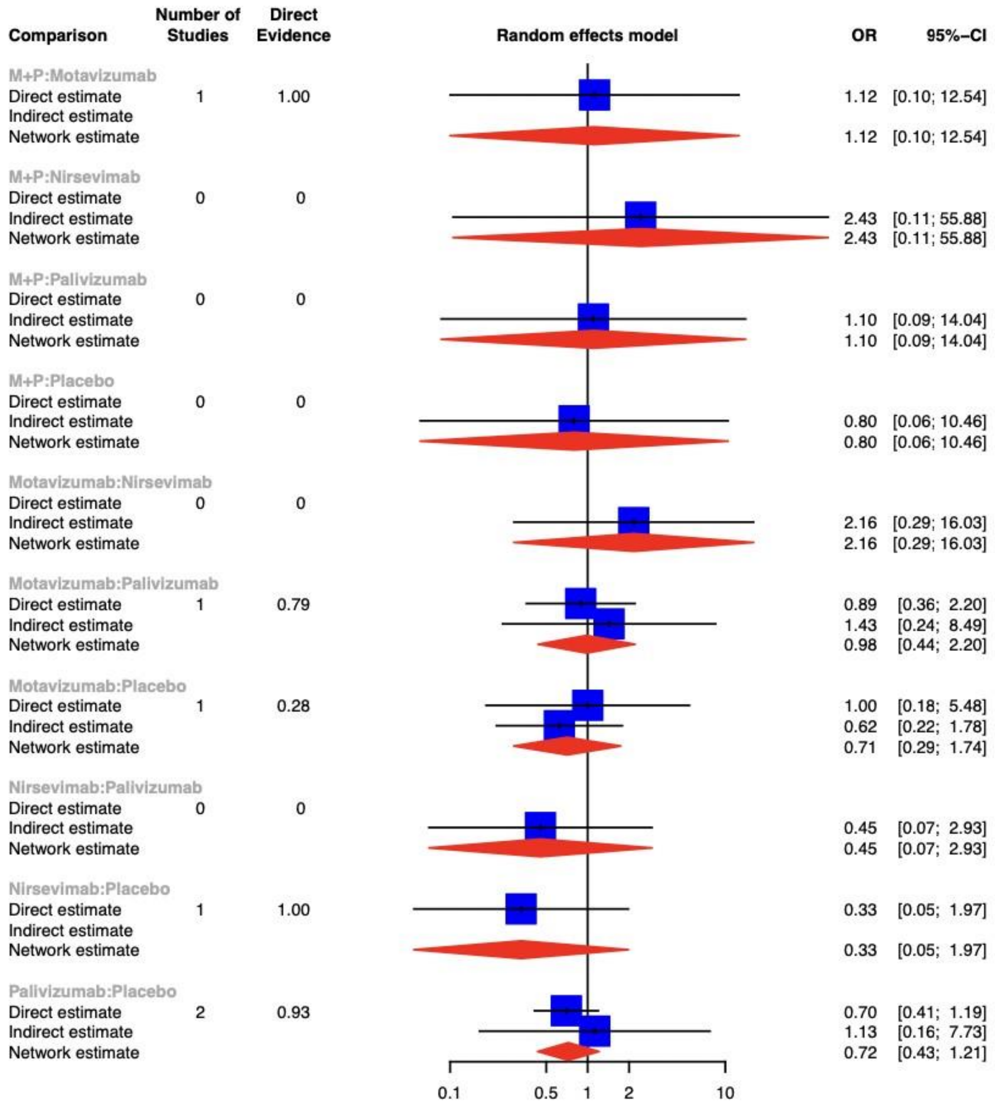

Appendix figure S47: node-split plot of all-cause mortality in random effects model

© 2023 Sun M et al. *JAMA Network Open*.

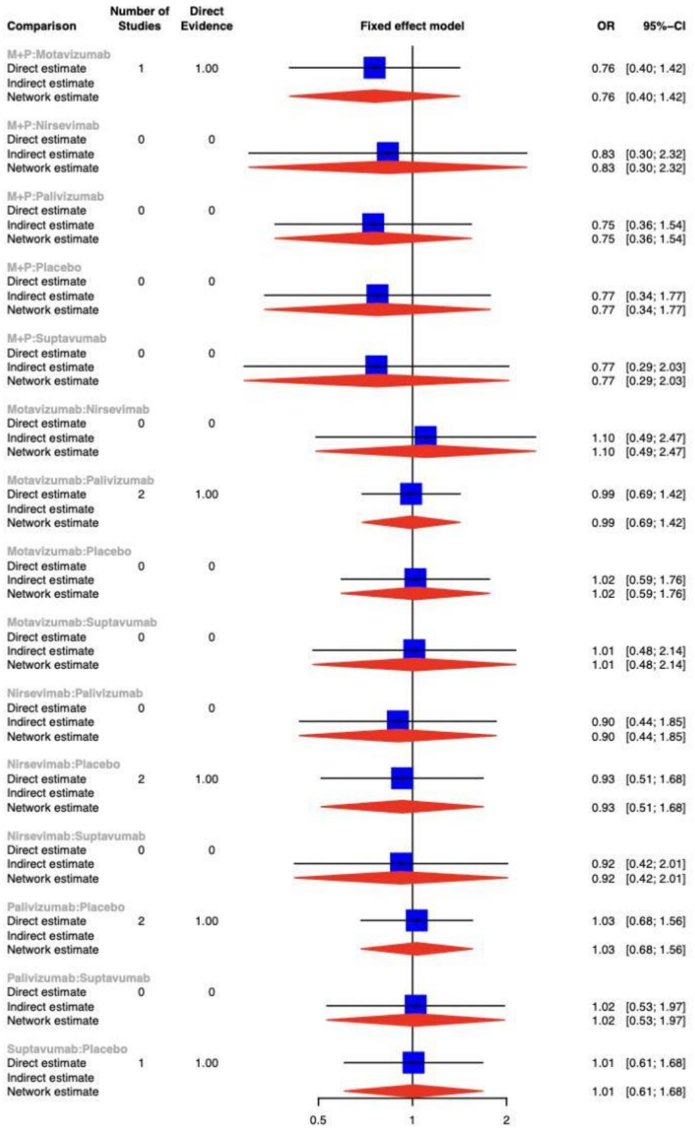

Appendix figure S48: node-split plot of drug-related adverse events in fixed effects model

© 2023 Sun M et al. *JAMA Network Open*.

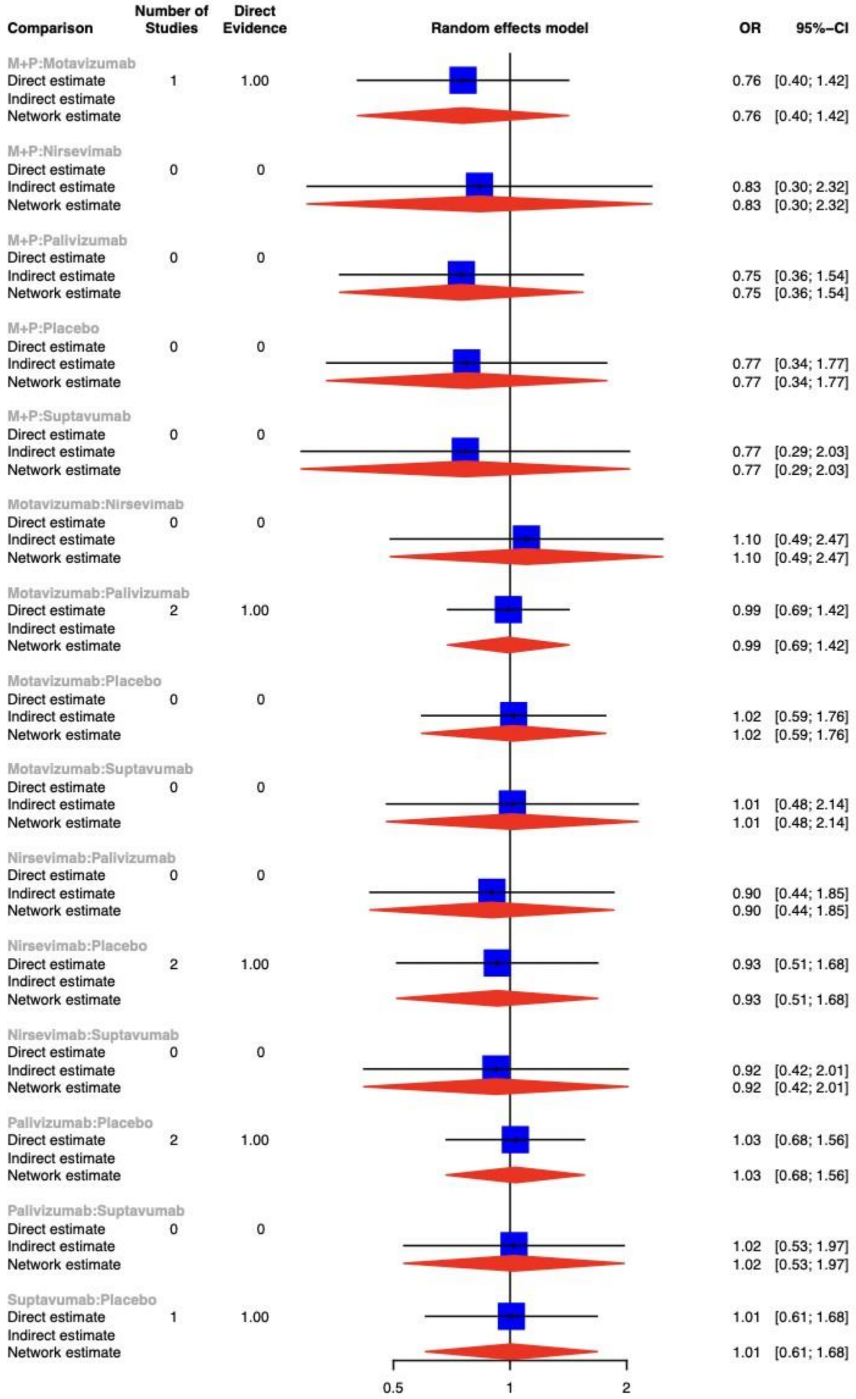

Appendix figure S49: node-split plot of drug-related adverse events in random effects model

© 2023 Sun M et al. *JAMA Network Open*.

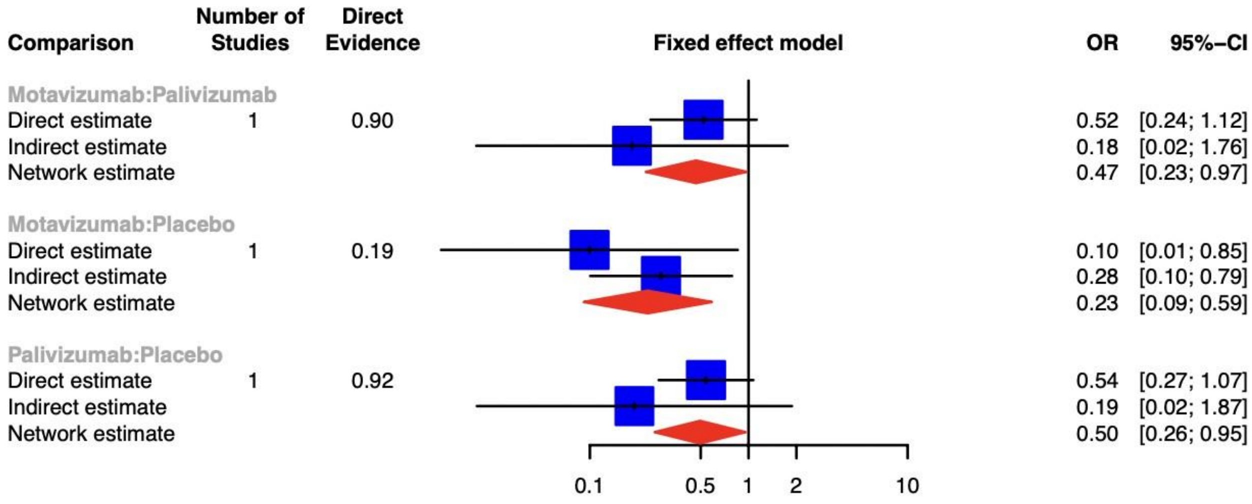

Appendix figure S50: node-split plot of rate of ICU admission in fixed effects model

© 2023 Sun M et al. *JAMA Network Open*.

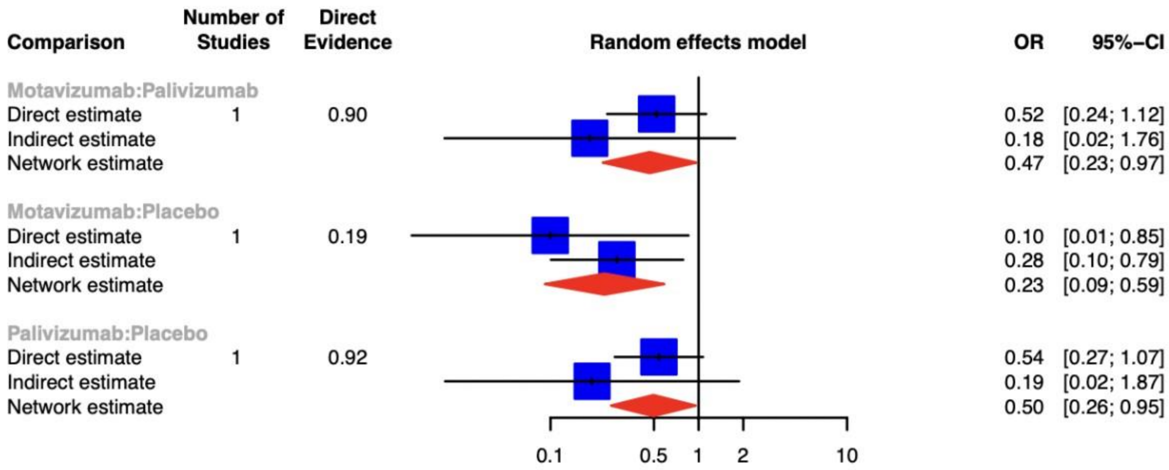

Appendix figure S51: node-split plot of rate of ICU admission in random effects model

© 2023 Sun M et al. *JAMA Network Open*.

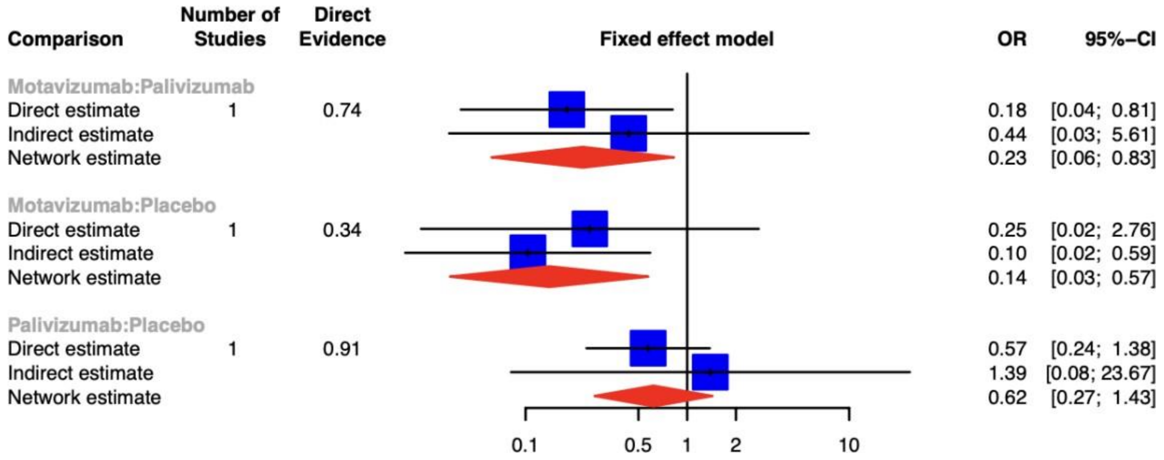

Appendix figure S52: node-split plot of rate of mechanical ventilation use in fixed effects model

© 2023 Sun M et al. *JAMA Network Open*.

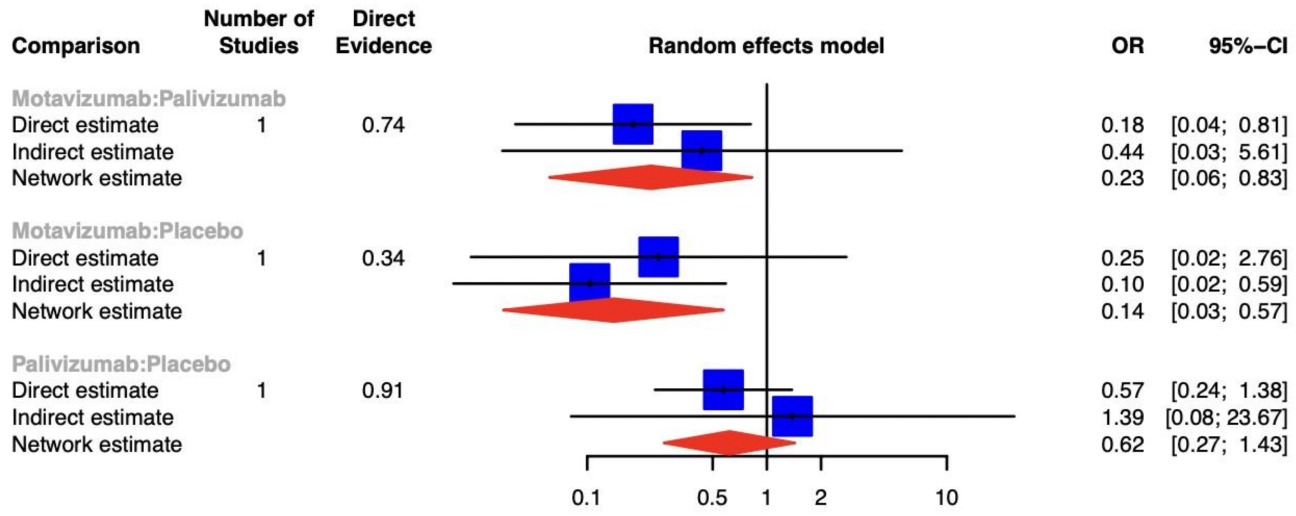

Appendix figure S53: node-split plot of rate of mechanical ventilation use in random effects model

© 2023 Sun M et al. *JAMA Network Open*.

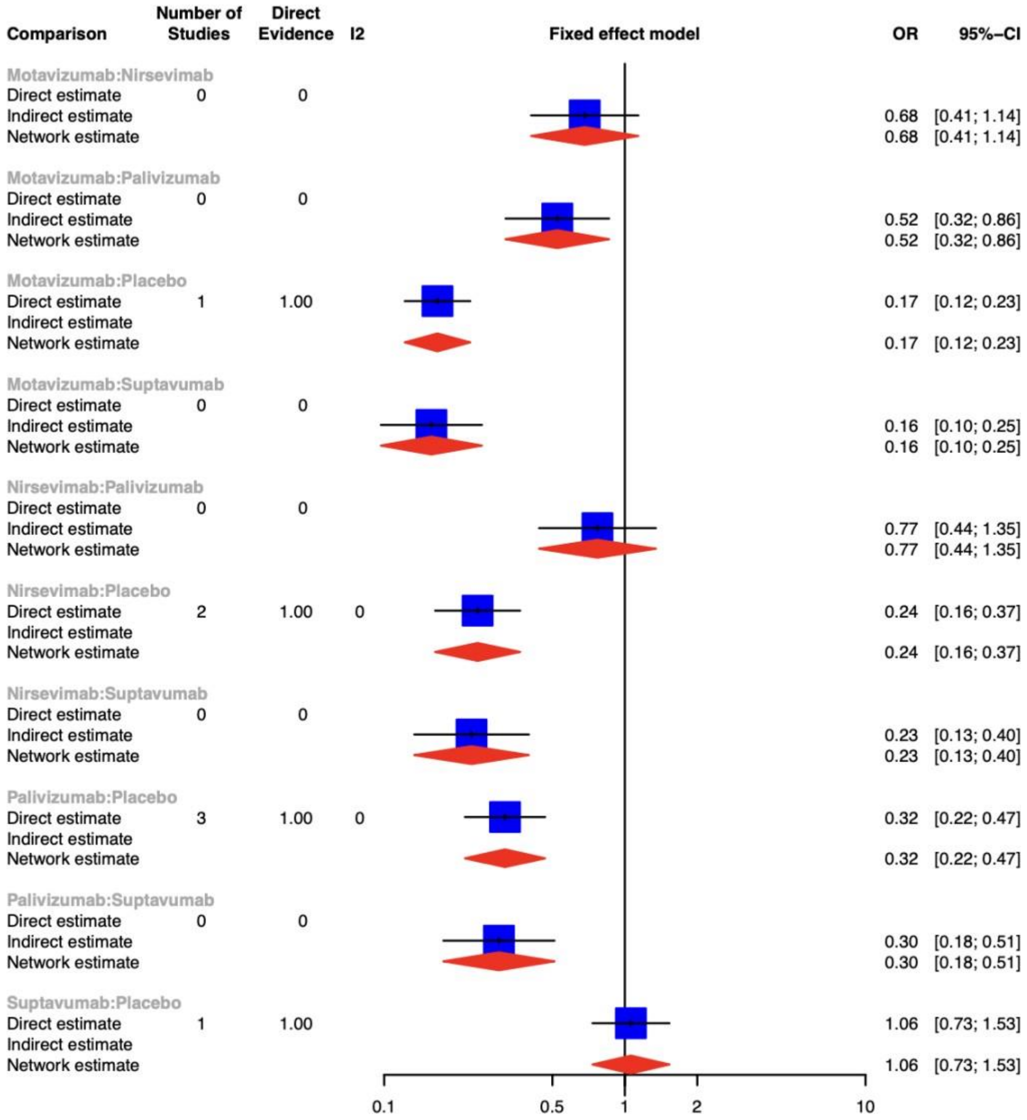

Appendix figure S54: node-split plot of rate of RSV infection in fixed effects model

© 2023 Sun M et al. *JAMA Network Open*.

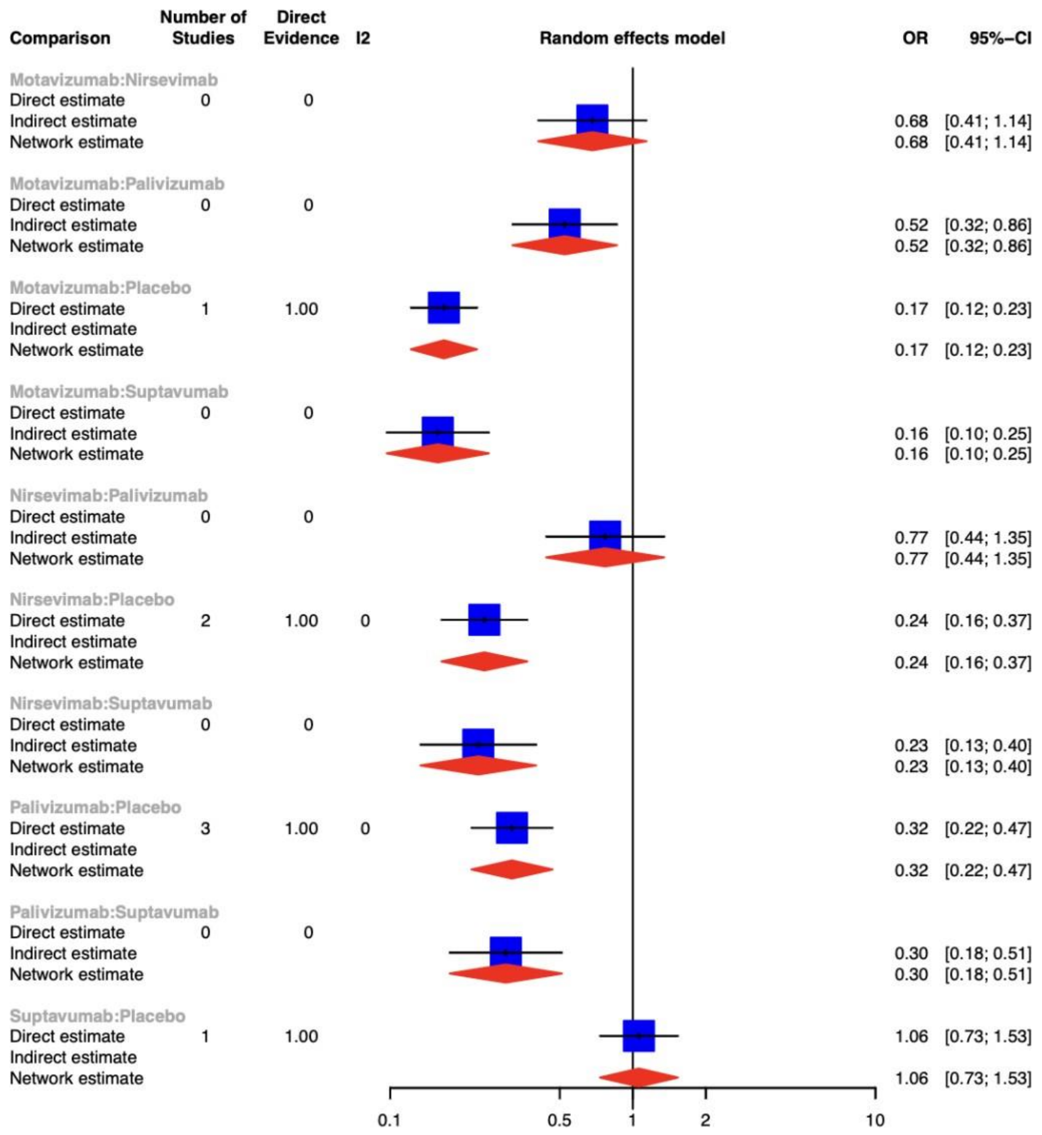

Appendix figure S55: node-split plot of rate of RSV infection in random effects model

© 2023 Sun M et al. *JAMA Network Open*.

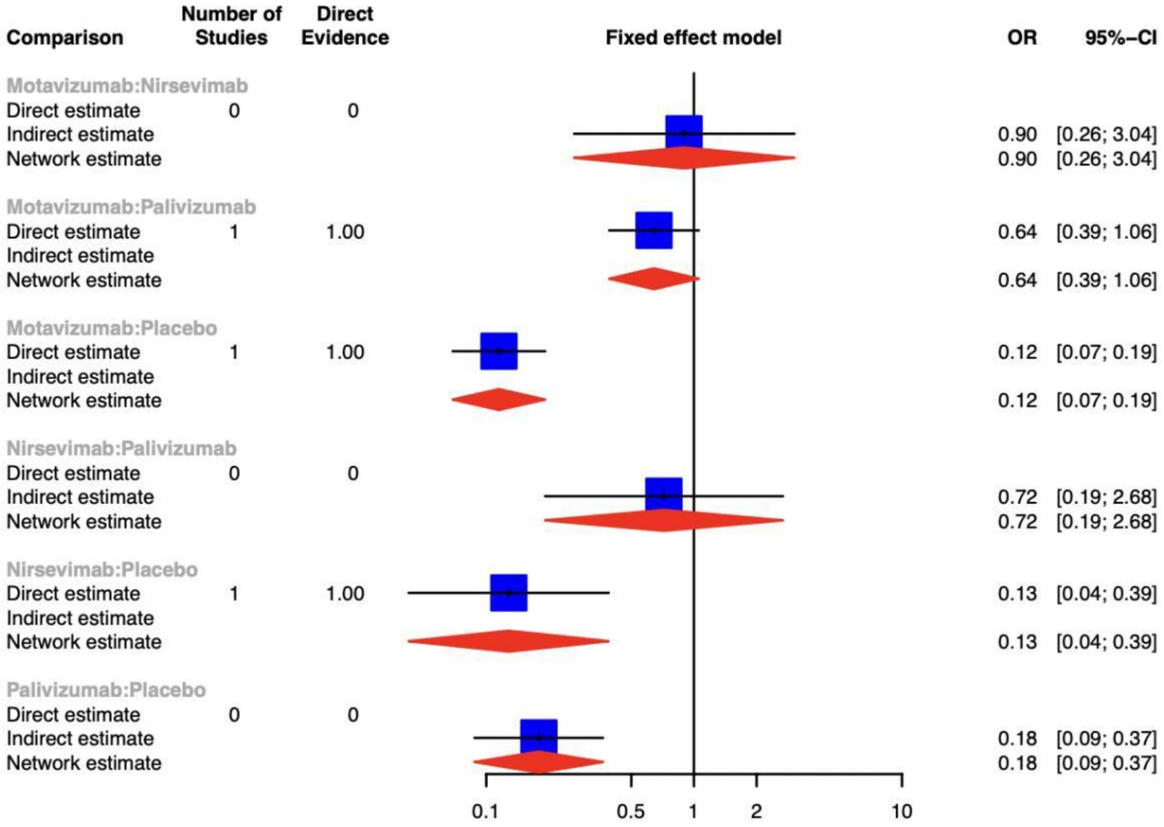

Appendix figure S56: node-split plot of rate of supplemental oxygen use in fixed effects model

© 2023 Sun M et al. *JAMA Network Open*.

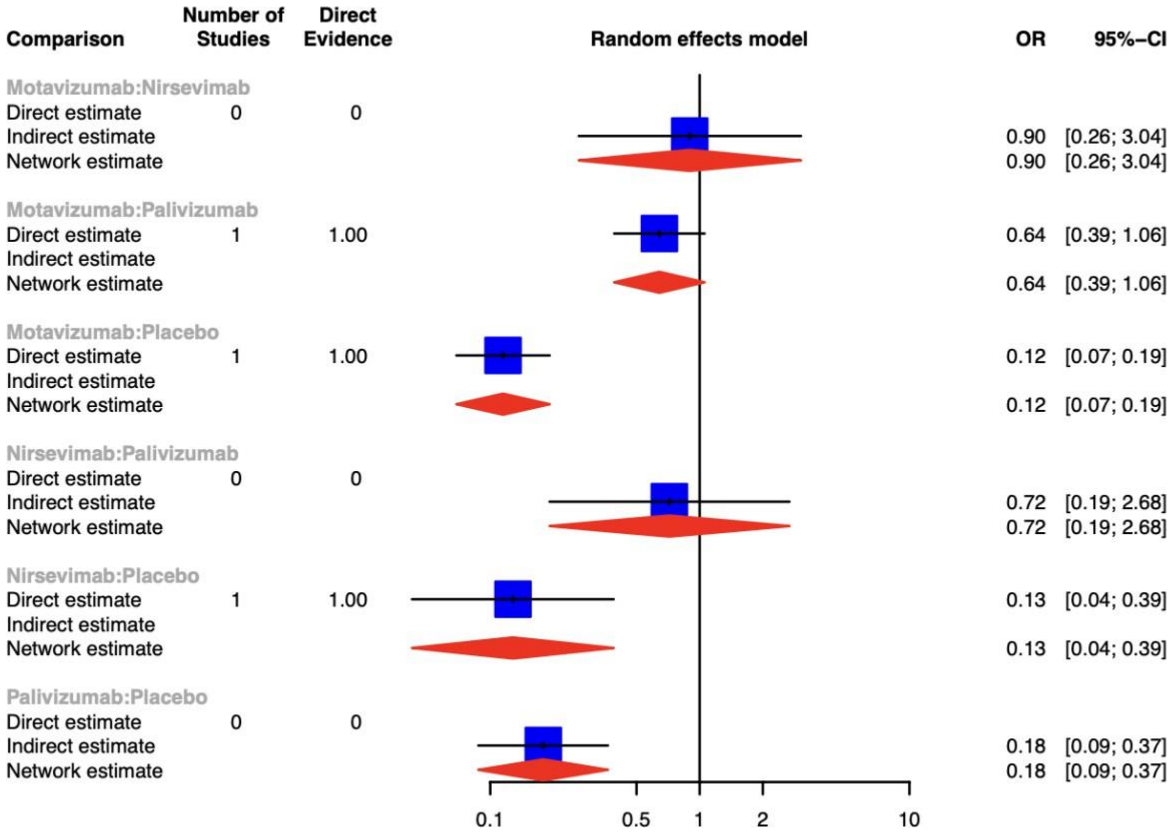

Appendix figure S57: node-split plot of rate of supplemental oxygen use in random effects model

© 2023 Sun M et al. *JAMA Network Open*.

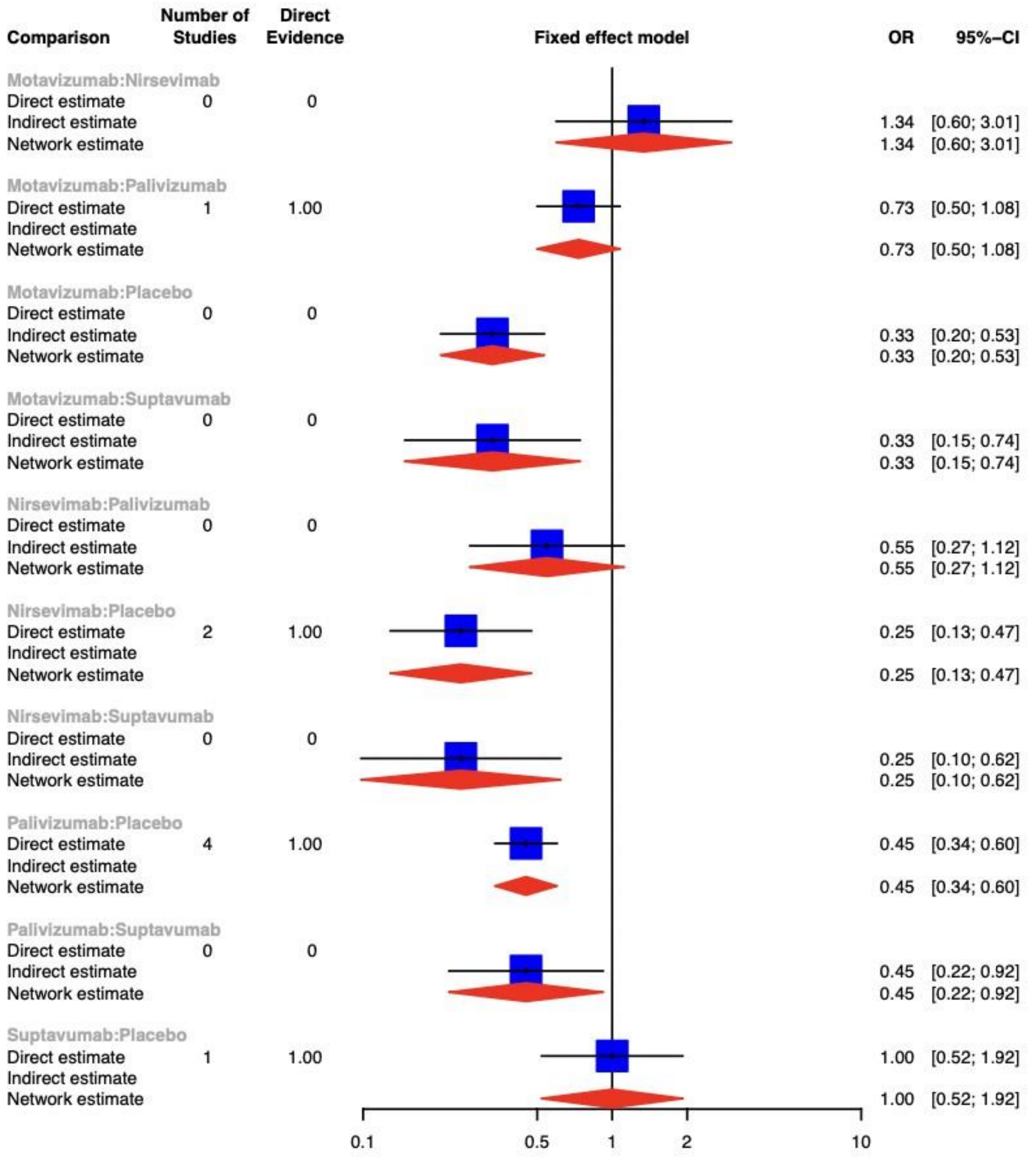

Appendix figure S58: node-split plot of RSV-related hospitalization in fixed effects model

© 2023 Sun M et al. *JAMA Network Open*.

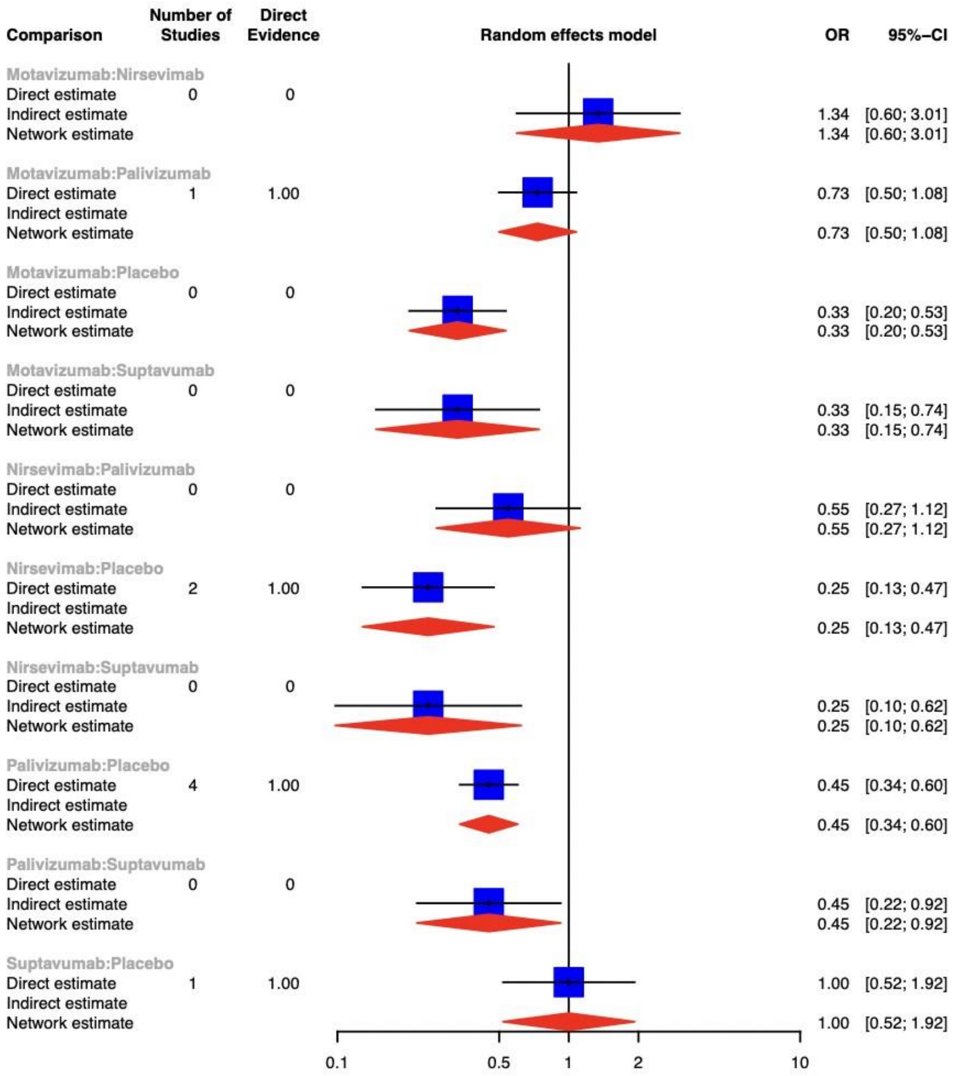

Appendix figure S59: node-split plot of RSV-related hospitalization in random effects model

© 2023 Sun M et al. *JAMA Network Open*.

Appendix figure S60-S66: contribute plots

The contribution matrix shows how much each direct treatment effect contributes to each treatment effect estimate from network meta-analysis. The rows represent network treatment effects and columns represent the contribution of direct treatment effects.

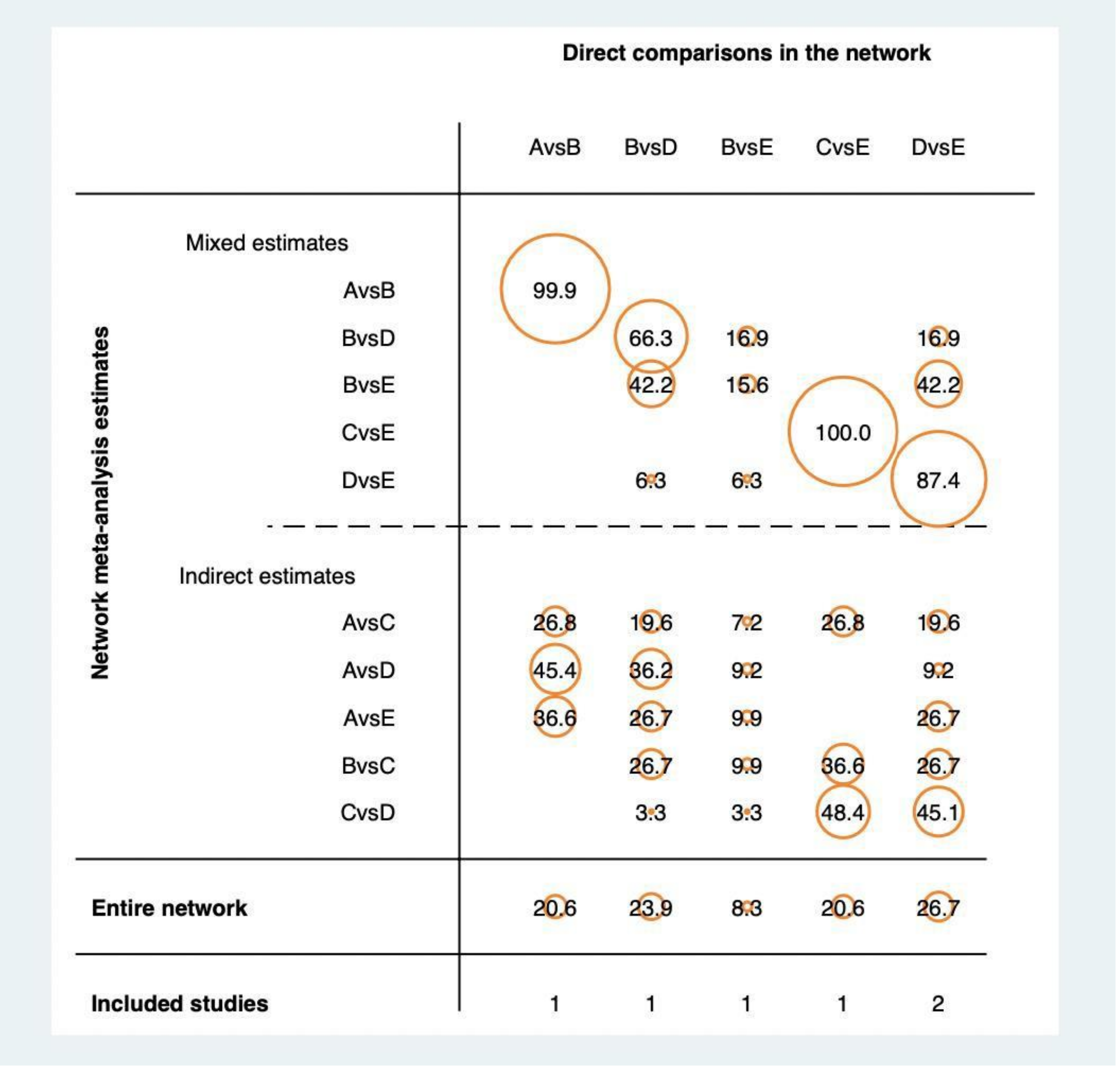

Appendix figure S60: contribute plot of all-cause mortality A: M+P; B: Motavizumab; C: Nirsevimab; D: Palivizumab; E: Placebo

© 2023 Sun M et al. *JAMA Network Open*.

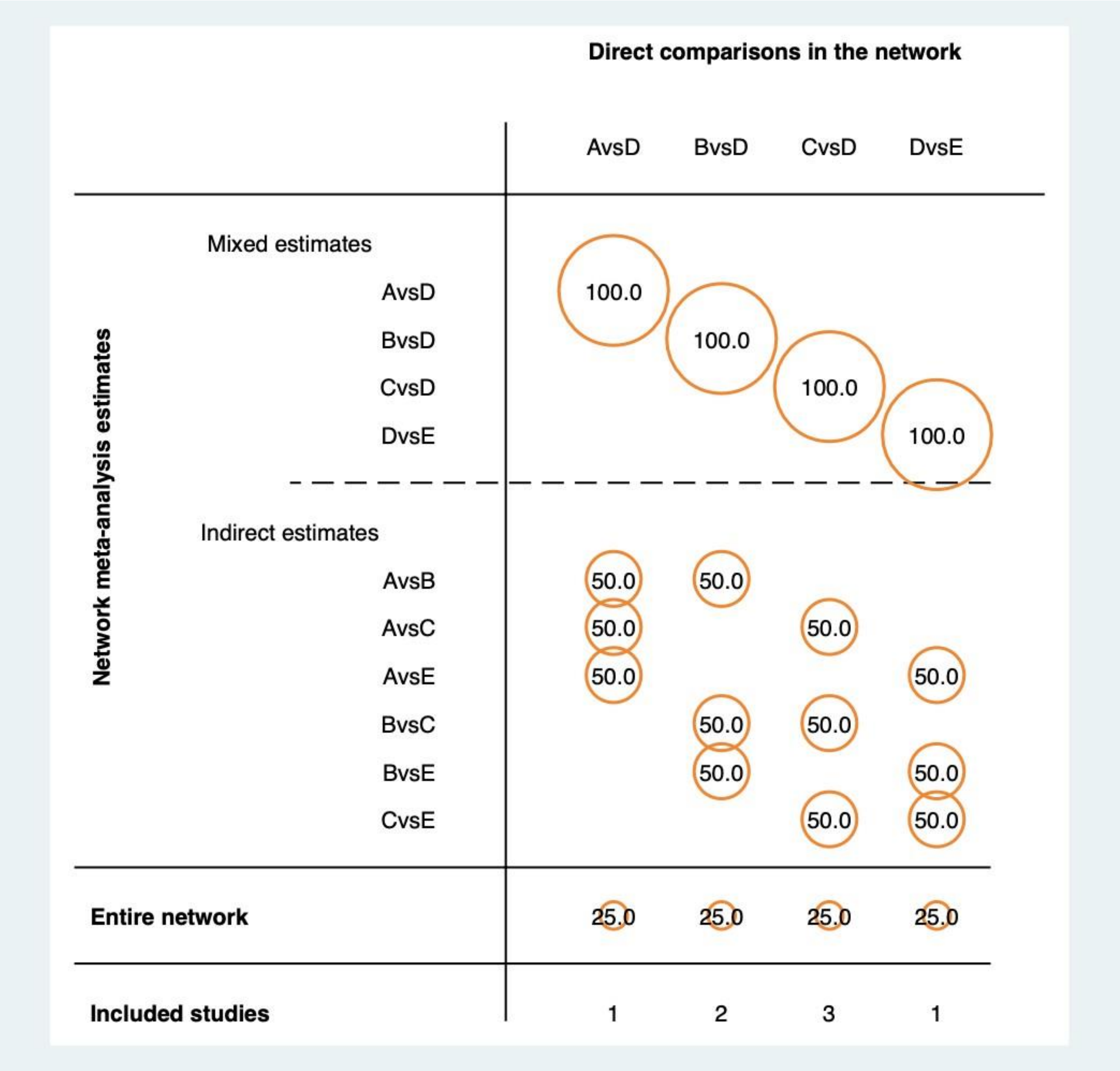

Appendix figure S61: contribute plot of rate of RSV infection A: Motavizumab; B: Nirsevimab; C: Palivizumab; D: Placebo; E: Suptavumab

© 2023 Sun M et al. *JAMA Network Open*.

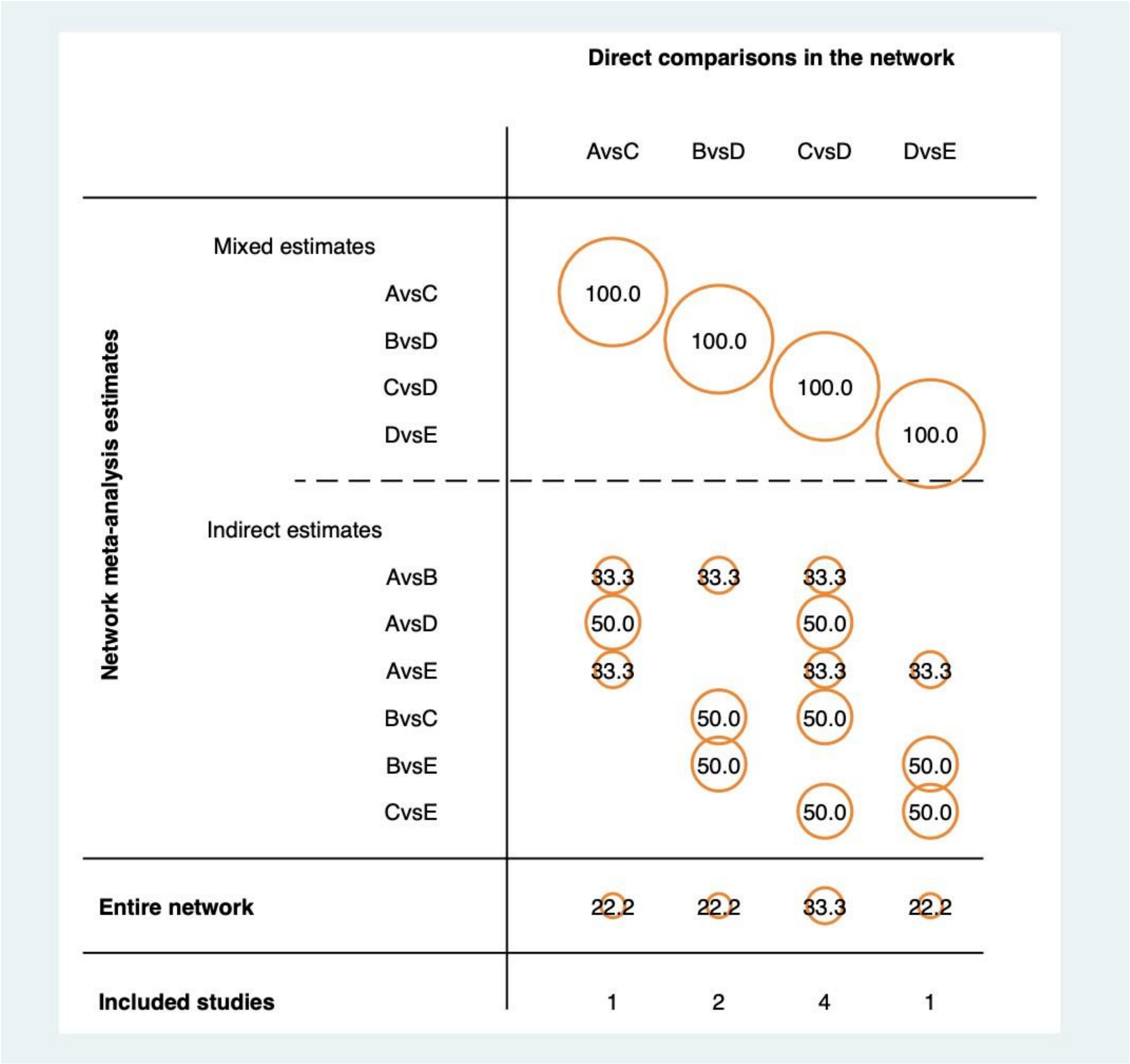

Appendix figure S62: contribute plot of RSV-related hospitalization A: Motavizumab; B: Nirsevimab; C: Palivizumab; D: Placebo; E: Suptavumab

© 2023 Sun M et al. *JAMA Network Open*.

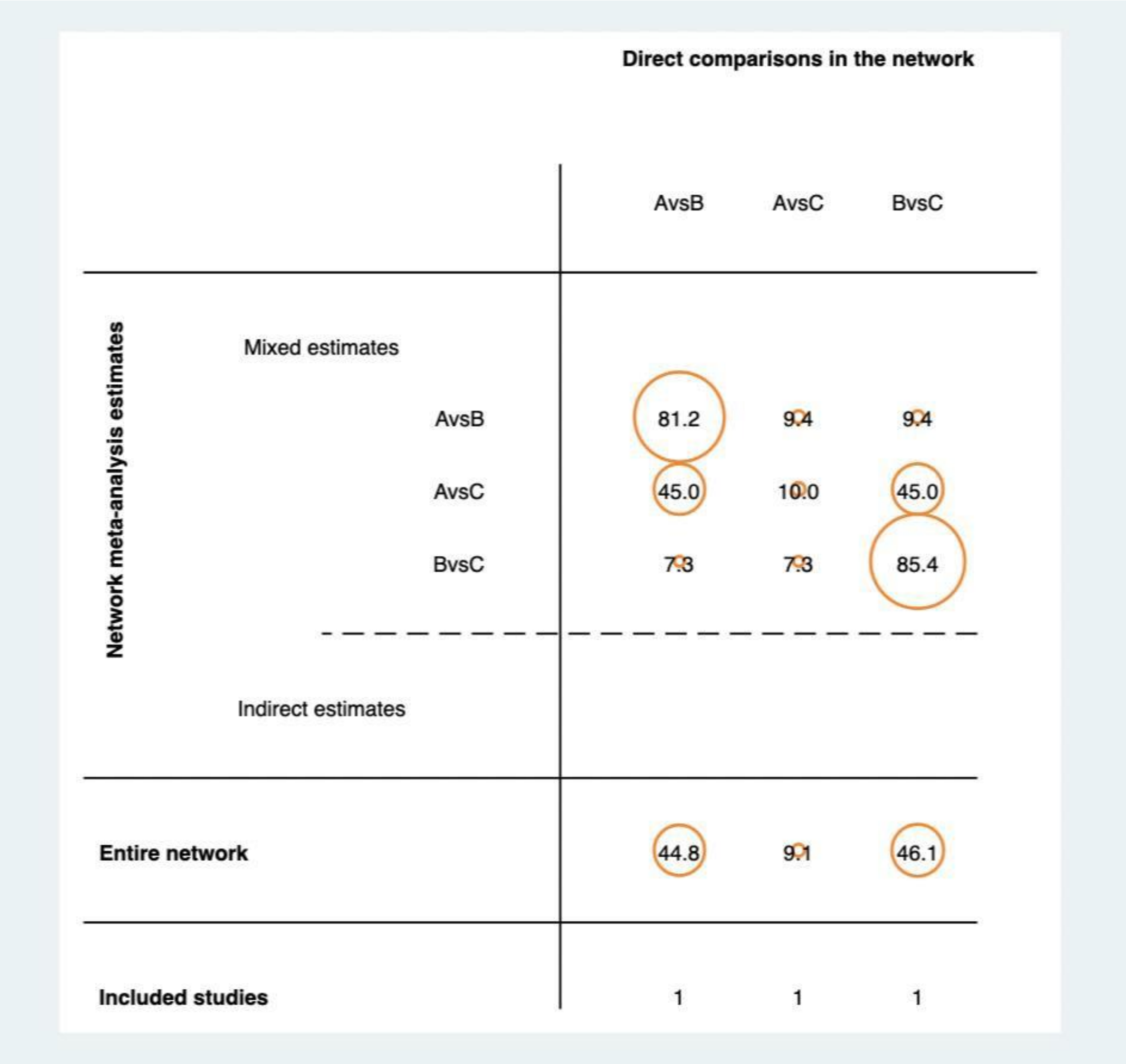

Appendix figure S63: contribute plot of rate of ICU admission A: Motavizumab; B: Palivizumab; C: Placebo

© 2023 Sun M et al. *JAMA Network Open*.

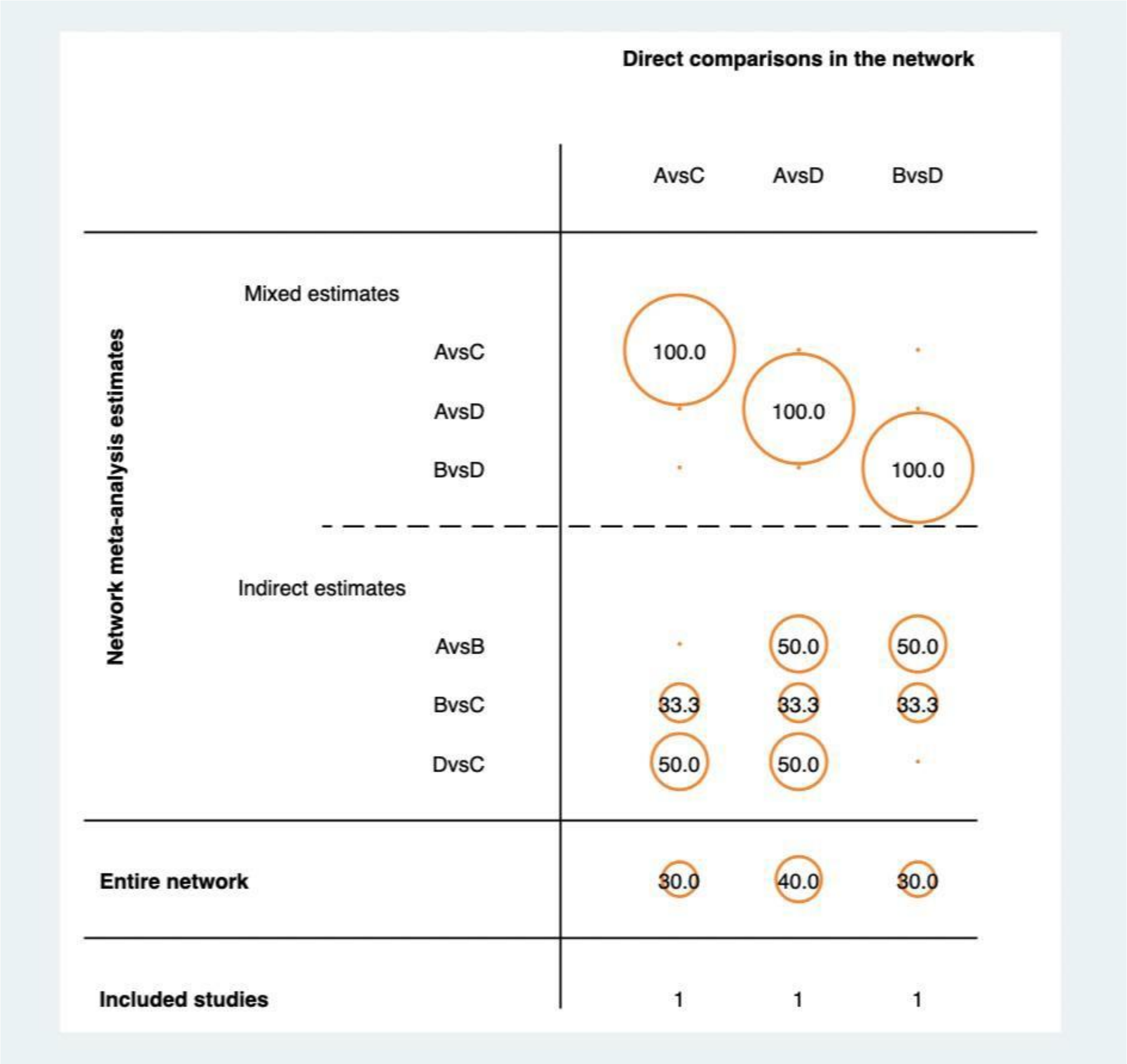

Appendix figure S64: contribute plot of rate of supplemental oxygen use A: Motavizumab; B: Nirsevimab; C: Palivizumab; D: Placebo

© 2023 Sun M et al. *JAMA Network Open*.

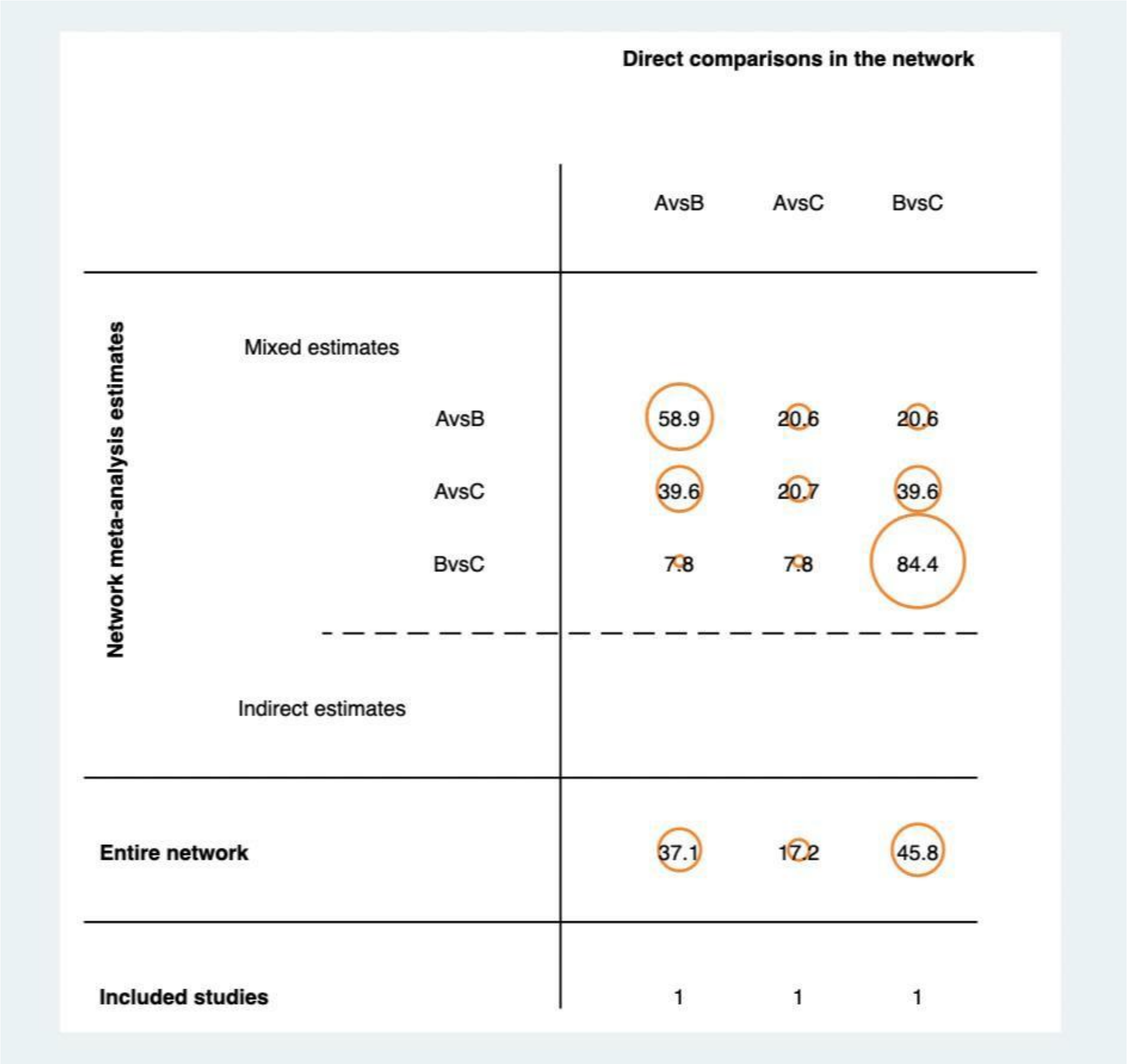

Appendix figure S65: contribute plot of rate of mechanical ventilation use A: Motavizumab; B: Palivizumab; C: Placebo

© 2023 Sun M et al. *JAMA Network Open*.

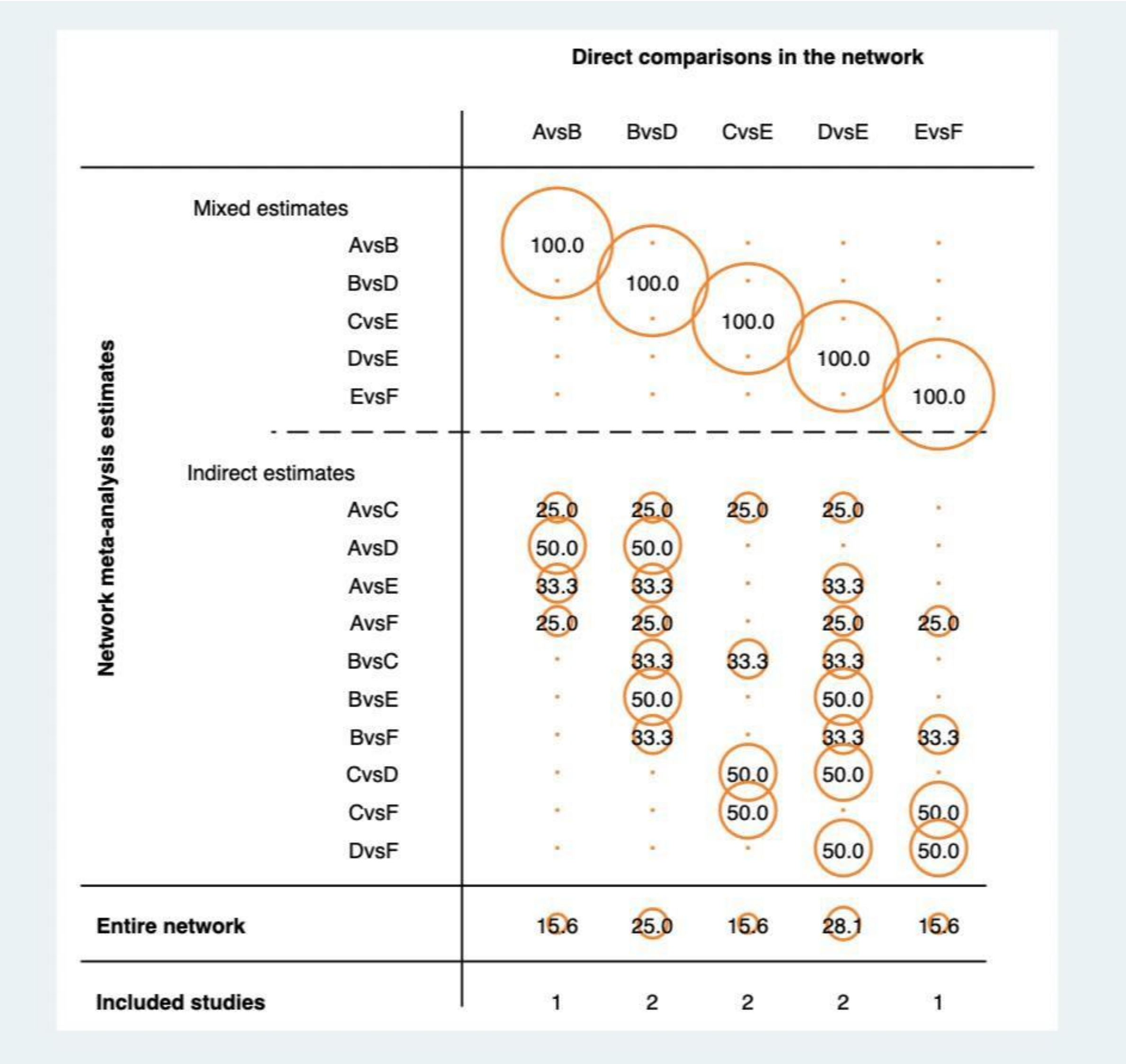

Appendix figure S66: contribute plot of drug-related adverse events A: M+P; B: Motavizumab; C: Nirsevimab; D: Palivizumab; E: Placebo; F: Suptavumab

© 2023 Sun M et al. *JAMA Network Open*.

Appendix figure S67-S68: subgroup analysis

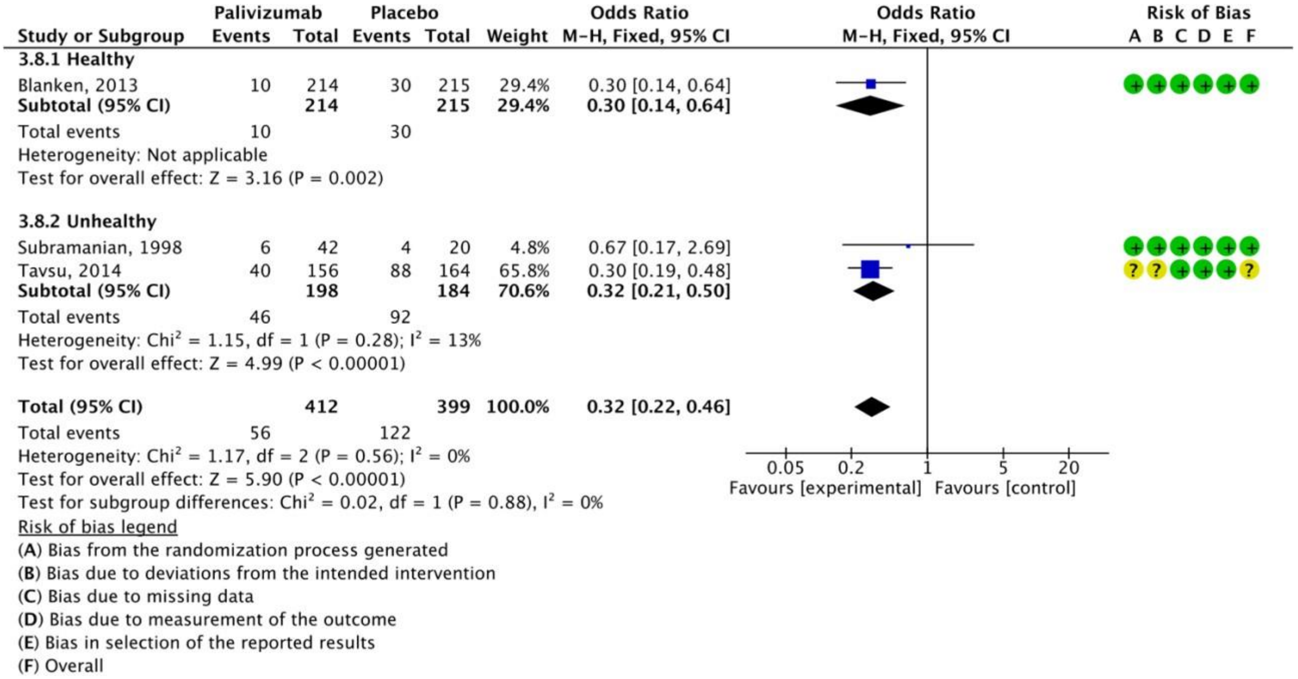

Appendix figure S67: subgroup analysis of rate of RSV infection

© 2023 Sun M et al. *JAMA Network Open*.

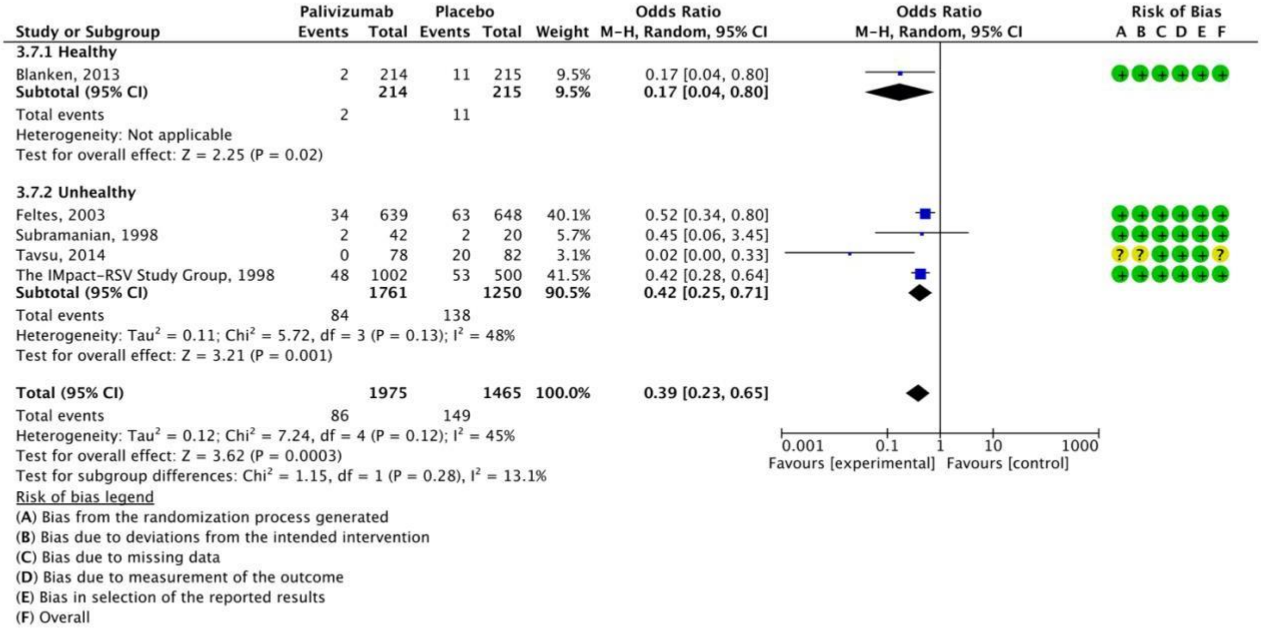

Appendix figure S68: subgroup analysis of RSV-related hospitalization

© 2023 Sun M et al. *JAMA Network Open*.

Appendix figure S69-S71: plots of meta-regression

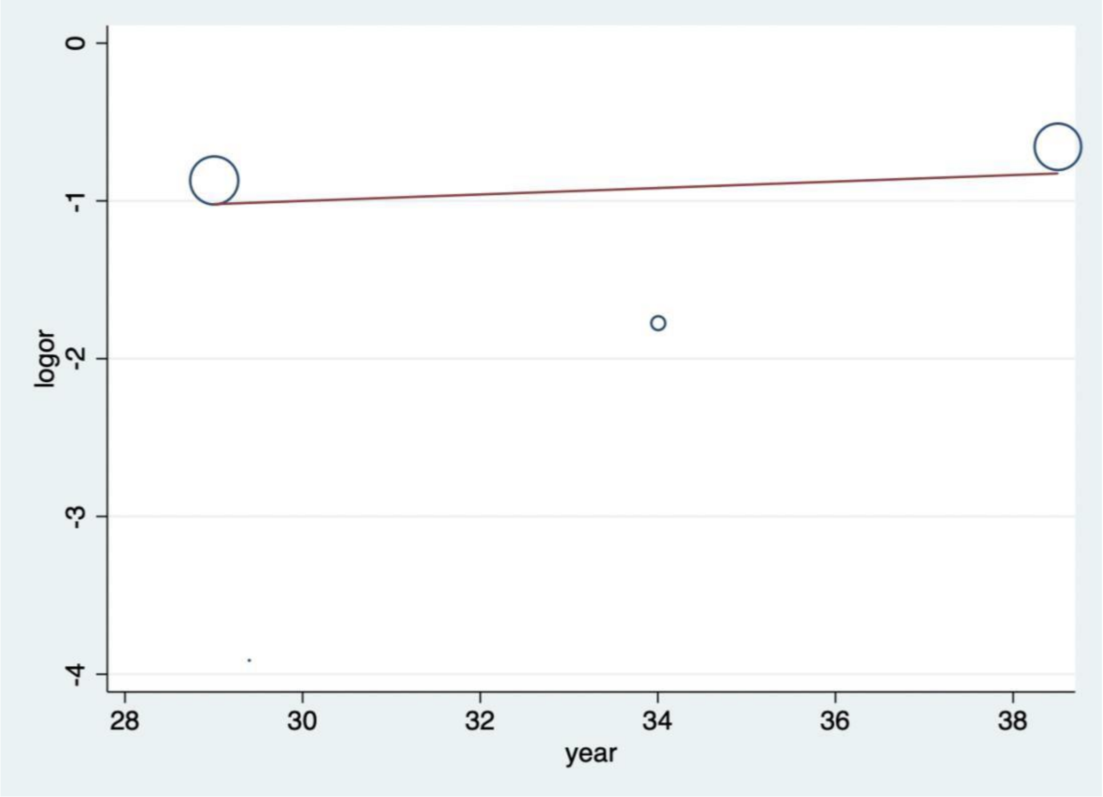

Appendix figure S69: meta-regression of age in RSV-related hospitalization

© 2023 Sun M et al. *JAMA Network Open*.

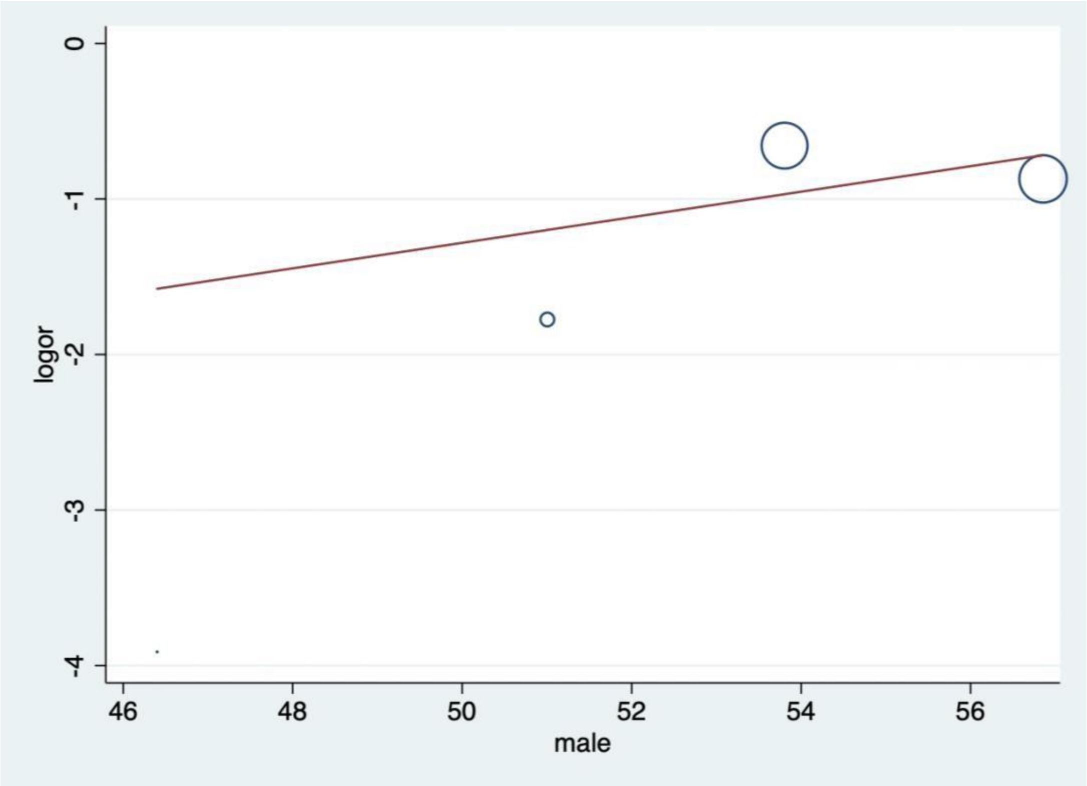

Appendix figure S70: meta-regression of gender in RSV-related hospitalization

© 2023 Sun M et al. *JAMA Network Open*.

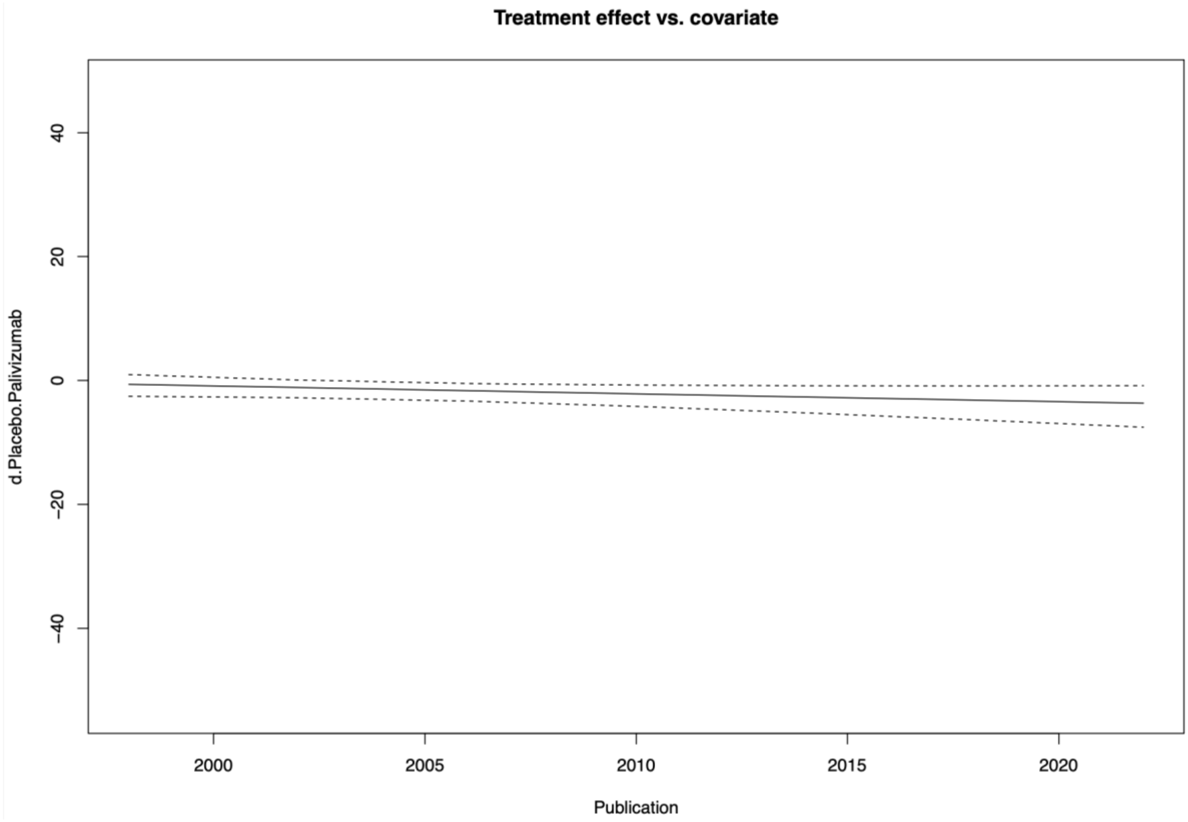

Appendix figure S71: meta-regression of publication year in RSV-related hospitalization

© 2023 Sun M et al. *JAMA Network Open*.

Appendix figure S72: Comparison of different monoclonal antibodies on efficacy and safety outcomes

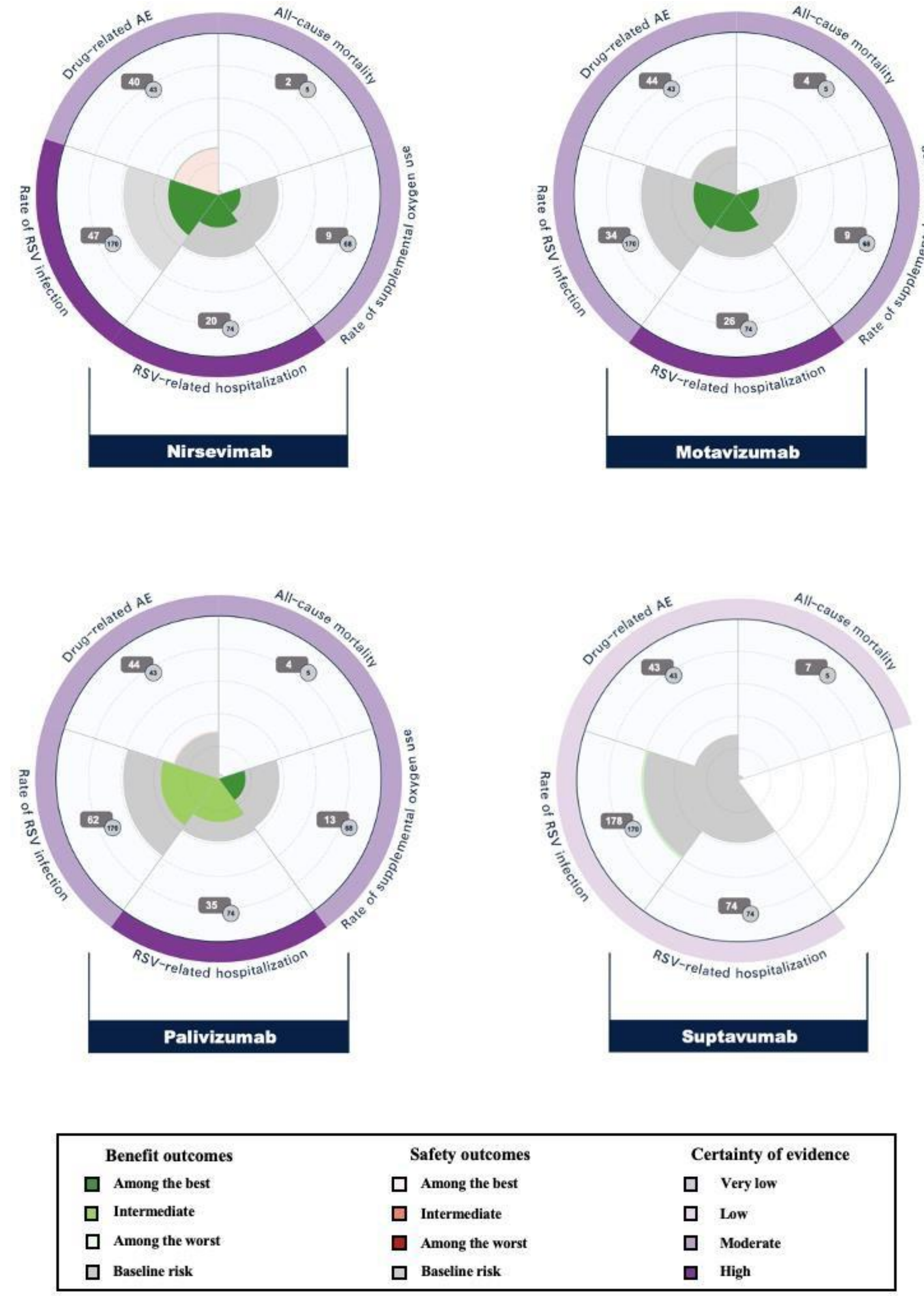

*The numbers in the circles (light gray) represent the baseline risk, the numbers in the boxes represent the absolute risk of the outcome after using the intervention. For example, the baseline risk of RSV infection was 170 per 1000 participants (light gray), after using nirsevimab, the absolute risk was reduced to 47 per 1000 participants (dark green), which indicates that nirsevimab was the best of the four drugs.

*AE: adverse events.

© 2023 Sun M et al. *JAMA Network Open*.
