# Supplementary material for: Monoclonal Antibody for the Prevention of Respiratory Syncytial Virus in Infants and Children: A Systematic Review and Network Meta-analysis
Source: JAMA Netw Open. 2023 Feb 17;6(2):e230023. doi: 10.1001/jamanetworkopen.2023.0023 (PMC9938429; doi:10.1001/jamanetworkopen.2023.0023)
Supplement: Supplement 2. — Data Sharing Statement [file jamanetwopen-e230023-s002.pdf]

Data Sharing Statement

Sun. Monoclonal Antibody for the Prevention of Respiratory Syncytial Virus in Infants and Children. *JAMA Netw Open*. Published February 17, 2023.
doi:10.1001/jamanetworkopen.2023.0023

Data
Data available: No
